# Non-destructive classification of unlabeled cells: Combining an automated benchtop magnetic resonance scanner and artificial intelligence

**DOI:** 10.1371/journal.pcbi.1010842

**Published:** 2023-02-21

**Authors:** Philipp Fey, Daniel Ludwig Weber, Jannik Stebani, Philipp Mörchel, Peter Jakob, Jan Hansmann, Karl-Heinz Hiller, Daniel Haddad

**Affiliations:** 1 Fraunhofer Institute for Integrated Circuits IIS, Development Center X-ray Technology, Würzburg, Germany; 2 Experimental Physics V (Biophysics), Julius-Maximilians-Universität Würzburg, Würzburg, Germany; 3 University of Applied Sciences Würzburg-Schweinfurt, Schweinfurt; Stowers Institute for Medical Research, UNITED STATES

## Abstract

In order to treat degenerative diseases, the importance of advanced therapy medicinal products has increased in recent years. The newly developed treatment strategies require a rethinking of the appropriate analytical methods. Current standards are missing the complete and sterile analysis of the product of interest to make the drug manufacturing effort worthwhile. They only consider partial areas of the sample or product while also irreversibly damaging the investigated specimen. Two-dimensional T_1_ / T_2_ MR relaxometry meets these requirements and is therefore a promising in-process control during the manufacturing and classification process of cell-based treatments.

In this study a tabletop MR scanner was used to perform two-dimensional MR relaxometry. Throughput was increased by developing an automation platform based on a low-cost robotic arm, resulting in the acquisition of a large dataset of cell-based measurements. Two-dimensional inverse Laplace transformation was used for post-processing, followed by data classification performed with support vector machines (SVM) as well as optimized artificial neural networks (ANN). The trained networks were able to distinguish non-differentiated from differentiated MSCs with a prediction accuracy of 85%. To increase versatility, an ANN was trained on 354 independent, biological replicates distributed across ten different cell lines, resulting in a prediction accuracy of up to 98% depending on data composition.

The present study provides a proof of principle for the application of T_1_ / T_2_ relaxometry as a non-destructive cell classification method. It does not require labeling of cells and can perform whole mount analysis of each sample. Since all measurements can be performed under sterile conditions, it can be used as an in-process control for cellular differentiation. This distinguishes it from other characterization techniques, as most are destructive or require some type of cell labeling. These advantages highlight the technique’s potential for preclinical screening of patient-specific cell-based transplants and drugs.

## Author summary

Stem cells are of great importance for scientific and clinical research. Modern approaches seek to treat degenerative diseases with stem cell therapies. For these applications, it is of central interest to ensure that the cells have developed into the cell or tissue type required for the respective treatment. To this end, a small number of cells or tissues are typically sacrificed to perform routine staining or genetic analysis, and the results are generalized to the entire sample. This study presents an alternative method based on magnetic resonance that is capable of classifying cells in the entirety of the culture. Since only lightweight and mobile components were included, this application is highly flexible and can be used in a routine laboratory environment. With the help of a self-developed robotic platform a large database of cell-related measurements was created. Using an artificial neural network, it was possible to accurately follow the developmental process of mesenchymal stromal cells and distinguish between differentiated and undifferentiated cells with an accuracy of 85%. Furthermore, it was possible to distinguish between multiple different cell lines with an accuracy of up to 98% depending on the sample composition.

## Introduction

The increasing importance of advanced therapy medicinal products (ATMPs) for the treatment of various diseases such as atherosclerosis, muscle and tendon disorders or skin ulcers [1,2] leads to a need for more suitable characterization methods. Before being transferred to patients, ATMPs must undergo rigorous testing [3]. In particular, if a graft is based on differentiating cells such as mesenchymal stromal cells (MSCs), successful maturation of the corresponding cells and tissue must be demonstrated. Usually, this process requires sacrificing small portions of the product to perform routine staining. Considering the high cost and effort for isolation, cultivation and possible differentiation of the corresponding cells, a suitable characterization method should either use as little material as possible or better as much as possible but in a non-destructive way so that the product can subsequently be applied to the patient.

Histological assessment usually relies on trained personnel performing the sample assessment. As this gives rise to possible human errors, multiple other studies have also introduced unbiased, computer-led methods to support the fast and unbiased diagnosis process in a variety of clinical applications [4–7]. Although there are other established methods for the identification and characterization of cells and tissues—such as Raman spectroscopy [8–12] or single cell RNA sequencing [13,14]—besides classical histological and immunofluorescent staining, they cannot be performed under sterile conditions or lead to sample destruction. Moreover, they consider only a small portion of the final graft and depend on the homogeneity of the mature cells. Magnetic resonance (MR) relaxometry promises the possibility of non-destructive whole-sample analysis under sterile conditions and overcomes the above limitations. The data obtained is a two-dimensional representation of the T_1_ and T_2_ relaxation times of the respective samples [15–17]. The relaxation times represent the sample’s physical properties, based on its molecular composition. This results in shorter T_1_ values for samples with a higher water content compared to samples containing more lipids. Depending on the applied MR pulse sequence, different physical properties of the measured samples can be investigated.

The underlying MR sequence and processing steps used in this work were originally proposed by Vekataramanan et al. and Song et al. in 2002 [18,19]. This data acquisition combines an inversion recovery (IR) sequence for T_1_ quantification and a Carr-Purcell-Meiboom-Gill (CPMG) sequence [20,21] for T_2_ quantification (for further details on the used sequence please refer to S1 Fig and the materials and methods section). Data reconstruction methods developed later facilitated rapid and accurate fitting of the acquired data [22,23].

The recorded relaxation times, when applied globally, can be used to create a fingerprint based on the respective values and distributions. The recorded data represent multiple physical properties at once. Therefore, it is not possible to extract specific tissue information like density, elasticity, or viscosity from the results. MR relaxometry has been used in biomedical and biochemical contexts to characterize water diffusion in skeletal muscle cells [24] and human tissues such as liver, kidney, and articular cartilage [25]. Subsequent studies have successfully demonstrated how to increase the measurement speed [26,27] and have succeeded in demonstrating the interaction between water and biopolymers such as proteins [28].

The aim of this study was to provide proof of principle that MR relaxometry can indeed provide suitable results for non-destructive, high-precision, and objective classification and identification of various cells and cell development and possibly in future applications, even complex tissues such as biopsies. This will support pre-clinical and clinical unbiased decision making leading to cost-reduction for expensive assays as well as increasing patient safety by supporting the diagnosis based on objective data.

Since their therapeutic potential and importance are widely recognized and used in clinical applications [29–32], MSCs were selected as a suitable model system to investigate the ability of MR relaxometry to monitor the cellular differentiation process. As previously described, aqueous and fatty solutions produce characteristic relaxation times which differ significantly from each other. Therefore, adipogenic differentiation was chosen for the MSCs in this study, as it should show the clearest differences in MR signal. Because primary cells, such as MSCs, can yield widely varying results depending on the original tissue and donor [33–35], cell lines were also included in this study as a more standardizable method for characterization. This was particularly important because a large dataset was needed for the targeted AI classification, which required a reproducible model system.

All measurements were performed using a mobile low-field benchtop MR scanner so that the presented methodology can be used in any standard cell culture laboratory. To increase the throughput and thus the number of measurements, a low-cost automation solution was developed based on open-source technology such as the Arduino microcontroller. For data and thus cell classification, support vector machines (SVM) as well as customized artificial neural networks (ANN) were trained on the acquired data, allowing fast and accurate assignment to the appropriate developmental stage or cells.

Based on previous studies [28,36], the presented work investigates the hypothesis, that 2D T_1_ / T_2_ magnetic resonance relaxometry combined with artificial intelligence can be used to identify and characterize unlabeled cells in a non-destructive and sterile way.

## Results

### Proving measurement stability and reproducibility of the tabletop MR scanner

**Fig 1:**
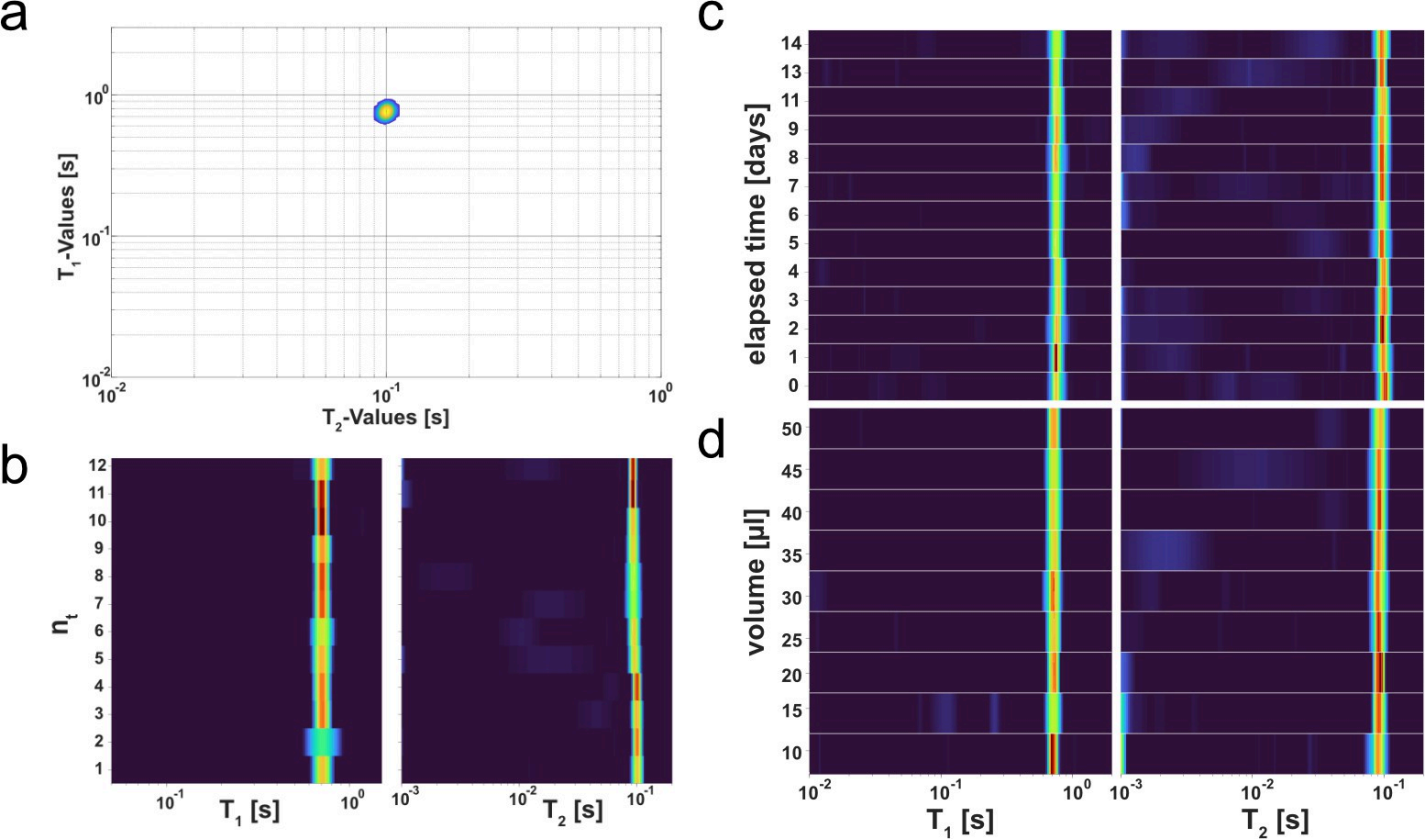
Validation of signal stability by using an agarose Dotagraf cell phantom (ADCP). An ADCP was used—consisting of RPMI 1640 cell-culture medium with 0.25 mM of Dotagraf for T_2_ adjustment and 0.6 wt% of agarose for T_1_ adjustment—for generating a MR phantom showing similar T_1_ / T_2_ values compared to exemplary measurements of cells (**a**). The ADCP was used to prove the signals short-term reproducibility by measuring twelve separate samples in a row and comparing their T_1_ and T_2_ spectra (**b**). In addition, the long-term reproducibility was proven by measuring multiple ADCP samples throughout a 14-day time course (**c**). Using the ADCP samples, it was also investigated how the sample’s volume would influence the generated data (**d**). Within the given range of 10 μl to 50 μl the signal showed little to no variation, indicating a volume independent signal.

In order to produce a defined and reproducible measurement, it was decided to establish a phantom sample to test the MR setup. A custom MR phantom was created to match the data obtained on Chinese hamster ovary (CHO) cells. The corresponding measurements were later included in Fig 3. For this, a combination of the gadoteric acid-based contrast agent Dotagraf and agarose dissolved in Roswell Park Memorial Institute (RPMI) cell-culture medium was used. The concentration of both components was adjusted so that the produced T_1_ / T_2_ distribution (**Fig 1**A) matched the one from the measured CHO cells. With this defined agarose-dotagraf cell phantom (ADCP) multiple measurements were performed to investigate the system’s reproducibility.

Twelve individual ADCP samples were measured from one stock solution. The T_1_ and T_2_ spectra were plotted, stacked above each other (**Fig 1**B). Here it became apparent, that the peaks fluctuated in width and height within a certain degree, but that the position of the peaks stayed consistent throughout all repetitions. This observation was congruent in both dimensions (T_1_ and T_2_). To investigate the long-term reproducibility of the tabletop scanner, a series of samples were measured daily over a period of two weeks (**Fig 1**C). For each point in time, several samples were measured, and the resulting signal was combined to reflect the overall trend of the corresponding time point. The data showed the previously described fluctuation around a constant position in T_1_ and T_2_, proving that the measured values remained constant over time. Statistical analysis confirmed the initial visual interpretation of the data (T_1_ p-value = 0.0932; T_2_ p-value = 0.1807; S4A Fig).

The ADCP was used to investigate the influence of the sample’s volume on the produced T_1_ / T_2_ signal. For this, samples with varying volume of ADCP from 10 μl to 50 μl in 5 μl increments were prepared. The experiment was repeated three times with freshly prepared ADCPs for each repetition. Like in **Fig 1**C, the values for each repetition were combined to reflect the general tendency of the respective sample point. The results showed consistent T_1_ and T_2_ positions for the measured data throughout all recorded samples (**Fig 1**D), indicating that the acquired T_1_ / T_2_ distribution was independent of the corresponding volume. Like in the previous section, statistics confirmed the initial visual interpretation of the plotted data (T_1_ p-value = 0.9736; T_2_ p-value = 0.7419; S4B Fig).

### Automated cell measurement enabled the establishment of a comprehensive data pool

**Fig 2:**
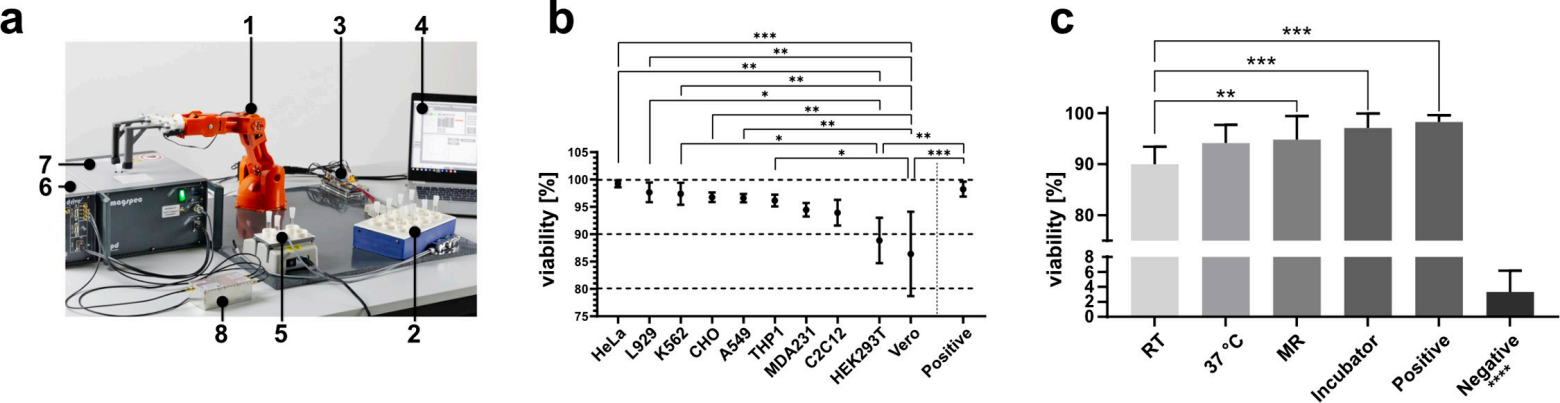
The evaluation of the automated measurement process proves the viability of the processed cells. Automation of the measurement process facilitated the efficient generation of a comprehensive dataset that could be used for analysis. For this purpose, a low-cost robotic arm (a-1) was combined with a self-designed and 3D-printed sample holder (a-2; respective STL files can be found in the attachment). The automation platform was controlled by a microcontroller (a-3) connected to a laptop running a self-designed Matlab user interface (a-4). To keep the viability of the samples as high as possible, they were preheated to 37°C in a heating block (a-5). The measurement system consisted of a console (a-6), a temperature-controlled neodymium magnet operating at 37°C (a-7), and a low-noise amplifier (LNA) (a-8). Subsequent assessments of the viability of three biological replicates (n_b_) showed a significant decrease only for HEK293T and Vero cells. Compared with the positive control, none of the other cells showed a statistically significant decrease in viability (b). When the root cause of this decrease was investigated, it was found that the only culture factor that showed significant differences was when the cells were kept at room temperature (RT) for 5 hours (c). This leaves the undersupply of cell culture medium, in addition to keeping the sample at 37°C, as the only factor to improve culture conditions for future applications and development. All samples showed highly significant differences compared to the negative control. All measurements were carried out using n_b_ = 3. *: P ≤ 0.05 / **: P ≤ 0.01 / ***: P ≤ 0.001 / ****: P ≤ 0.0001; n_b_: biological replicates.

Once it had been demonstrated that the measurement method provided reproducible data, it was of central importance for the subsequent analysis to provide the AI algorithms with a sufficiently large pool of measurement data. The only way to achieve this in a timely manner was to automate the measurement process. Therefore, a low-cost robotic arm was combined with self-designed 3D-printed parts to build an autonomously functioning platform (Fig 2A; the STL files for 3D printing can be found in the attachment).

In pre-measurement studies, it was determined, that the samples should contain 5e6 cells, as this yielded reliable results and could be obtained from most cell cultures (S3 and S4C Figs). Therefore, every cell related sample was composed of this number of cells. To verify their viability, cells were processed in the automation platform and then measured using the NucleoCounter NC-200 as an unbiased, automated counting method. Three biological replicates (n_b_) were measured for each cell line used. Statistical analysis of the data showed that only two of the ten measured cell lines (HEK293T and Vero) exhibited a significant decrease in viability (Fig 2B). All other cell lines did not show significant differences when compared to the positive control harvested immediately after the cells were harvested from the culture flask. An examination of the possible influences of culture conditions on the viability of the samples revealed that they showed a decrease in viability when stored at RT for the duration of 5 hours (the maximum time the samples would spend in the measurement cycle). All other factors studied did not result in a significant change in viability (Fig 2B). This was most evident when comparing the results with a positive control in which the cells were prepared in the same manner but kept in an excess amount of culture medium and stored in the incubator for the duration of the assay. The reported observations suggest that the undersupply of culture medium may be responsible for the reduced viability.

The NucleoCounter not only provided information on cell viability, but also an estimate of the cell’s diameter. Although the diameters showed significant differences (S4A Fig), these did not correlate with the observed changes in viability. CHO cells had the largest diameter, i.e., they yielded the largest cell sample at a given cell number and thus the least amount of excess medium, but still had one of the highest observed viabilities. The opposite can be assumed for the Vero cells, which had the smallest diameter, i.e., the largest amount of excess medium, but showed the lowest cell viability of all cell lines examined. This indicated that cell diameter did not have a significant effect on cell viability after processing.

### Evaluation of the generated dataset based on the shape and position of the measured MR spectra

Since this study aimed to demonstrate that the presented method can be used to non-invasively discriminate between different cell types, it was decided to perform most measurements based on commonly used cell lines (S8 Fig). Although they represent a biologically stable and reproducible model system, they are of little relevance for application in a clinical context. Therefore, patient-derived mesenchymal stromal cells (MSCs) were differentiated into adipocytes (S8 Fig). Both stages, undifferentiated and differentiated cells, were measured.

**Fig 3:**
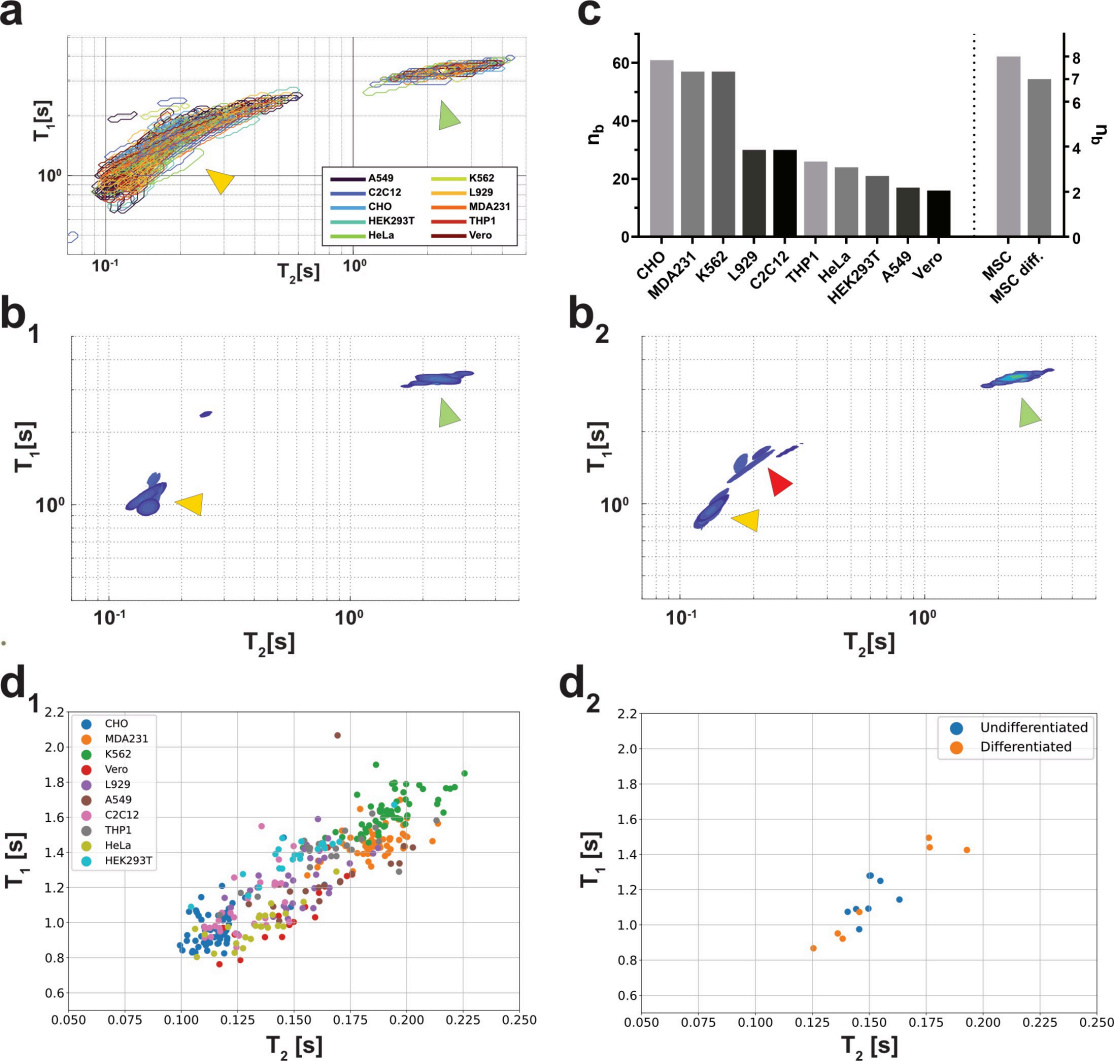
The evaluation of the measured data showed an accumulation of signal peaks at different T_1_ and T_2_ times. When contour plots were plotted for the measured 2D spectra, two major signal clusters became apparent. One was referred to as the cell peak (yellow arrowhead) and one as the medium peak (green arrowhead). For the cell lines, these clusters were indistinguishable at first glance (a), whereas the MSC clusters showed only one cell peak for undifferentiated cells (b-1 –yellow arrowhead; n_b_ = 8) and two cell peaks for differentiated cells (b-2 –yellow and green arrowhead; n_b_ = 7). The total dataset included n_b_ = 362 (cell lines: n_b_ = 354; MSCs: n_b_ = 8) with a total of 369 samples, as MSCs were measured twice, once for the undifferentiated stage and once for the differentiated stage (c). For experimental reasons, the measured dataset was not balanced, meaning that not all cells were measured equally often. To reduce sample complexity, the weighted centroid was calculated for each cell peak and plotted as a singular T_1_ / T_2_ coordinate (d1—cell lines; d2—MSCs). Here, the clustering of the different cell lines and MSC differentiation stages became most apparent. This clustering provided the first evidence on how to distinguish the different cells based on their spectral data. n_b_: biological replicates.

Using the automation solution described previously, a dataset was created that included 362 independent measurements. For cell lines (n = 354), each measurement represented a biological replicate. For MSCs, eight biological replicates were measured, with one contaminated during differentiation, ultimately resulting in 15 independent measurements for MSC-associated data (Fig 3C). When plotting all spectral data for the measured cell lines, the signal clustered in two distinct locations. One was referred to as the media peak (Fig 3A; green arrowhead), while the other peak was referred to as the cell peak (Fig 3A; yellow arrowhead), as the latter only occurred when cells were added to the samples (S2 Fig). While the spectra for the measured cell lines showed very different cell peaks due to their shape and location (Figs 3A and S7), the spectra obtained for undifferentiated (Fig 3B1) and differentiated (Fig 3B2) MSCs were more locally specific. Another difference was that the cell lines and undifferentiated MSCs showed only one cell peak (yellow arrowhead), while the differentiated MSCs showed two (red arrowhead). This might be due to the lipid buildup during the differentiation process (S8 Fig). As described in the introduction, lipids usually show significantly different MR behaviors compared to aqueous solutions like cells and cell culture media.

To reduce the complexity of the signals, the spectra were reduced to only one T_1_ / T_2_ coordinate by calculating the respective weighted centroid. When these were plotted for the measured cell lines (Fig 2D1) and the MSCs (Fig 2D2), the respective regions were more distinguishable for each cluster (S6 Fig). As these were in distinct T_1_ / T_2_ regions, this provided initial evidence that the weighted centroid of the cell peak could provide suitable information for non-invasive classification.

### Using a support vector machine as a first approach to classify cells based on data obtained from MR measurement

**Fig 4:**
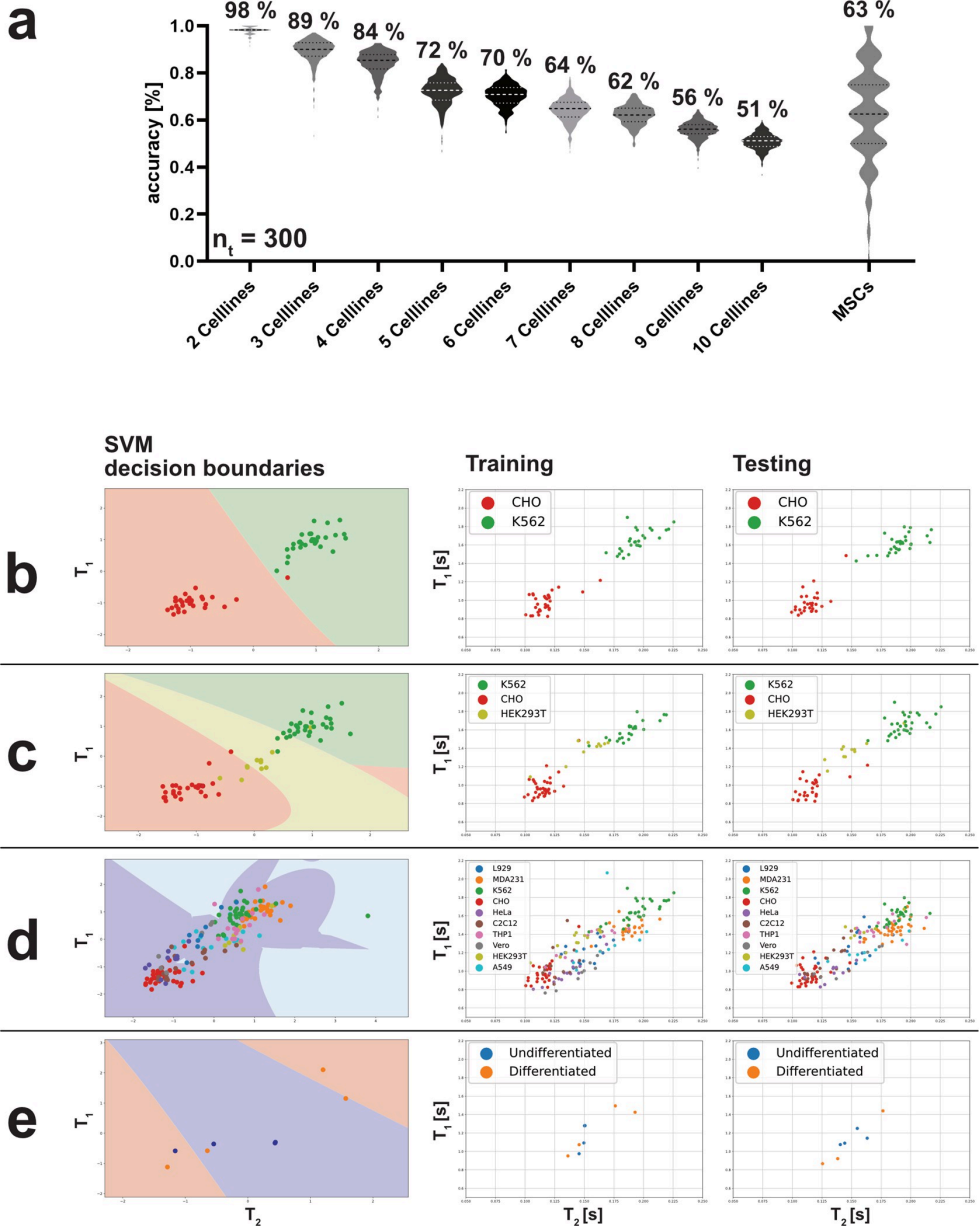
The Support Vector Machine (SVM) provided promising classification results based on the weighted centroid of its cell peaks. When training an SVM on the calculated weighted centroid of different combinations of cell lines, the accuracies obtained correlated directly with the number of cell lines included in the data (a). With the exception of 5 vs. 6, 7 vs. MSC, and 8 vs. MSC, all possible combinations yielded significant differences. The decision boundaries showed that the SVM had difficulties classifying the cell lines correctly (b-d; S9 and S10 Figs). For each iteration, the data were split into training and test data, and the final accuracy was calculated based on the test data. Because the MSC classification decision depended on two data points for each differentiated sample, rather than one, the SVM in these cases yielded widely varying results, ranging from 100% to 0%. Since the SVM calculation did not require too much computational effort, 300 technical replicates (n_t_) were calculated for each combination of cell lines.

As previously stated, the weighted centroid showed promising results to use it for classifying the cells. Therefore, the calculated weighted centroids were used as input for a support vector machine (SVM), which classifies the given data by separating them using a non-linear vector. For this, the data were randomly split into training and testing data for every run by a factor of 50%. This ensured, that the SVM was only evaluated based on previously unseen data points. In order to take the SVM’s performance with different cell lines into account, the overall dataset could be split into subsets consisting of a partial amount of cells (S1 Table). To investigate the performance, based on the composition of the dataset, each combination was tested individually and the resulting accuracies for 300 technical replicates were compared next to each other (Fig 4A). The accuracy correlated directly with the number of included cell lines. The decreasing accuracy could also be visualized by plotting the decision boundaries for the SVM (Figs 4B–4D and S9 and S10). While the results were very promising for the classification of the cell lines (Average accuracy of 300 technical replicates: 2 CL = 98.89%; 3 CL = 89.3%; 4 CL = 84.04%; 5 CL = 72.16%; 6 CL = 70.34%; 7 CL = 64.25%; 8 CL = 62.03%; 9 CL = 55.84%; 10 CL = 50.82%; CL = cell lines), the performance for the MSC classification (62.67%) was highly variable and in most cases significantly different to the cell lines. Only for the comparison of 7 cell lines and 8 cell lines to MSC, no significant changes could be detected. The decision boundaries for the MSC classification (Fig 4E), showed the algorithm’s struggle to classify MSCs, because the decision was not only based on one data point but on two datapoints simultaneously. By design, this was not a challenge that the SVM was designed for, limiting its applicability to situations where the MR data can be reduced to one individual centroid. Due this this high variability in results, the SVM did not provide a suitable machine learning approach to classify the development of MSCs.

### Augmentation of measured data to obtain enough datapoints to train an artificial neural network (ANN)

While the SVM provided suitable results for the classification of cell lines, it did not provide the expected results for the classification of differentiated vs. undifferentiated MSCs. Another aspect that needed reflecting was, that the reduction of the spectrum to just one T_1_ and one T_2_ coordinate, also lost all information on the orientation and shape of the original signal peak. To incorporate this information into the evaluation, it was decided to use artificial neural networks (ANN) as a classification approach. These incorporated not only one coordinate but the entire matrix of 300 x 300 datapoints.

**Fig 5:**
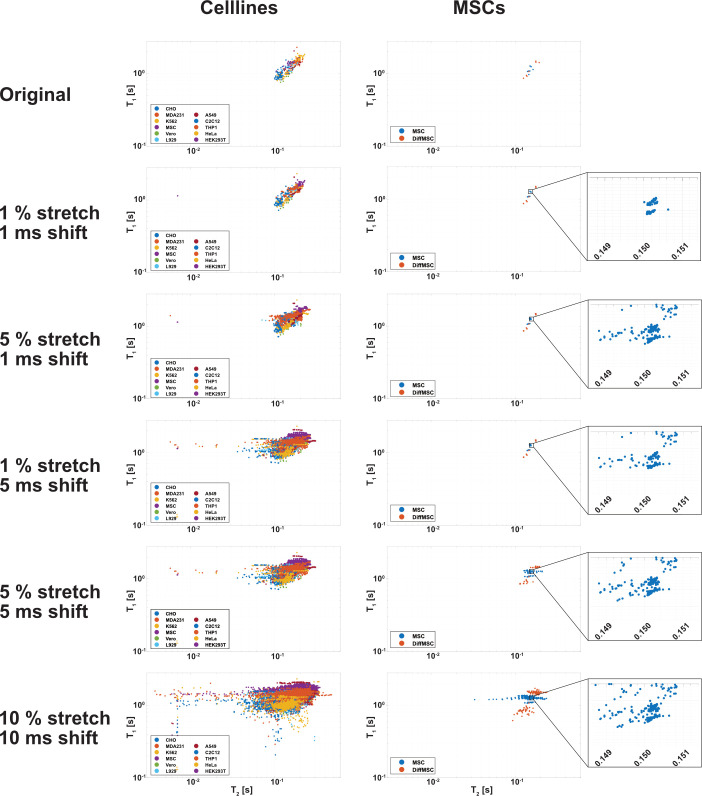
Visualization of the effect of augmentation on the data. Different combinations of increasing stretch and shift values were tested to optimize the augmentation parameters. To visualize the results, the weighted centroids for the augmented data (augmentation factor of 40) was calculated and plotted on scatter plots. This was done separately for the augmented cell line data and the MSC data. Due to the double logarithmic scaling of the x- and y-axis of the plots, the effect was more visible at T_2_ values. With increasing values, it could be shown how the originally localized signal clusters merged into overlapping regions. This merging of the data may indicate an initial limit to the augmentation values, as strong augmentation leads to indistinguishable signal clusters. Due to the small number of MSC data, the merging of augmented values is not as profound as in cell lines, not resulting in indistinguishable data.

The downside of an ANN is, that it is highly dependent on enough training data. Therefore, a self-designed augmentation algorithm was used to increase the amount of available training and testing data. The algorithm individually selected the cell peaks and was able to shift them by a random value with a definable standard deviation in T_1_ and T_2_. The algorithm was also able to stretch or shrink the selected peak by a random percentage of the original peak width (with a definable standard deviation), keeping the overall signal intensity constant, generating different shapes of signal peaks. To estimate the optimized values for augmentation, the cell line and MSC data were each augmented 40 times using different augmentation values. The weighted centroids for each augmented dataset were calculated and plotted, to visualize the effect of the augmentation on the data (Fig 5). With increasing the augmentation values (shift and stretch), the individual signal groups started to merge into each other resulting in areas of overlapping signal. This was most prominent for shift values ≥ 5 ms (standard deviation). Because of the fewer datapoints, the merging effect for the MSCs was not as prominent as for cell line data. The observed merging of the centroids gave a first possible limitation for the optimization of the augmentation parameters for the ANN training.

The overall effect of the augmentation algorithm was also made visible when the individual spectra were projected onto separate T_1_ and T_2_ axes (S11 and S12 Figs). Here, the shift of the augmented values relative to the original values could be shown more comprehensively. The stretching aspect of the augmentation was also made visible by different peak widths and heights.

The augmented data was then, together with the original data, used to train neural networks. The structure of the network was based on a modified version of the VGG network [37]. A network consisting of seven convolutional layers and four sense layers was chosen for MSC classification. Normalization and maxpooling layers were inserted between the convolutional layers. Relu was chosen as the activation function and Adamax as the optimization function (S14 Fig). For training and testing, cells were cropped to the region representing the cell peak (T_1_: 0–3.0079 s / T_2_: 0–0.4062 s).

**Fig 6:**
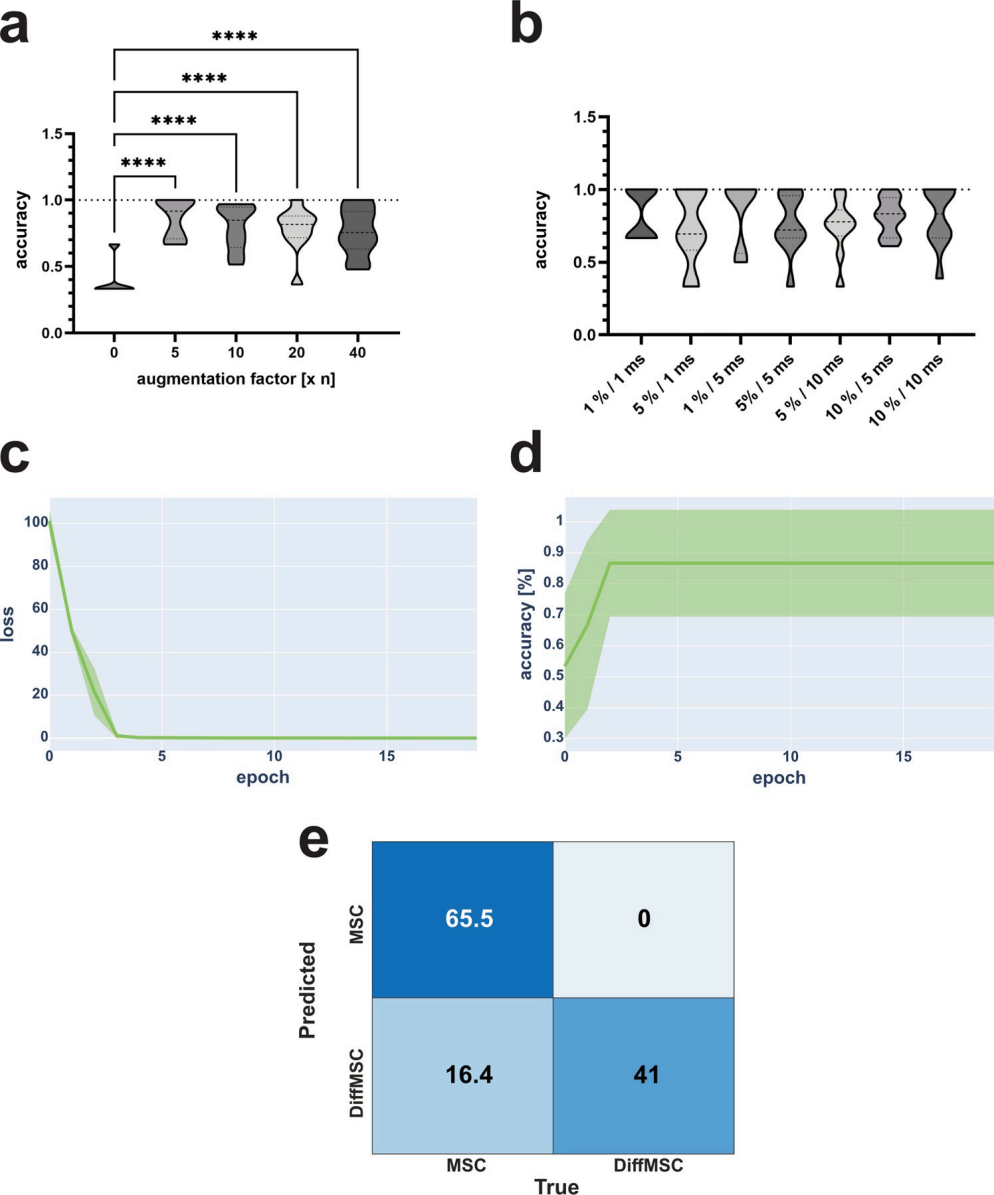
Optimization of augmentation parameters and investigation of ANN performance for training on MSC data. Examination of the effect of augmentation factor on ANN performance showed that each augmentation factor resulted in significantly higher accuracy compared to performance on un-augmented data (a—average accuracy = 40%). No significant differences were found when comparing the different augmentation factors (5: 86.67%; 10: 79.7%; 20: 78.1%; 40: 76.18%). Therefore, it was decided to use an augmentation factor of five for future analyses, since the amount of data directly correlated with the time needed for training. A similar evaluation was performed for the augmentation parameters stretch and shift (b). All combinations examined did not yield significant differences. Based on the mean accuracy, 1% stretch and 5 ms shift were chosen as the final augmentation parameters for MSC classification. Based on these scores, an ANN was trained on the corresponding dataset. The cumulative loss values (c) converged after only three to four epochs, while the validation accuracy (d) reached its plateau of 85% at the same time. While the standard deviation (shaded area) of the loss function was very small, it ranged from 100% to 70% for the validation loss, indicating a dependence of the evaluation result on the train-test assignment. The confusion matrix (e—average values of ten replicates) also proved that most of the samples were correctly classified. All measurements were performed ten times independently and the resulting values were averaged. All depicted values are based on n_t_ = 10. *: P ≤ 0.05 / **: P ≤ 0.01 / ***: P ≤ 0.001 / ****: P ≤ 0.0001.

When examining the influence of augmentation factors on ANN performance, the statistics showed that there were no significant differences between the assessed augmentation values (Fig 6A). The only significant difference was the comparison between non-augmented and augmented data. The latter yielded significantly higher accuracies. This confirmed the original assumption that the initial dataset was too small, and that augmentation was crucial for training success. Since the amount of data directly correlated with the time required for the ANN to train, the augmentation factor was set to five for further MSC-based ANN analysis.

Further evaluation of the augmentation parameters showed no significant differences between different stretch and shift values (Fig 6B). Based on the highest mean value, 1% stretch and 5 ms shift were selected as the final augmentation parameters for MSC-based ANN training (1% / 1 ms: 83.33%; 5% / 1 ms: 73.89%; 1% / 5 ms: 85%; 5% / 5 ms: 76.67%; 10% / 5 ms: 81.11%; 5% / 10 ms: 74.44%; 10% / 10 ms: 81.11%). Testing higher augmentation values showed a significant decrease in accuracy above a certain threshold. At 25% stretch and 25 ms shift, the results were not significantly different from the previously measured samples, even though the average accuracy dropped to 57%. At 25% and 50 ms, the generated results fell below 50% accuracy. Statistical analysis showed that this was significantly lower compared to all previously measured samples except for 25% stretch and 50 ms shift (S13A Fig). When comparing the results to those generated from uncropped data augmented with 1% stretch and 5 ms shift, the results showed significantly lower accuracies compared to the previously measured cropped data (S13 Fig). This demonstrated the importance of cropping the MSC data for training the ANN. This also demonstrated that the media peak did not hold viable information for the MSC classification, but on the contrary negatively influenced the performance.

Based on these initial assessments, the performance of the trained ANN was examined in more detail. For this purpose, ten independent runs were performed, whose cumulative training loss showed good convergence already after four epochs (Fig 6C). Similar observations were made for the test accuracy, where the plateau was reached after only two to four epochs (Fig 6D). The confusion matrix for the ten test runs (Fig 6E) also proved that most of the samples were correctly classified.

The only drawback of the ANN training based on the MSC data was the relatively high standard deviation of the test accuracy (Fig 6D; shaded area). This varied between 100% and 70%, indicating some instability between samples. This could be due to the limited number of samples initially measured. With an average test accuracy of 85%, it still provided significantly better results than MSC classification using SVM (S18 Fig), which had an average accuracy of 62.67%.

Based on the MSC ANN, a similar network architecture was used to classify the data from the cell line samples. The network was also based on the ANN architecture and was optimized to achieve the highest possible accuracy. The network consisted of four convolutional layers separated by normalization and maxpooling layers. The convolutional layers were followed by four dense layers. Relu was used as the activation function and Adamax as the optimization function. Additionally, drop-out layers with a drop-out rate of 25% were used to reduce the rate of overfitting. As with the MSC classification, the architecture ended with a softmax function (S15 Fig).

**Fig 7:**
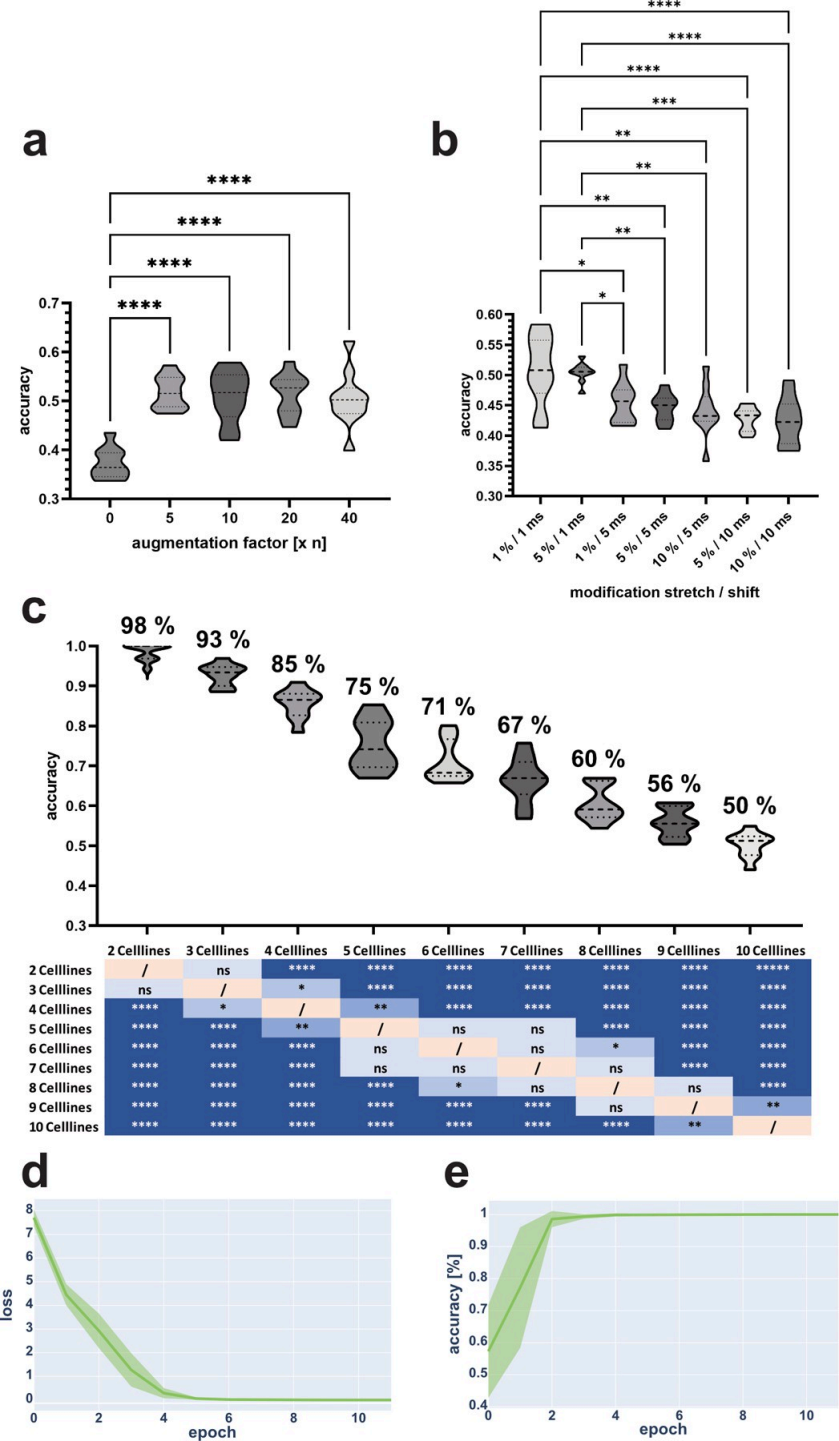
Augmentation and ANN performance for cell line classification. Different augmentation parameters were tested to evaluate their individual performance (a). As already described for the MSC classification (Fig 6), non-augmented data (augmentation factor = 0) provided significantly lower results compared to all augmentation values. There were no significant differences between the compared augmentation factors (5,10,20,40). As the amount of data directly correlated with the need time for training the ANN, it was decided to use an augmentation factor of 5 for subsequent ANN trainings. The comparison of different augmentation parameters revealed significantly lower accuracies when the corresponding data were shifted by more than 1 ms (b). According to the observed data, stretching the selected signal peak did not significantly affect the outcome of ANN training. Therefore, 1% strain and 1 ms shift were chosen as the final augmentation parameters. When used to train an appropriate ANN, accuracy correlated directly with the number of cell lines in the dataset (c). Performance was evaluated based on the cell line combinations used for training. When two cell lines (CHO, K562) were included in the training, cumulative loss converged after five to six epochs (d), while test accuracy (e) reached the plateau of 98% after two to three epochs of training. All depicted values based on n_t_ = 10; *: P ≤ 0.05 / **: P ≤ 0.01 / ***: P ≤ 0.001 / ****: P ≤ 0.0001.

The augmentation pipeline was optimized by iterations of different parameters. The optimization of the augmentation factor (Fig 7A) showed a similar behavior as before for MSC augmentation. Each augmentation value tested (5,10,20,40) showed significantly better performance compared to no augmentation. There were no detectable significant differences between the tested augmentation values. As before with the MSCs, the augmentation factor was set to five as this resulted in better performance while also reducing the time required to train the ANN. This observation was consistent across multiple cell combinations with increasing numbers of cell lines (S17B Fig).

The same augmentation parameters for shift and stretch that were tested for the MSC augmentation were also tested on the cell line data. Unlike the MSCs, the resulting accuracies for the cell lines showed significant differences (Fig 7B). The data shifted by 1 ms had significantly higher accuracy than the data based on a shift of 5 ms and 10 ms. The analysis also showed that the stretch of the signal peaks did not contribute to the overall result of ANN training, which means that the key factor was the position of the signal peak.Since they did not show significant differences, 1% stretch and 1 ms shift were chosen as the final augmentation parameters for optimal ANN performance.

For the optimal cell composition analysis, all possible combinations for the provided cells were tested and the average accuracy of ten independent runs with independent tensile test split assignments was evaluated. The best combinations (CHO, K562, HEK293T, HeLa, THP1, A549, Vero, MDA231, C2C12—in that order) were compared (Fig 7C). Similar to the previous observations for the MSC based training, the number of cell lines tested, directly correlated with the average accuracy based on the test data. In 70% of the cases, the addition of one additional cell line resulted in no significant change, while the addition of two additional cell lines resulted in a significant decrease in 80% of the cases examined (see table below Fig 7C).

The training loss and validation accuracy for training with two (Fig 7E; CHO and K562) and more cell lines (S16 Fig) showed a plateau for the validation accuracy after four to six epochs and a good convergence of training loss (Fig 7D) after four to ten epochs, which indicates that the ANN was successfully trained. Unlike MSC training, the standard deviation was much smaller, which can be explained by the increased number of training data leading to a more stable training performance.

As also the training length, given in number of training epochs, plays a vital role in the resulting accuracy of an ANN, two different training lengths were investigated. When comparing these two training lengths (12 and 25 epochs), there were no significant differences (S17A Fig). Since this saves 50% of the time needed for training, it was decided to use the twelve epochs as the final value.

Similar to the MSC ANN analysis, it was also investigated how the cropping of the data to the cell peak (T_1_: 0–3.0079 s / T_2_: 0–0.4062 s), influences the outcome of the training. Therefore, the previously used cell combinations were used to assess the network’s accuracy. The cropped results did not show any significant differences when compared to the previously used, uncropped data (S18A Fig).

Finally, the results of the SVM training for the different cell combinations were compared with the results of ANN training (S18B Fig). The only comparison that showed significant differences was for MSC classification. Here, the ANN resulted in better classification accuracy. Otherwise, none of the cell combinations examined provided conclusive evidence that either the SVM or the ANN resulted in higher classification accuracy.

## Discussion

Correct identification of cells and tissues is an essential prerequisite for the application of cell-based treatments in patients. Especially for stem cell-based therapeutics, it is of utmost importance to prove the identity and correct maturation of the cells. In most cases, these identifications are based on the assessment by trained personnel, introducing the possibility of human error risking patient safety.

To this end, this study used a mobile low-field MR scanner in combination with a low-cost automation solution to generate a large dataset of cell-based 2D T_1_ / T_2_ MR relaxometry measurements. By correctly classifying several different cell lines using support vector machines and artificial neural network (ANN), this approach was successfully demonstrated to be a viable method for non-destructive cell identification. The classification algorithms were trained on ten different cell lines with an accuracy of up to 98% (depending on the cell composition of the dataset) based on up to 362 individually measured samples magnified by an augmentation factor of five. Successful monitoring of cellular development was demonstrated with an accuracy of 85% using adipogenically differentiated MSCs as an example. Compared to cell line classification, MSC classification yielded higher variation in results, likely due to the smaller initial dataset.

The advantage of 2D MR relaxometry over other cell characterization methods, such as single cell RNA sequencing or Raman spectroscopy, is the ability to measure samples in a sterile and non-destructive manner. This facilitates the use of the presented method as an in-process control to monitor the development of cell-based therapeutic products. In the future, current limitations in sensitivity and maximum culture duration may be overcome by using microcoils for signal high-sensitivity signal detection and a (PDMS-based) bioreactor for continuous media supply for long-term culture of the appropriate cells and tissues [38]. In particular, cell viability must be interpreted in the context of the detection limit. The minimum detected average viability was 85%. With a cell count of 5e6 cells, this means that 7.5e5 cells were affected by the culture conditions. Based on the minimum detection limit of approximately 1e6 cells (S3 and S4C Figs), this would mean that the dead/impacted cells may not be detectable. Nevertheless, with an extended culture duration, it will be possible to continuously monitor cellular development and train ANN models to predict the exact differentiation stage of stem cells. It is important to note that adapting the presented method to a different problem, e.g., different cell types or cell numbers, requires the creation of an appropriate dataset a long a new training and optimization of the respective AI algorithm.

This study was not intended to provide a pre-trained model that can be used without any further investigation. Future studies will need to expand the applicability of the presented system and explore the degree of generalizability using more diverse cell lines, other primary cells, and cellular developmental processes. These studies should also take into consideration, that, depending on the respective question, different AI approaches like SVM and ANN might yield different data, like discussed for the classification of MSCs in this publication.

Furthermore, the classification and quantification of cell mixtures and tissues consisting of more than one cell type cannot be based on this proof of principle study alone. The neural network result cannot be interpreted as a quantifiable value for each class. Therefore, identification of multiple cell types within a mixture requires training of an appropriate ANN based on each required data composition. For these applications, additional measurements as well as optimization of the appropriate AI algorithm will be required.

With T_1_ / T_2_ relaxometry it is not possible to assign the measured signal to specific sample properties e.g., density, elasticity or otherwise. The interpretation of the signal relies on the comparison between the measured samples, or in this case, cell types. Nevertheless, respective MR sequences can extract specific tissue properties from the MR sample e.g., the apparent diffusion coefficient (ADC). Due to its ability to measure the membrane diffusion of the cells [39–42], the ADC could provide further details for characterizing cellular differentiation. Due to limitations in time and resources, no ADC or other measurements were performed in the context of this study.

The sample preparation step could be overcome by transferring the method to a unilateral MR system with which entire cell culture flasks could be measured. This would increase the current limited sample size and open the presented technique to more versatile application in a preclinical and clinical setting. With these more diverse data, the presented method will make a valuable contribution to preclinical research and clinical diagnostics.

## Conclusion

The aim of this study was to use 2D T_1_ / T_2_ MR relaxometry as a noninvasive, whole-mount, label-free, sterile, and unbiased method for classifying cells and cellular development. A workflow was successfully implemented to acquire a large dataset of 2D MR relaxometry data. It was possible to distinguish between adipogenic differentiated and undifferentiated mesenchymal stromal cells using the presented system, while the ANN yielded more reliable results compared to the investigated SVM. This study provides evidence that 2D MR relaxometry, performed on a low field benchtop MR scanner in combination with a custom data augmentation algorithm and an appropriate AI algorithm (SVM and ANN) can be used to distinguish between multiple different cell lines with an accuracy of up to 98%. This suggests that the presented method is suitable for use in a routine preclinical screening workflow, e.g., to evaluate the proper differentiation of a custom bioengineered tissue graft and therefore contributing to patient safety by reducing the human error.

## Material and methods

### Ethics statement

All primary cells were used in accordance with the ethical approval of the Ethics Committee of the Julius-Maximilians-University of Würzburg (Votum 280/18 and 182/10). As the cells were used under anonymized aliases, no written or verbal consent was needed from the respective patients.

### Cell culture

All measurements were performed using the cell lines CHO-K1 (ATCC CCL-61), MDA-MB-231 (ATCC HTB-26), K562 (ATCC CCL-243), THP-1 (DSMZ ACC 16), C2C12 (ATCC CRL-1772), L929 (ATCC CCL-1), A-549 (DMSZ ACC 107), Vero (ATCC CCL-81), HeLa (ATCC CCL-2), HEK293T (ATCC CRL-3216), and bone marrow-derived human mesenchymal stromal cells (MSCs). CHO-K1 and MDA-MB-231 will be referred to hereafter only as CHO and MDA231, respectively. All cell lines were kindly provided by the Department of Tissue Engineering and Regenerative Medicine of the University Hospital Würzburg.

All adherent cells (CHO, MDA231, C2C12, L929, A-549, Vero, HeLa, HEK293T, and MSC) were cultured in standard T150 cell culture flasks (TPP AG, CH), whereas suspension cells K562 and THP-1 were grown in untreated T75 flasks (Thermo Fisher Scientific Inc., US). The adherent cells were cultured to 70–80% confluence and then passaged with trypsin-EDTA solution (Merck KGaA, DE) diluted in Dulbecco’s phosphate-buffered saline modified without calcium and magnesium chloride (DPBS-; Merck KGaA, DE). Cells were cultured until passage 15. Suspension cells (K562, THP-1) were cultured until they reached a density of 1e6 cells per milliliter. They were then repopulated with 2e5 cells per milliliter. C2C12, L929, HEK293T, and HeLa cells were cultured in DMEM containing high glucose (4.5 g/l) and GlutaMAX (Thermo Fisher Scientific Inc., USA) supplemented with 10% FBS (PAN-Biotech GmbH, Germany) and 1% penicillin-streptomycin (Merck KGaA, DE). Vero cells were cultured in the same medium supplemented with 1% sodium pyruvate (Thermo Fisher Scientific Inc., USA). MDA231, K562, and A-549 were cultured in RPMI 1640 medium supplemented with GlutaMAX (Thermo Fisher Scientific Inc., US) supplemented with 1% penicillin-streptomycin (Merck, KGaA, DE) and 10% FBS (PAN-Biotech GmbH, DE). CHO cells were cultured in DMEM/F-12 with GlutaMAX (Thermo Fisher Scientific Inc., US) supplemented with 1% penicillin-streptomycin and 10% FBS. THP-1 cells were cultured in RPMI-1640 medium supplemented with 1% penicillin-streptomycin and 10% FBS (Thermo Fisher Scientific Inc., US). The culture medium was replaced every 2–3 days. Cells were maintained at a relative humidity of 95% at 37°C and 5% CHO2.

### Sample preparation

Cells were cultured as described above and detached with trypsin-EDTA. They were counted using an improved Neubauer counting chamber, and the appropriate number of cells, usually 5e6, was transferred to a 200 μl plastic tube (Biozym Scientific GmbH, Germany). Unless otherwise indicated, the sample volume was always maintained at 50 μl. All attachments of the plastic tube were cut off. It was placed in a glass tube (SCHOTT AG, Germany), which was sealed with a silicone stopper (Deutsch & Neumann GmbH, Germany). Finally, the cells were pelleted at 300 xg for 3 minutes.

### Culture, differentiation, and MR measurement of human mesenchymal stromal cells (hMSC)

The bone marrow mesenchymal stromal cells (MSCs) used were isolated from patients undergoing surgical femoral head replacement. The cells were used in accordance with the ethical approval of the Ethics Committee of the Julius-Maximilians-University of Würzburg (Votum 280/18 and 182/10). As the cells were used under anonymized aliases, no written or verbal consent was needed from the respective patients.

Cells were cultured in a standard T150 cell culture flask (TPP AG, CH) and maintained under standard cell culture conditions until they reached approximately 70–80% confluence. DMEM/F12 (1:1) basal medium (Thermo Fisher Scientific Inc., US) supplemented with 10% FBS (Bio&Sell GmbH, DE) and 1% penicillin-streptomycin was used for expansion. Cells were passaged according to the same protocol described above for cell culture of the cell lines used. MSCs were generally used until the fourth passage.

For differentiation, cells were cultured until they reached near 100% confluence. They were separated and the number of viable cells was determined using trypan blue (Merck KGaA, DE) and a Neubauer counting chamber (BRAND GmbH + Co KG, DE). The volume containing 5e6 cells was centrifuged at 300 xg for 3 minutes and the supernatant was then completely aspirated. The cell pellet was suspended in 50 μl of fresh medium and transferred to a plastic tube (BioZym Biotech Trading GmbH, AT). The attachments of the plastic tube were cut off. The plastic tube was placed in a glass tube (SCHOTT AG, Germany) which was sealed with a silicone stopper (Deutsch & Neumann GmbH, Germany). The cells were then pelleted at 300 xg for 3 minutes. As a reference, these undifferentiated cells were measured with the MR scanner. After measurement, the 5e6 cells were transferred to a standard T75 cell culture flask (TPP AG, CH) and allowed to adhere overnight in the incubator.

For differentiation, the medium was replaced the next day with the appropriate adipose differentiation medium (ADM) consisting of DMEM with high glucose content (4. 5 g/l) with GlutaMAX supplemented with 10% FBS (Bio&Sell GmbH, DE), 1 μM dexamethasone (Merck KGaA, DE), 1 μg/ml insulin (Merck KGaA, DE), 100 μM indomethacin (Merck KGaA, DE), 500 μM IBMX (Applichem, US), 1% D-glucose (Merck KGaA, DE) and 0.1% lipid mix (Merck KGaA, DE). The medium was replaced every two to three days. After 21 days the cells were washed using PBS^-^ and then detached using Trypsin/EDTA. The samples for measurement were prepared as described above. The adipose differentiation followed the same protocol as previously described in Malkmus et al. [43].

### Validation of cellular differentiation

To validate the differentiation process, undifferentiated MSCs were seeded into a 6-well plate (TPP, CH). They were cultured with the previously described expansion medium. When cells reached confluence, they were rinsed with PBS, and the medium was replaced with 2 ml of MSC differentiation medium described previously. The cells were cultured for 21 days under standard cell culture conditions. After differentiation, the cells were washed twice with PBS-. They were fixed for 10 min at room temperature with ROTI Histofix 4% (Carl Roth GmbH + CO. KG, DE). Cells were rinsed with deionized water and then incubated with 60% 2-propanol (Carl Roth GmbH + Co. KG) for five min. Lipid droplets were stained with the prepared Oil Red O staining solution (stock concentration: 0.5 g Oil Red O [Merck KGaA, DE] to 100 ml 2-propanol; staining solution: 60% stock solution + 40% deionized water) for 10 minutes at room temperature, followed by two rinsing steps, first with 60% 2-propanol and then with deionized water.

### Operating the automatization platform

The developed automatization platform was based on the commercially available Braccio Robot (Arduino, IT). The robot was powered by small server motors. The gripper was customized using Autodesk Inventor (Autodesk, US) and 3D printed. The Braccio robot was connected to an Arduino Mega microcontroller (Arduino, IT) running a custom script. The Arduino was connected to a laptop via USB. On the laptop the user was able to control the robot using a custom designed Matlab interface. From the Matlab interface, the user could define preset robot positions and add procedures which the robot should follow to process the samples.

All relevant positions were saved within the Matlab interface. The process workflow was designed, so that the user would input a glass sample tube into the custom build sample box. Within the sample box a microswitch was connected to the microcontroller. Therefore, the user interface presented a pop-up window to the user to input all viable information like sample name, project name, measurement conditions and MR sequence. After the user confirmed the input, they could add up to eleven additional samples, resulting in a total capacity of twelve samples within the system. As soon as the process was started, the robot transferred up to four samples to the included heating block, preheating them to 37°C. As soon as the preheating was finished the samples were transferred to the MR scanner where they were measured. As soon as the measurement was finished, the respective samples was transferred back to the sample box and the empty space in the heating block was filled with additional samples, if necessary. This was repeated until all samples had been processed.

All components were mounted on a plastic PVC plate, so that the positions, relative to each other stayed consistent. If the positions of the components were changed, the respective coordinates for the robot had to be adjusted accordingly.

### Viability assessment of cells

Cells were cultured and processed as described above. Once the 50 μl cell suspension was transferred to the plastic tube and then to the glass tube, they were placed in a preheated heating block and kept at 37° for 40 minutes. They were then brought to room temperature (RT) for 4 hours and 20 minutes. The total time of 5 h represents the maximum time a sample would spend in the measurement process. For the assessment at RT, the cells were kept on the working bench for the entire duration of the test, without transferring them to a heating device. To investigate the influence of the incubator on the viability, the prepared cell sample was instantly transferred to a standard cell culture incubator after preparation. The sample was kept there for the entire duration of the test.

At the end of the 5 h, the cells were transferred to a fresh 1.5 ml centrifugation tube along with 950 μl of fresh cell culture medium. For viability measurement, a 1:10 dilution in the appropriate cell culture medium was transferred to a fresh 1.5 ml centrifugation tube with a total volume of 1 ml. Using this suspension, cell viability was measured using the NucleoCounter NC-200 and the corresponding Via1 Cassettes (ChemoMetec, DK). Each of the 10 cell lines used and the MSCs were measured independently three times.

As a negative control, cells were maintained at 90°C and 300 rpm for 30 minutes. As a positive control, the cells were processed as described above, but then immediately measured for viability.

### Preparation of agarose dotagraf cell phantoms (ADCP)

For the preparation of agarose cell phantoms, RPMI1640 base medium (Thermo Fisher Scientific Inc., US) was used supplemented with 1/2000 Dotagraf (Jenapharm, DE), resulting in a final concentration of 0.25 μmol/ml to achieve the desired T_2_ values. For T_1_ adjustment, 0.6% agarose (Genaoxx bioscience GmbH, DE) was added. The mixture was heated in a microwave at 700 W for several seconds until the agarose was completely dissolved. The ADCP was prepared in a larger stock of 20 ml and stored at 4°C. When needed, it was reheated in the microwave and the appropriate volume was taken to prepare the corresponding samples.

### MR Sequence for T_1_ / T_2_ relaxometry

All measurements were performed on an adapted version of the table-top MR system "magspec" together with the "drive-l" console (PureDevices GmbH, DE). The 90° and 180° pulses had a rectangular shape and identical amplitude but were of different lengths. The sequence (S1A Fig) consisted of an inversion recovery sequence (IRS), starting with a rectangular 180° pulse at t_0_, followed by a rectangular 90° pulse after a certain inversion time (TI). TI was logarithmically increased 32 times, starting at 5 ms and ending at 15 s. After each inversion, a multispinecho (CPMG sequence) of 5000 rectangular 180° pulses were performed to thoroughly evaluate the T_2_ decrease. Each pulse was separated by an inter echo time (TE) of 3 ms, starting at TE/2 after the 90° excitation pulse of the IRS. Each echo was acquired with a bandwidth of 200 kHz and 40 data points, giving a data size of 32 x 5000 x 40. Because the data are based on different TIs, they represent information in the time domain. Before each measurement, the Larmor frequency for the sample was automatically determined. Shim values were determined once and then used again for each sample.

### Postprocessing of measured data

The 5000 datapoints of the CPMG sequence were re-gridded to a logarithmic scale and for reducing the complexity of the data, the number of T_2_ datapoints was mapped to match 256. The data were then normalized before a basic noise reduction was carried out, using a principal component analysis (PCA). The data were inverted in T_1_ before they were fitted using a two-dimensional Laplace transformation, based on a 300 by 300 matrix. Because of the Laplace transformation, the data no longer represent information in the time domain but in the frequency domain.

### Data augmentation

Augmentation was performed using a self-designed Matlab class [44]. It mapped the data on a linear scale before it was modified. It then isolated the peaks and selected them by size and intensity. One operator of the class randomly shifted the selected peak within a defined standard deviation specified in seconds. The other operator stretched the peak by a random percentage (based on the target standard deviation) of the original peak. Both operators could be set individually in T_1_ and T_2_ dimensions but were usually kept identical for both possible dimensions. Finally, the data were again mapped to a logarithmic scale. The class was run n-times to increase the amount of available data by a specified augmentation factor. For every iteration, the augmentation algorithm randomly selected new augmentation parameters within the given range to ensure a diversity of newly generated data.

### Training and assessment of the Support Vector machine (SVM)

For the training of the SVM, the weighted centroids of the given data were calculated. Then the targeted cells could be extracted from the over-all data pool, in order to only investigate the performance on a defined combination of cells. The data then was split into training and testing data by a factor of 0.5. Using the *sklearn*.*svm* class from python, a SVM using a polynomial kernel, a degree of 2 and a coefficient of 0 was fitted to the given training data. The trained SVM model was then evaluated based on the previous testing data, yielding the final accuracy.

The training was carried out 300 independent times, each time with a new train-test-split. The resulting testing accuracies were analyzed using GraphPad Prism.

### Artificial neuronal networks for classification

Each machine learning and deep learning approach was performed using Python 3 [45]. The following packages were used to set up, train test and document the ANN: PyTorch [46], Tensorboard [47,48], NumPy [49], Matplotlib [50], Scikit-learn [51,52], Seaborn [47], Netron [53], and Pandas [54,55].

The data was exported from Matlab as *.png files and imported into Python. There it could be selected, which cell types to include into the later analysis. The data were then split into train (75%), validation (20% of training data) and testing data (25% of initial data). The custom train-test-split function selected the cells with an equal distribution, to ensure that every cell line / type was part of every data group. Otherwise, due to the unbalanced dataset, it might be possible, that the test dataset would hold data that was not part of the training dataset. This method was called ‘Celltype aware Train-Test-Split’.

The architecture of the model was based on the VGG architecture (37). For the classification of the MSCs it consisted of seven convolutional layers followed by four dense layers. The convolutional layers were separated maxpooling layers after every other layer, starting after the first one (s. S14 Fig for more details). Relu was chosen for both layer types (convolutional and dense) as the activation function. Adamax was chosen as the optimizer function.

For the classification of the cell lines, the architecture used four convolutional layers separated by maxpooling layers after every layer. Like for the MSC classification four dense layers followed the convolutional layers (s. S15 Fig for more details). Differing from the MSC classification, dropout layers with a dropout rate of 25% were used to minimize the overfitting. Every ANN training ended with a softmax function.

Every ANN training was carried out ten independent times with a new train-validation-test split for every iteration. The key performance indicators (batch loss, comulative loss, training accuracy, validation accuracy and confusion matrix) were exported using tensorboard. The respective tensorboard files are attached to this publication. The resulting accuracies were documented and analyzed using GraphPad Prism.

The final network architecture was exported as an ONNX file and was visualized using Netron.

## Supporting information

S1 Fig The MR sequence produced fully relaxed T1 and T2 signals for a two-dimensional measurement. A combined T_1_ and T_2_ sequence (a) was used for two-dimensional data acquisition. It consisted of an inversion recovery sequence with different inversion times for T_1_ estimation, followed by a CPMG sequence with 5000 rectangular 180° pulses for T_2_ decay estimation. The sequence was repeated 32 times, with inversion times (TI) varying from 5 ms to 15 s for each repetition to fully capture T_1_ remodeling. The resulting data could be plotted in a three-dimensional space (b), where one dimension reflected the T_1_ remodeling, the other the T_2_ estimate, and the third the corresponding amplitude. Values for all measurement parameters were chosen to produce a fully relaxed T_1_ (c1) and T_2_ (c2) signal. The data were post-processed using an inverse two-dimensional Laplace transform to extract the corresponding T_1_ (d1) and T_2_ (d2) spectra. All pulses used were rectangular RF pulses with identical amplitude but different length. The graphical depiction of the sequence in (a) is only a scheme. The RF pulses are depicted as black rectangles and the respective echo as a curve. The three dots at the end represent the remaining 4997 180° pulses.
(TIFF)

S2 Fig Spectral differences between samples containing media only and samples containing cells. When measuring samples containing only the appropriate medium, the signal showed a single peak (**a**). As soon as cells were added to the sample, peaks with shorter T_1_ and T_2_ times became detectable (**b**), while the previously described peak with higher relaxation times remained consistently in place. Because this was reproducible in each measurement, it was assumed that the cell-related information of the measurement was contained in the peaks with the shorter T_1_ and T_2_ times. Based on this assessment, these peaks were referred to as cell peaks, while the larger peak at higher relaxation times was referred to as the media peak. Each plot of the spectrum was trimmed to the range between 5e-1 s to 5 s in T_1_ and to 1e-1 s to 4 s for T_2_, as this was the range of interest.
(TIFF)

S3 Fig Using HEK293T cells to define minimal detectable cell number. HEK293T cells were used to investigate the minimum number of cells that can be detected without signal change. For this purpose, four independent, biological replicates were prepared and measured for each cell number in question (1e6, 3e6, 6e6, 8e6, 1e7). The signal was stable down to 6e6 cells. At 3e6 and 1e6, the T_1_ signal began to drift toward longer relaxation times. The T_2_ signal remained stable until 3e6. At 1e6, the signal from the cell peak began to fade in intensity. On this basis, the minimum detectable cell number was set at > 3e6 cells. The described fading also indicates, that the relative composition of the sample in regard to cell to media ratio, directly correlates with the respective peak intensity.
(TIFF)

S4 Fig Statistical analysis of centroids for stability measurements. Weighted centroids were calculated for the data shown in Fig 1. The calculated T_1_ and T_2_ values were compared to statistically test the previously postulated hypothesis based on the visual impression of the plotted data. Statistical analysis of ADCP samples measured over a 14-day period did not reveal statistically significant differences in either T_1_ (a1) or T_2_ values. The same was true for the comparison of the studied sample volume. No significant differences were found for both ADCP volumes studied (T_1_—b1; T_2_—b2). To also validate the cell number used, the weighted centroids for the respective samples from S3 Fig were calculated and analyzed. The T_1_ values for 1e6 cells yielded significantly higher values compared with any other cell number (c1). The respective T_2_ values did not show significant differences (c2). This analysis confirmed the first visual impression of the data.
(TIFF)

S5 Fig Additional details on the assessment of viability after different treatments of parts of the studied cells. During the automatic evaluation of cell viability, the diameter of the cells was also measured. The statistics showed several significant differences between the different diameters. CHO cells had the largest diameter, while Vero cells had the smallest. Since cell diameter directly correlates with the size of the cell pellet produced for the 5e6 cells, this also provides information about the ratio of cells to media. However, since the CHO cells have significantly higher viability compared to the Vero cells (Fig 2), cell diameter does not appear to have a decisive effect on cell viability in the culture method presented. The individual viability measurements (b) provide detailed insights into the scatter of the data for each cell type. *: P ≤ 0.05 / **: P ≤ 0.01 / ***: P ≤ 0.001 / ****: P ≤ 0.0001
(TIFF)

S6 Fig Separate scatter plots for each cell type studied showing clustering of weighted centroids for the media peaks of the measured MR spectra. When calculating the weighted centroids for each cell peak of the measured data and creating separate scatter plots for each measured cell line, the local orientation and distribution became apparent. Most cell lines showed an elongated distribution along the T_2_ axis. Some, such as C2C12 or MDA231, were more locally aggregated.
(TIFF)

S7 Fig Separate comparison of the two-dimensional spectra for the cells used. Plotting all spectra recorded for one cell type on a contour plot reveals the two groups of peaks described previously. The media peak was ≈ 2–3 s/3-4 s T1/T2, whereas the position of the cell peaks varied a little for each cell type. Some of the cells used showed a more densely packed group of cell peaks such as the CHO, C2C12, MSC, and A549 cells, whereas others were distributed over a wider range of T_1_ and T_2_ values such as HEK293T, HeLa, K562, L929, MDA231, THP1, and Vero cells. Each plot consisted of all measured spectra for the respective cell type as superimposed contour plots.
(TIFF)

S8 Fig Morphological differences between cultured cells. All eleven cells used in this study were classically grown in a two-dimensional monoculture. K562 and THP1 cells were suspension cells and therefore did not adhere to the surface of the culture flask. All other cells adhered to the surface. Some cells such as A549, C2C12, CHO, Vero, and HEK293T had a more compact appearance, while HeLa, L929, MDA231, and MSCs had more elongated phenotypes. The scale bar corresponds to 100 μm.
(TIFF)

S9 Fig Plot of SVM decision boundaries and corresponding training and test data. For each setup of SVM, the decision boundaries used by the system were plotted along with the corresponding training and test data. The decision boundaries show that the systems struggle to make appropriate decisions to partition the training data into the appropriate number of classes defined by the number of input cells. Plotting the training and test data provided evidence that the SVM algorithm was indeed trained on the training data only and that the test data indeed represented previously unseen values. A train-test split ratio of 0.5 was chosen for the SVM. For each cell combination, the system was run 300 times. The visualization shows only an example representation of the decision boundaries together with an instance of the training and test data.
(TIFF)

S10 Fig Plot of SVM decision boundaries and corresponding training and test data. For each setup of SVM, the decision boundaries used by the system were plotted along with the corresponding training and test data. The decision boundaries show that the systems struggle to make appropriate decisions to partition the training data into the appropriate number of classes defined by the number of input cells. Plotting the training and test data provided evidence that the SVM algorithm was indeed trained on the training data only and that the test data indeed represented previously unseen values. A train-test split ratio of 0.5 was chosen for the SVM. For each cell combination, the system was run 300 times. The visualization shows only an example representation of the decision boundaries together with an instance of the training and test data.
(TIFF)

S11 Fig Showing effects of data augmentation on MSC spectra. Each MSC measurement was augmented by a factor of 30. When the augmented spectra are plotted on top of each other, the effects produced become visible. Therefore, each area separated by white lines consist of 30 individual spectra. Due to the logarithmic scaling, the augmentation had a stronger effect on the depiction of the signal peaks at lower T_1_ and T_2_ times than on those at higher relaxation times. The spectra of the undifferentiated MSCs (a) showed only two peaks in T_1_ and T_2_, whereas the spectra of the differentiated MSCs (b) mostly showed three peaks. The lines in the diagrams represent the groups of 30 belonging to an original measurement.
(TIFF)

S12 Fig Effects of the augmentation algorithm on cell-based data. The effects of the position shift aspect of the data augmentation were more pronounced for the cell peaks due to their shorter relaxation times. This was similar for A549 (a; n_b_ = 17), C2C12 (b; n_b_ = 30), CHO (c; n_b_ = 61), HEK293T (d; n_b_ = 21), HeLa (e; n_b_ = 24), K562 (f; n_b_ = 57), L929 (g; n_b_ = 30), MDA231 (h; n_b_ = 57), THP1 (i; n_b_ = 26), and Vero (j; n_b_ = 16) cells. The plots shown also demonstrate the stability of the media peak across all ten cell lines measured. Shown are plots with an augmentation factor of 10. The lines drawn delineate each cluster of augmented data derived from the first measurement. The number of clusters in the plots is different for each cell line due to the different number of measurements.
(TIFF)

S13 Fig The comparison of exceptionally high augmentation values for MSC spectral data proves to be significant. In addition to the augmentation values of 1% - 10% stretch and 1 ms—10 ms shift already shown, other high augmentation values were tested. 25% / 50 ms showed no significant differences from other augmentation values, while 25% / 50 ms was significantly different from any other reported augmentation value (a). Because 1% / 5 ms had the highest mean value (85%), it was compared with performance for the same data set but without the media peak cropping. The statistics showed that except for the augmentation for 25% / 50 ms, the performance without cropping to 0–3.0079 s for T_1_ and 0–0.4062 s for T_2_ showed significant differences from all other augmentation values. This suggests that the media peak reduces the accuracy of the ANN when trained on MSC data. *: P ≤ 0.05 / **: P ≤ 0.01 / ***: P ≤ 0.001 / ****: P ≤ 0.0001
(TIFF)

S14 Fig Architecture of the ANN for the classification of MSCs. The architecture was based on a modified VGG neural network whose parameters were optimized to achieve the highest accuracy on the available data. The resulting structure consisted of seven convolutional layers followed by four dense layers. Normalization and maxpooling layers were inserted between the convolutional layers. Relu was chosen as the activation function and Adamax as the optimizer. The architecture ended with a softmax function. Further details can be found in the schematic above. The respective ONNX file from which this schematic was generated has been attached to this publication.
(TIFF)

S15 Fig Architecture of the ANN for the classification of Cell lines. The architecture was based on a modified VGG neural network whose parameters were optimized to achieve the highest accuracy on the available data. It consisted of four convolutional layers separated by normalization and maxpooling layers. The convolutional layers were followed by four dense layers. Relu was used as the activation function and Adamax as an optimizer. To reduce possible overfitting effects, a dropout of 0.25 was applied to the dense layers. The architecture ended with a softmax function. Further details can be found in the schematic above. The respective ONNX file from which this schematic was generated has been attached to this publication.
(TIFF)

S16 Fig Exemplary depiction of confusion matrix, validation accuracy and training loss for ANN training. Confusion matrices for the final ANN test (values averaged over ten replicates) demonstrate that most cell lines were correctly classified. These observations correlate with the low variance in the power plots. Accuracy reached its plateau after only four to six epochs. Training loss converged as expected with low variance (shaded area) between the ten independent replicates. All other data can be exported from the attached tensorboard data files.
(TIFF)

S17 Fig Optimization of the number of epochs and the augmentation factors to be achieved. When comparing two different epochs with augmentation factors of 0, 5, 10, 20, and 40, the statistics showed that there were no significant differences between a training duration of 12 and 25 epochs (a). When further comparing different augmentation factors for different cell compositions (b), the statistics showed the previously described pattern that any augmentation factor greater than zero was significantly better than no augmentation (augmentation factor = 0). This was consistent across several different cell compositions. CC06: CHO, K562, HEK293T / CC07: CHO, K562, HEK293T, HeLa / CC18: CHO, K562, HEK293T, HeLa, THP1; *: ≤ 0.05 / **: P ≤ 0.01 / ***: P ≤ 0.001 / ****: P ≤ 0.0001
(TIFF)

S18 Fig Investigating the influence of cropping the data to the cell peak and comparing the ANN and SVM results. When examining the effect of cropped and uncropped data on ANN performance (a), the statistics showed no significant differences between the two conditions. Similar observations were made when comparing SVM performance with ANN performance (b). No significant differences were found except for the MSCs. The performance of the SVM compared with the ANN for the classification of MSC differentiation was subject to significantly higher scatter. While the ANN only showed results with high accuracies, the SVM gave highly variable and therefore unreliable results between 0 and 100%. Each comparison was performed for the combinations of cell lines that yielded the highest average accuracy. All measurements were carried out ten indipendent times. ****: P ≤ 0.0001
(TIFF)

S1 Table Cell combinations used for SVM and ANN training. The following cell combinations represent the optimal compositions for the respective number of incorporated cells for SVM and ANN training.
(TIFF)

S1 Data Respective data to recreate Fig 1 including the necessary spectral data as CSV files. (ZIP)

S2 Data (a) 3D STL Files for self-designed robotics components, that can be used to print the respective components. (b) Raw data including statistical output to follow statistical analysis. (c) Raw data including statistical output to follow statistical analysis.
(ZIP)

S3 Data (a) All measured spectral information for cell line analysis as CSV files. (b) Spectral information regarding differentiated and undifferentiated MSC data as png files. (c) Information regarding the dataset composition for cell lines and MSCs as CSV files. (d) Data for weighted centroids as T1 and T2 coordinates for cell lines and MSCs as CSV files.
(ZIP)

S4 Data Data to follow the statistical analysis including raw data as CSV files. Data that was used to train the SVM. As the train-test data were assigned randomly for every training iteration, the individual data used for generating the subfigures b–e are not separately listed, as these cannot be manually recreated but depend on the train-test assignment by the algorithm.
(ZIP)

S5 Data The calculated T_1_-T_2_ coordinates, split for every used set of augmentation parameters. The data is saved as *csv file with the first column representing the cell tag, the second column representing the T_1_ and the third column the T_2_ coordinate.
(ZIP)

S6 Data All augmented MSC data that was generated for this manuscript. The data is saved as *.png files. The subfolder entitled ‘0’ represent the undifferentiated cells while ‘1’ represent the differentiated MSCs. Every dataset also includes the original data, that were not affected by the augmentation algorithm (refer to tag ‘_Original.png’).
(ZIP)

S7 Data All augmented cell line data that was generated for this manuscript (part 1). This part includes the data augmented with 0% stretch and 0 ms shift, and 1% stretch and 1 ms shift. Every dataset also includes the original data, that were not affected by the augmentation algorithm (refer to tag ‘_Original.png’).
(ZIP)

S8 Data All augmented cell line data that was generated for this manuscript (part 2). This part includes the data augmented with 1% stretch and 5 ms shift. Every dataset also includes the original data, that were not affected by the augmentation algorithm (refer to tag ‘_Original.png’).
(ZIP)

S9 Data All augmented cell line data that was generated for this manuscript (part 3). This part includes the data augmented with 5% stretch and 1 ms shift. Every dataset also includes the original data, that were not affected by the augmentation algorithm (refer to tag ‘_Original.png’).
(ZIP)

S10 Data All augmented cell line data that was generated for this manuscript (part 4). This part includes the data augmented with 5% stretch and 5 ms shift. Every dataset also includes the original data, that were not affected by the augmentation algorithm (refer to tag ‘_Original.png’).
(ZIP)

S11 Data All augmented cell line data that was generated for this manuscript (part 5). This part includes the data augmented with 5% stretch and 10 ms shift. Every dataset also includes the original data, that were not affected by the augmentation algorithm (refer to tag ‘_Original.png’).
(ZIP)

S12 Data All augmented cell line data that was generated for this manuscript (part 6). This part includes the data augmented with 10% stretch and 5 ms shift. Every dataset also includes the original data, that were not affected by the augmentation algorithm (refer to tag ‘_Original.png’).
(ZIP)

S13 Data All augmented cell line data that was generated for this manuscript (part 7). This part includes the data augmented with 10% stretch and 10 ms shift. Every dataset also includes the original data, that were not affected by the augmentation algorithm (refer to tag ‘_Original.png’).
(ZIP)

S14 Data (a) Information needed to follow the statistical analysis including the used raw data as CSV files. (b) Information needed to follow the statistical analysis including the used raw data as CSV files. (c) Respective run data for the documented training loss for each iteration as a CSV file. (d) Respective run data for the documented validation accuracy for each iteration as a CSV file. (e) Summarized data for following the generated confusion matrix. Additionally, every generated tensorboard file was added, so that every performed training run can be recreated.
(ZIP)

S15 Data (a) Information needed to follow the statistical analysis including the used raw data as CSV files. (b) Information needed to follow the statistical analysis including the used raw data as CSV files. (c) Information needed to follow the statistical analysis including the used raw data as CSV files. (d) Respective run data for the documented validation accuracy for each iteration as a CSV file. (e) Summarized data for following the generated confusion matrix.
(ZIP)

S16 Data Every generated tensorboard file was added, so that every performed training run can be recreated (Part 1). (ZIP)

S17 Data Every generated tensorboard file was added, so that every performed training run can be recreated (Part 2). (ZIP)

S18 Data Every generated tensorboard file was added, so that every performed training run can be recreated (Part 3). (ZIP)

S19 Data Every generated tensorboard file was added, so that every performed training run can be recreated (Part 4). (ZIP)

S20 Data Every generated tensorboard file was added, so that every performed training run can be recreated (Part 5). (ZIP)

S21 Data Every generated tensorboard file was added, so that every performed training run can be recreated (Part 6). (ZIP)

S22 Data Every generated tensorboard file was added, so that every performed training run can be recreated (Part 7). (ZIP)

S23 Data Every generated tensorboard file was added, so that every performed training run can be recreated (Part 8). (ZIP)

S24 Data Every generated tensorboard file was added, so that every performed training run can be recreated (Part 9). (ZIP)

S25 Data Every generated tensorboard file was added, so that every performed training run can be recreated (Part 10). (ZIP)

S26 Data Every generated tensorboard file was added, so that every performed training run can be recreated (Part 11). (ZIP)

S27 Data Every generated tensorboard file was added, so that every performed training run can be recreated (Part 12). (ZIP)

S28 Data Every generated tensorboard file was added, so that every performed training run can be recreated (Part 13). (ZIP)

S29 Data Every generated tensorboard file was added, so that every performed training run can be recreated (Part 14). (ZIP)

S30 Data Every generated tensorboard file was added, so that every performed training run can be recreated (Part 15). (ZIP)

S31 Data Every generated tensorboard file was added, so that every performed training run can be recreated (Part 16). (ZIP)

S32 Data Every generated tensorboard file was added, so that every performed training run can be recreated (Part 17). (ZIP)

S33 Data Every generated tensorboard file was added, so that every performed training run can be recreated (Part 18). (ZIP)

S34 Data Every generated tensorboard file was added, so that every performed training run can be recreated (Part 19). (ZIP)

S35 Data Every generated tensorboard file was added, so that every performed training run can be recreated (Part 20). (ZIP)

S1 Dataset (S3) Respective spectral data as CSV files. (S4) Information needed to follow the statistical analysis including the used raw data as CSV files. (S5) Information needed to follow the statistical analysis including the used raw data as CSV files. (S6) Coordinates for the calculated weighted centroids from all measured cell line data as a CSV file. (S7) Every acquired 2D spectrum for the measured cell lines. (S9 and S10) Coordinates for the calculated weighted centroids from all measured cell line data as a CSV file. (S11) Respective spectral data as CSV files. (S12) Respective spectral data as CSV files. (S13) Information needed to follow the statistical analysis including the used raw data as CSV files. (S14) ONNX file for the ANN used to classify MSCs. (S15) ONNX file for the ANN used to classify cell lines. (S16) Respective data for recreating the depicted confusion matrices as well as the training loss and validation accuracy. (S17) Information needed to follow the statistical analysis including the used raw data as CSV files. (S18) Information needed to follow the statistical analysis including the used raw data as CSV files.
(ZIP)
