# Supplementary figures and images for: Non-destructive classification of unlabeled cells: Combining an automated benchtop magnetic resonance scanner and artificial intelligence (part 1 of 3)
Source: PLoS Comput Biol. 2023 Feb 21;19(2):e1010842. doi: 10.1371/journal.pcbi.1010842 (PMC9983908; doi:10.1371/journal.pcbi.1010842)

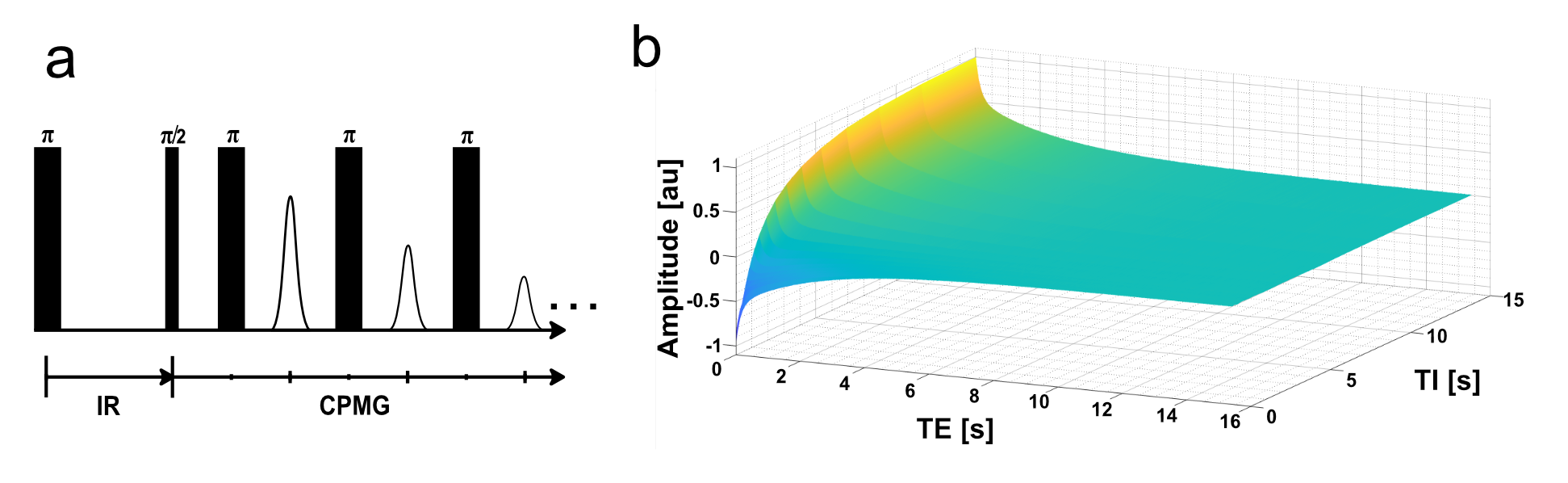

Supplement: S1 Fig — A combined T1 and T2 sequence (a) was used for two-dimensional data acquisition. It consisted of an inversion recovery sequence with different inversion times for T1 estimation, followed by a CPMG sequence with 5000 rectangular 180° pulses for T2 decay estimation. The sequence was repeated 32 times, with inversion times (TI) varying from 5 ms to 15 s for each repetition to fully capture T1 remodeling. The resulting data could be plotted in a three-dimensional space (b), where one dimension reflected the T1 remodeling, the other the T2 estimate, and the third the corresponding amplitude. Values for all measurement parameters were chosen to produce a fully relaxed T1 (c1) and T2 (c2) signal. The data were post-processed using an inverse two-dimensional Laplace transform to extract the corresponding T1 (d1) and T2 (d2) spectra. All pulses used were rectangular RF pulses with identical amplitude but different length. The graphical depiction of the sequence in (a) is only a scheme. The RF pulses are depicted as black rectangles and the respective echo as a curve. The three dots at the end represent the remaining 4997 180° pulses. (TIFF) [file pcbi.1010842.s001.tiff]

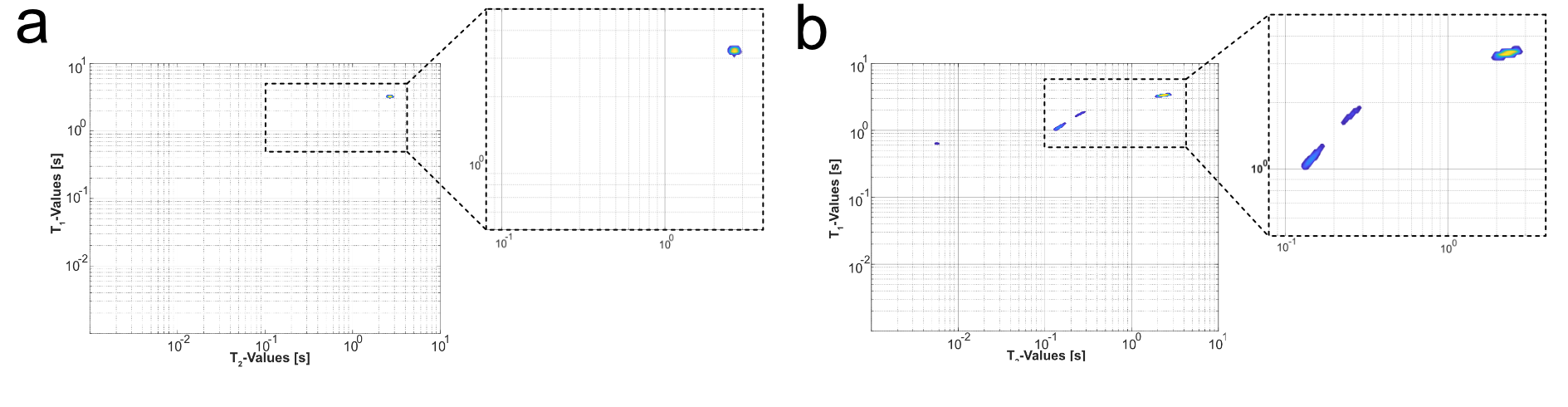

Supplement: S2 Fig — When measuring samples containing only the appropriate medium, the signal showed a single peak (a). As soon as cells were added to the sample, peaks with shorter T1 and T2 times became detectable (b), while the previously described peak with higher relaxation times remained consistently in place. Because this was reproducible in each measurement, it was assumed that the cell-related information of the measurement was contained in the peaks with the shorter T1 and T2 times. Based on this assessment, these peaks were referred to as cell peaks, while the larger peak at higher relaxation times was referred to as the media peak. Each plot of the spectrum was trimmed to the range between 5e-1 s to 5 s in T1 and to 1e-1 s to 4 s for T2, as this was the range of interest. (TIFF) [file pcbi.1010842.s002.tiff]

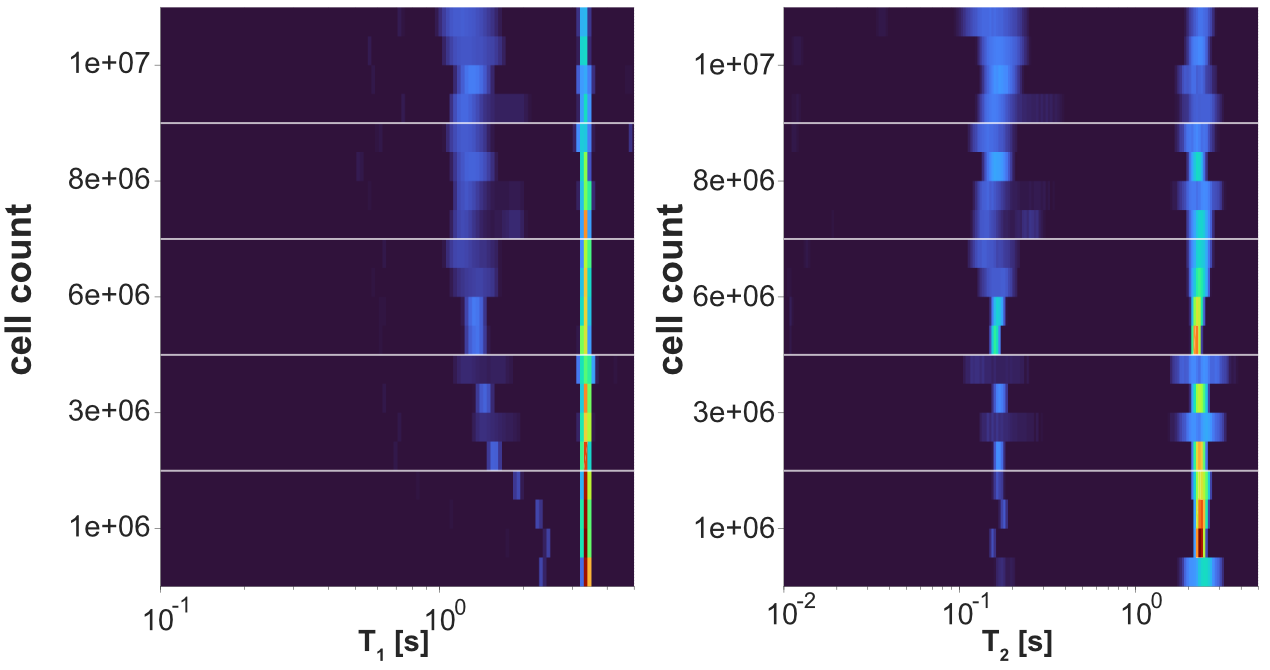

Supplement: S3 Fig — HEK293T cells were used to investigate the minimum number of cells that can be detected without signal change. For this purpose, four independent, biological replicates were prepared and measured for each cell number in question (1e6, 3e6, 6e6, 8e6, 1e7). The signal was stable down to 6e6 cells. At 3e6 and 1e6, the T1 signal began to drift toward longer relaxation times. The T2 signal remained stable until 3e6. At 1e6, the signal from the cell peak began to fade in intensity. On this basis, the minimum detectable cell number was set at > 3e6 cells. The described fading also indicates, that the relative composition of the sample in regard to cell to media ratio, directly correlates with the respective peak intensity. (TIFF) [file pcbi.1010842.s003.tiff]

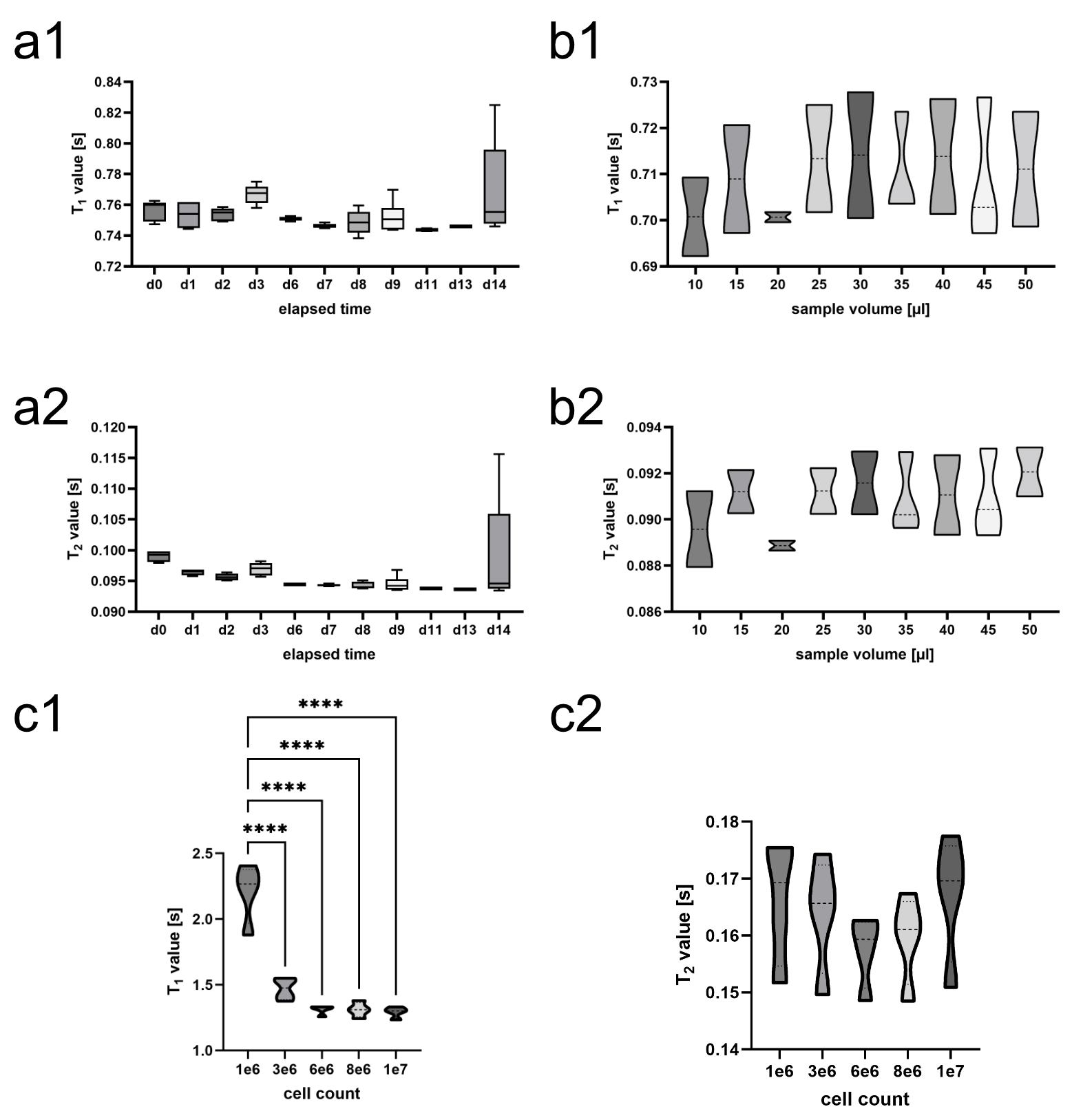

Supplement: S4 Fig — Weighted centroids were calculated for the data shown in Fig 1. The calculated T1 and T2 values were compared to statistically test the previously postulated hypothesis based on the visual impression of the plotted data. Statistical analysis of ADCP samples measured over a 14-day period did not reveal statistically significant differences in either T1 (a1) or T2 values. The same was true for the comparison of the studied sample volume. No significant differences were found for both ADCP volumes studied (T1—b1; T2—b2). To also validate the cell number used, the weighted centroids for the respective samples from S3 Fig were calculated and analyzed. The T1 values for 1e6 cells yielded significantly higher values compared with any other cell number (c1). The respective T2 values did not show significant differences (c2). This analysis confirmed the first visual impression of the data. (TIFF) [file pcbi.1010842.s004.tiff]

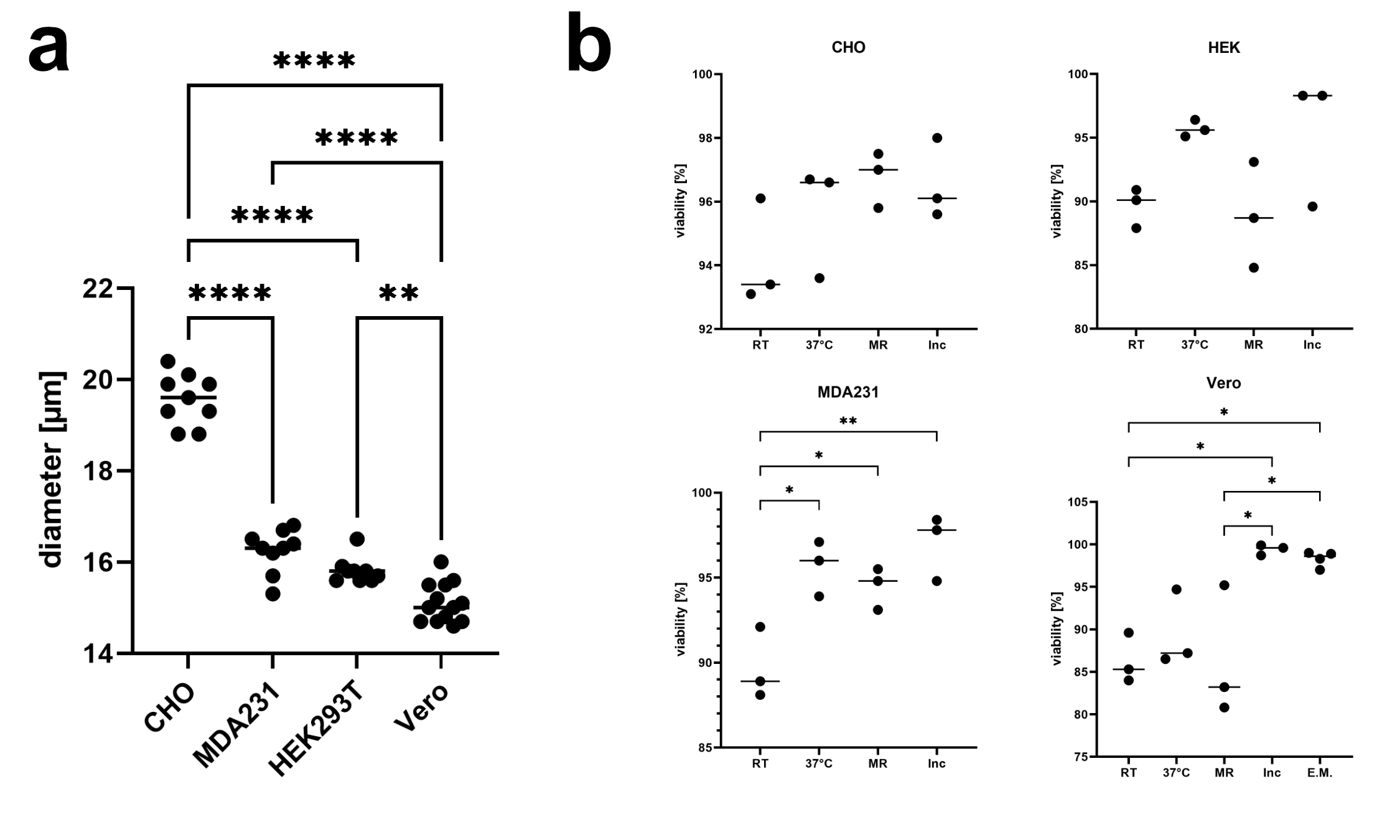

Supplement: S5 Fig — During the automatic evaluation of cell viability, the diameter of the cells was also measured. The statistics showed several significant differences between the different diameters. CHO cells had the largest diameter, while Vero cells had the smallest. Since cell diameter directly correlates with the size of the cell pellet produced for the 5e6 cells, this also provides information about the ratio of cells to media. However, since the CHO cells have significantly higher viability compared to the Vero cells (Fig 2), cell diameter does not appear to have a decisive effect on cell viability in the culture method presented. The individual viability measurements (b) provide detailed insights into the scatter of the data for each cell type. *: P ≤ 0.05 / **: P ≤ 0.01 / ***: P ≤ 0.001 / ****: P ≤ 0.0001 (TIFF) [file pcbi.1010842.s005.tiff]

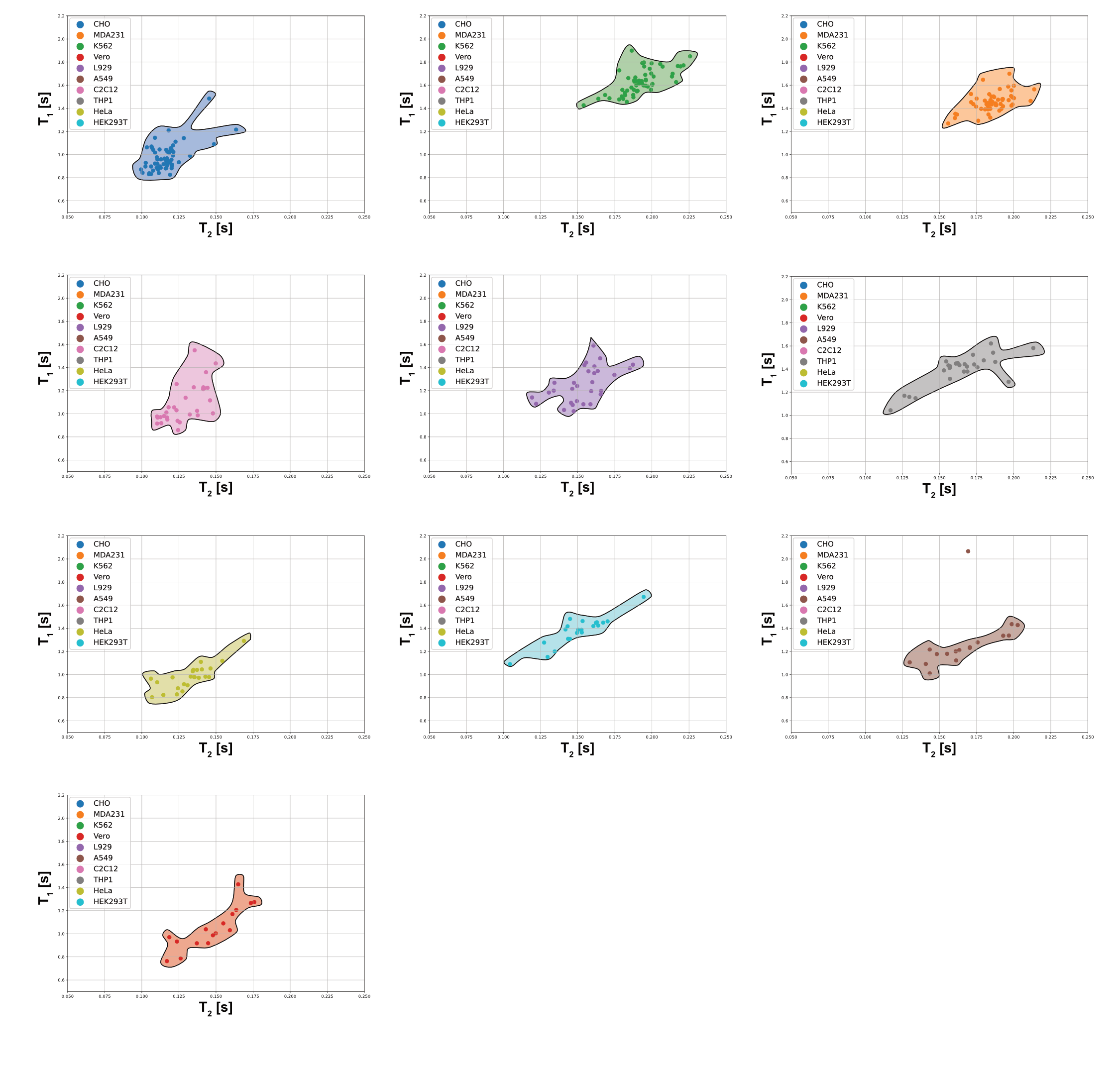

Supplement: S6 Fig — When calculating the weighted centroids for each cell peak of the measured data and creating separate scatter plots for each measured cell line, the local orientation and distribution became apparent. Most cell lines showed an elongated distribution along the T2 axis. Some, such as C2C12 or MDA231, were more locally aggregated. (TIFF) [file pcbi.1010842.s006.tiff]

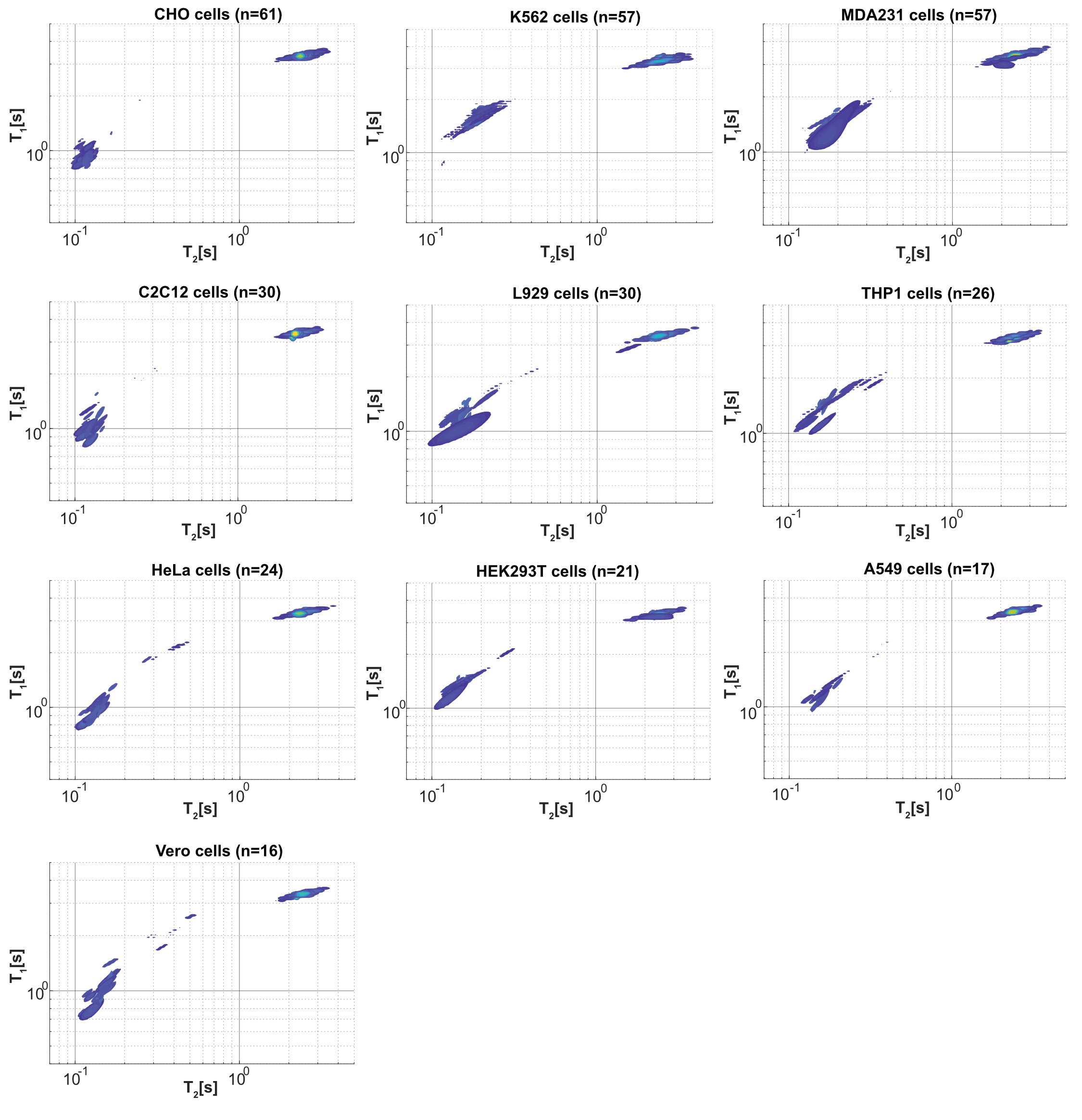

Supplement: S7 Fig — Plotting all spectra recorded for one cell type on a contour plot reveals the two groups of peaks described previously. The media peak was ≈ 2–3 s/3-4 s T1/T2, whereas the position of the cell peaks varied a little for each cell type. Some of the cells used showed a more densely packed group of cell peaks such as the CHO, C2C12, MSC, and A549 cells, whereas others were distributed over a wider range of T1 and T2 values such as HEK293T, HeLa, K562, L929, MDA231, THP1, and Vero cells. Each plot consisted of all measured spectra for the respective cell type as superimposed contour plots. (TIFF) [file pcbi.1010842.s007.tiff]

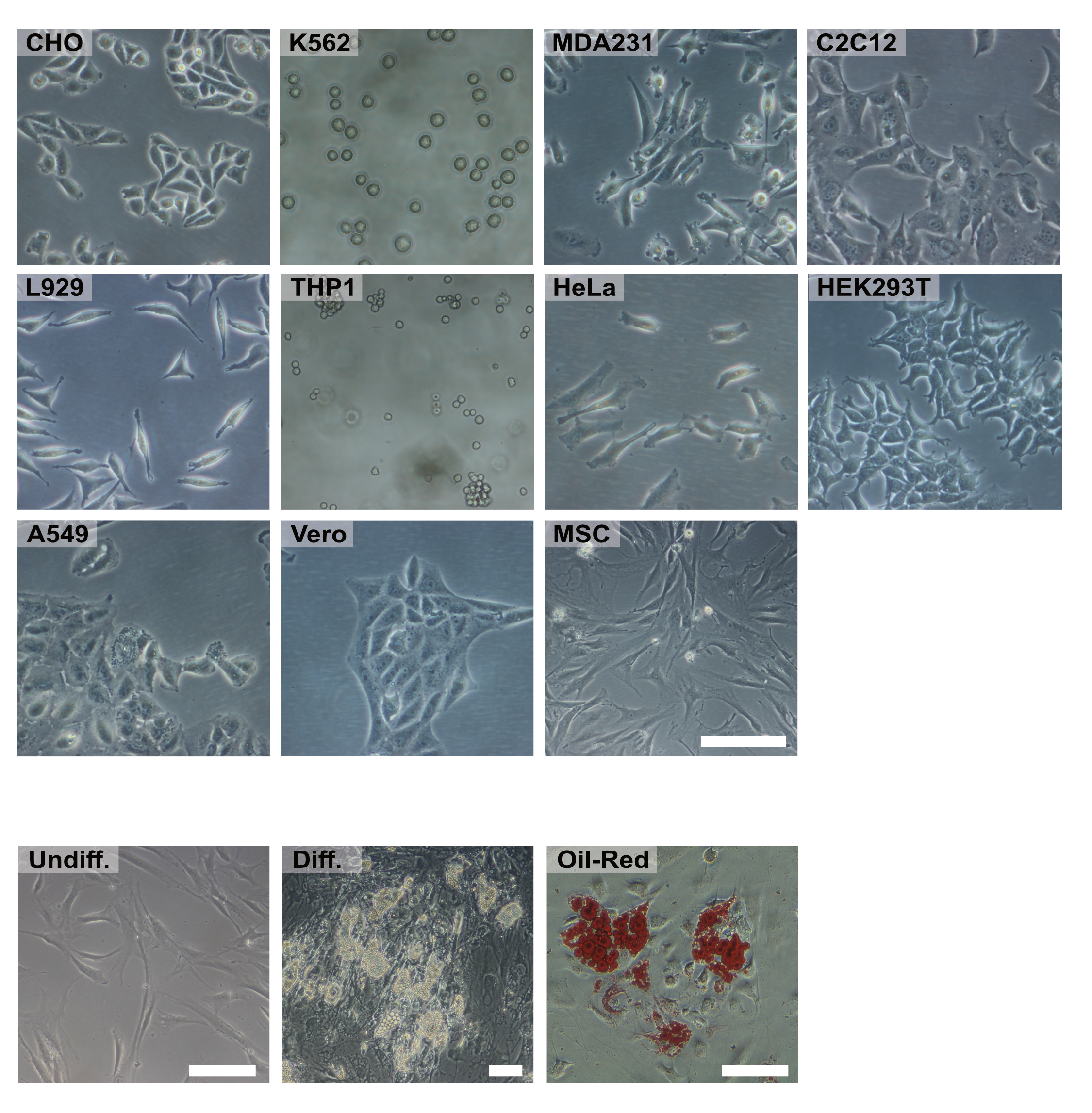

Supplement: S8 Fig — All eleven cells used in this study were classically grown in a two-dimensional monoculture. K562 and THP1 cells were suspension cells and therefore did not adhere to the surface of the culture flask. All other cells adhered to the surface. Some cells such as A549, C2C12, CHO, Vero, and HEK293T had a more compact appearance, while HeLa, L929, MDA231, and MSCs had more elongated phenotypes. The scale bar corresponds to 100 μm. (TIFF) [file pcbi.1010842.s008.tiff]

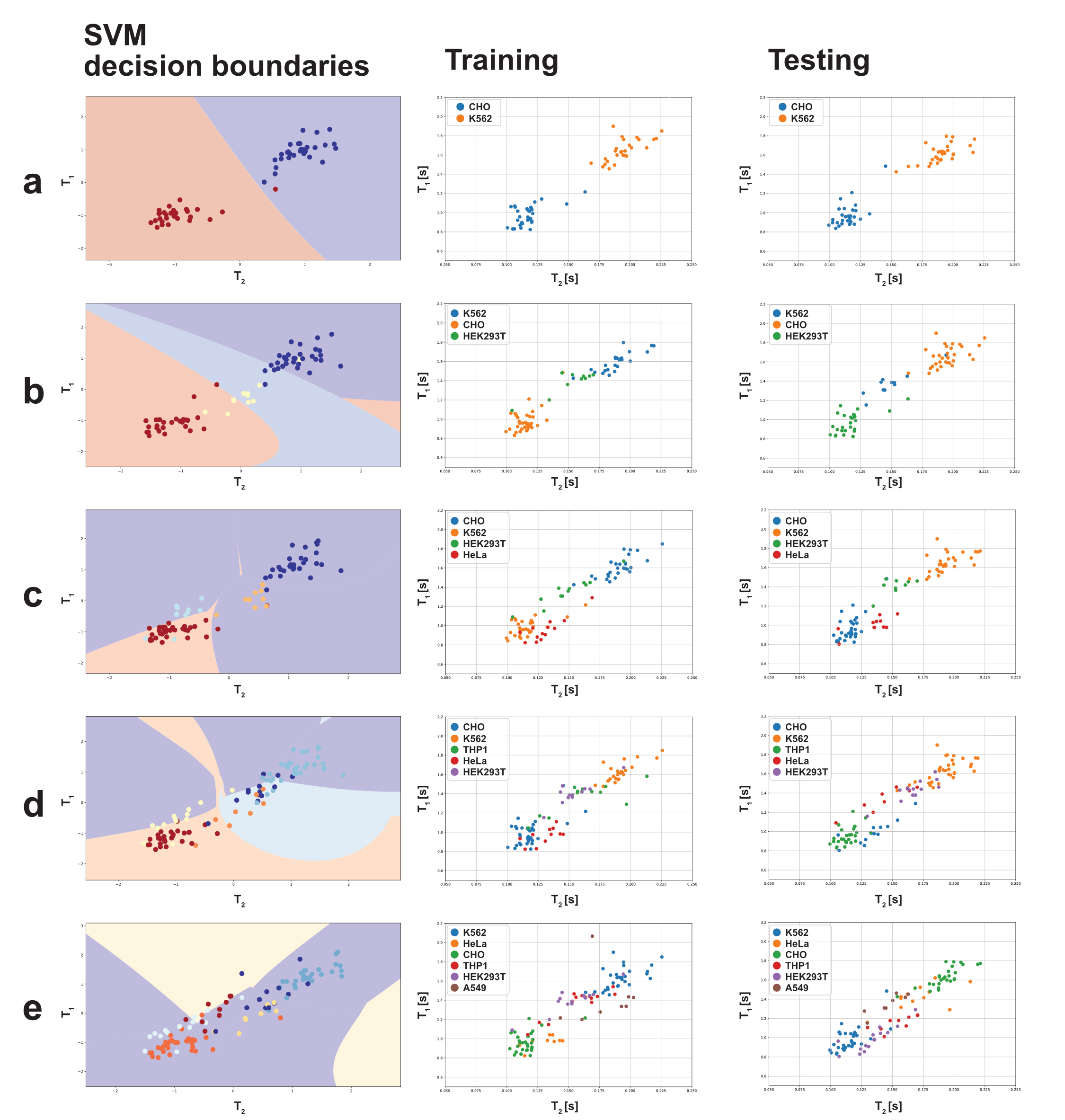

Supplement: S9 Fig — For each setup of SVM, the decision boundaries used by the system were plotted along with the corresponding training and test data. The decision boundaries show that the systems struggle to make appropriate decisions to partition the training data into the appropriate number of classes defined by the number of input cells. Plotting the training and test data provided evidence that the SVM algorithm was indeed trained on the training data only and that the test data indeed represented previously unseen values. A train-test split ratio of 0.5 was chosen for the SVM. For each cell combination, the system was run 300 times. The visualization shows only an example representation of the decision boundaries together with an instance of the training and test data. (TIFF) [file pcbi.1010842.s009.tiff]

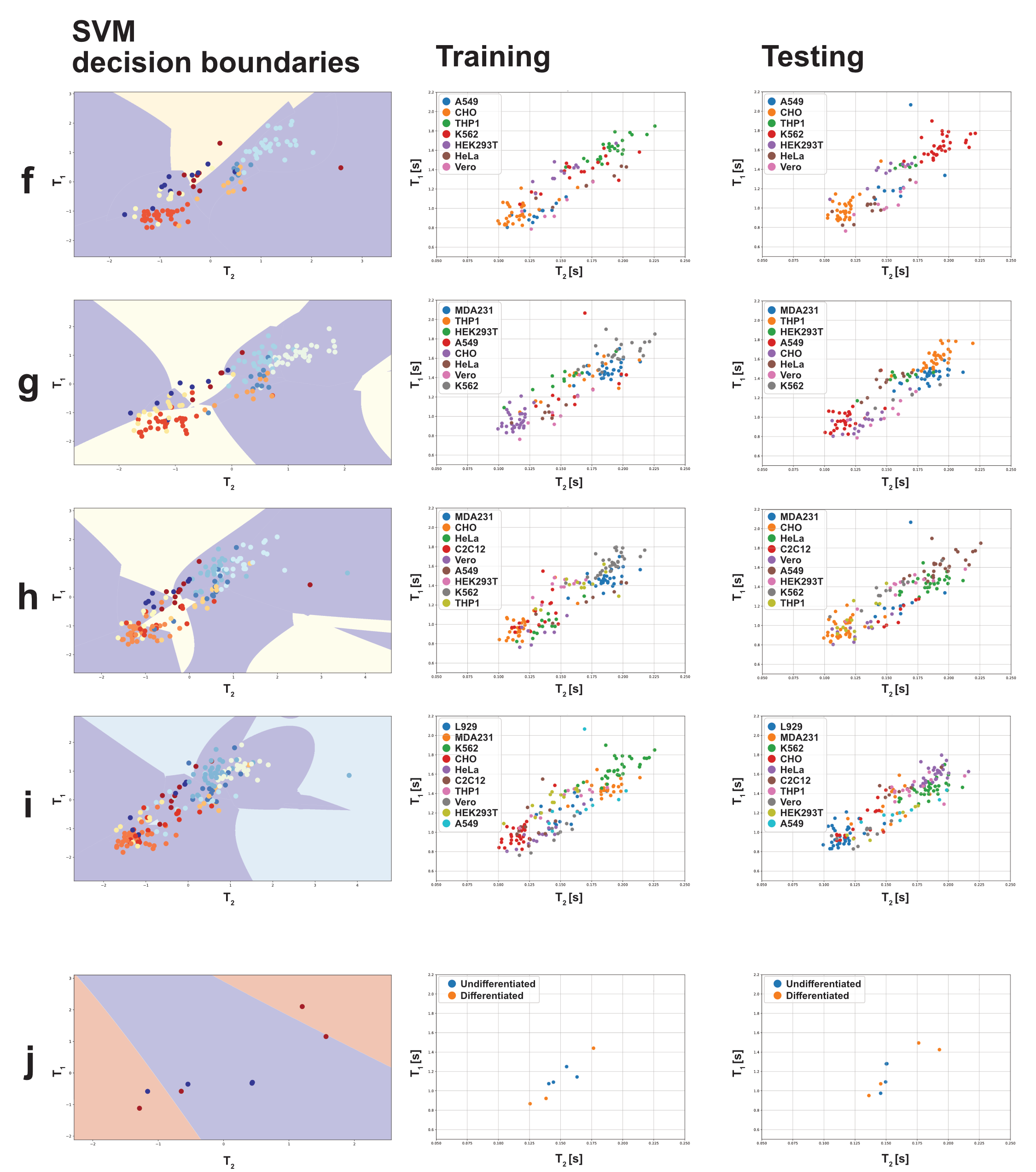

Supplement: S10 Fig — For each setup of SVM, the decision boundaries used by the system were plotted along with the corresponding training and test data. The decision boundaries show that the systems struggle to make appropriate decisions to partition the training data into the appropriate number of classes defined by the number of input cells. Plotting the training and test data provided evidence that the SVM algorithm was indeed trained on the training data only and that the test data indeed represented previously unseen values. A train-test split ratio of 0.5 was chosen for the SVM. For each cell combination, the system was run 300 times. The visualization shows only an example representation of the decision boundaries together with an instance of the training and test data. (TIFF) [file pcbi.1010842.s010.tiff]

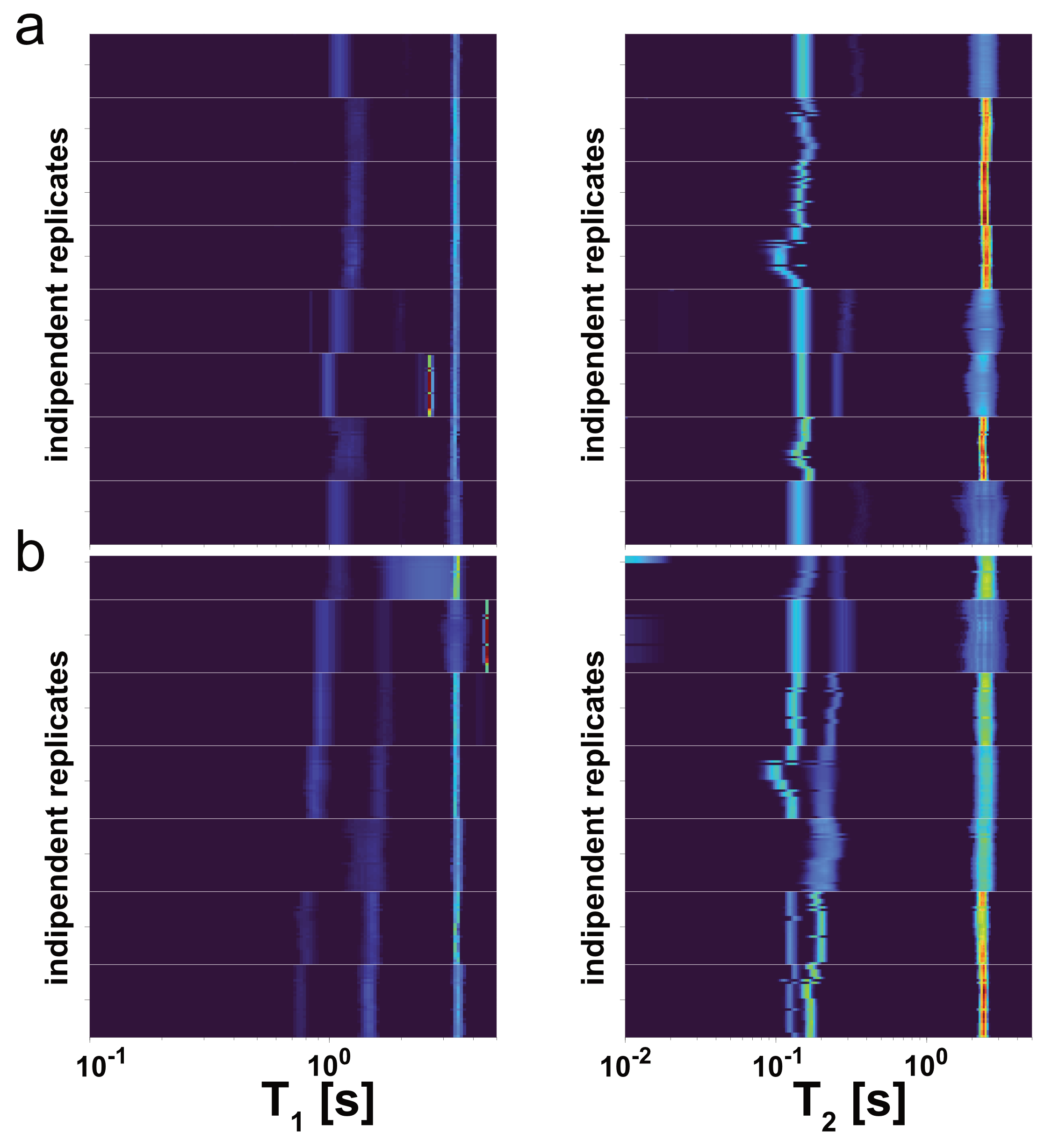

Supplement: S11 Fig — Each MSC measurement was augmented by a factor of 30. When the augmented spectra are plotted on top of each other, the effects produced become visible. Therefore, each area separated by white lines consist of 30 individual spectra. Due to the logarithmic scaling, the augmentation had a stronger effect on the depiction of the signal peaks at lower T1 and T2 times than on those at higher relaxation times. The spectra of the undifferentiated MSCs (a) showed only two peaks in T1 and T2, whereas the spectra of the differentiated MSCs (b) mostly showed three peaks. The lines in the diagrams represent the groups of 30 belonging to an original measurement. (TIFF) [file pcbi.1010842.s011.tiff]

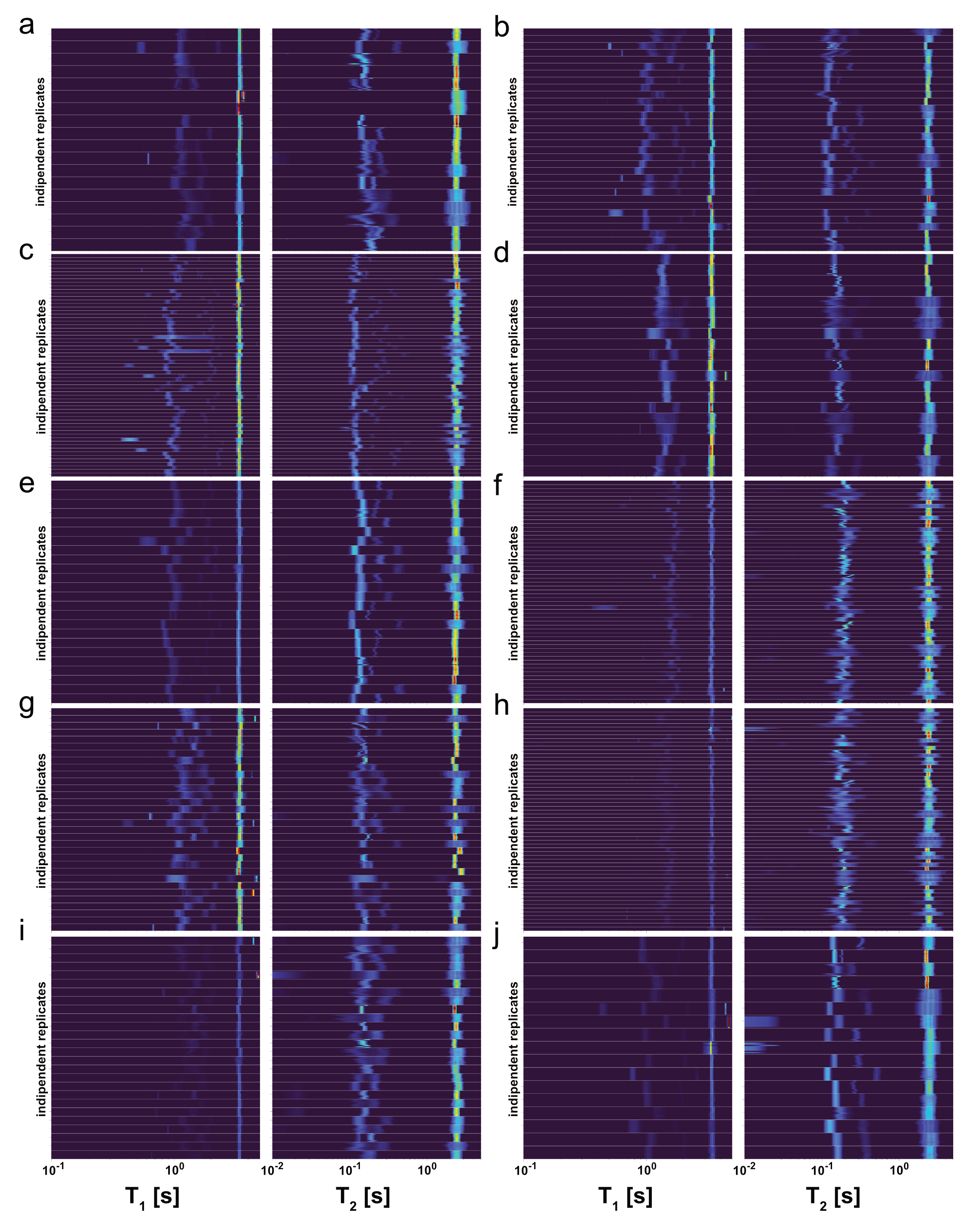

Supplement: S12 Fig — The effects of the position shift aspect of the data augmentation were more pronounced for the cell peaks due to their shorter relaxation times. This was similar for A549 (a; nb = 17), C2C12 (b; nb = 30), CHO (c; nb = 61), HEK293T (d; nb = 21), HeLa (e; nb = 24), K562 (f; nb = 57), L929 (g; nb = 30), MDA231 (h; nb = 57), THP1 (i; nb = 26), and Vero (j; nb = 16) cells. The plots shown also demonstrate the stability of the media peak across all ten cell lines measured. Shown are plots with an augmentation factor of 10. The lines drawn delineate each cluster of augmented data derived from the first measurement. The number of clusters in the plots is different for each cell line due to the different number of measurements. (TIFF) [file pcbi.1010842.s012.tiff]

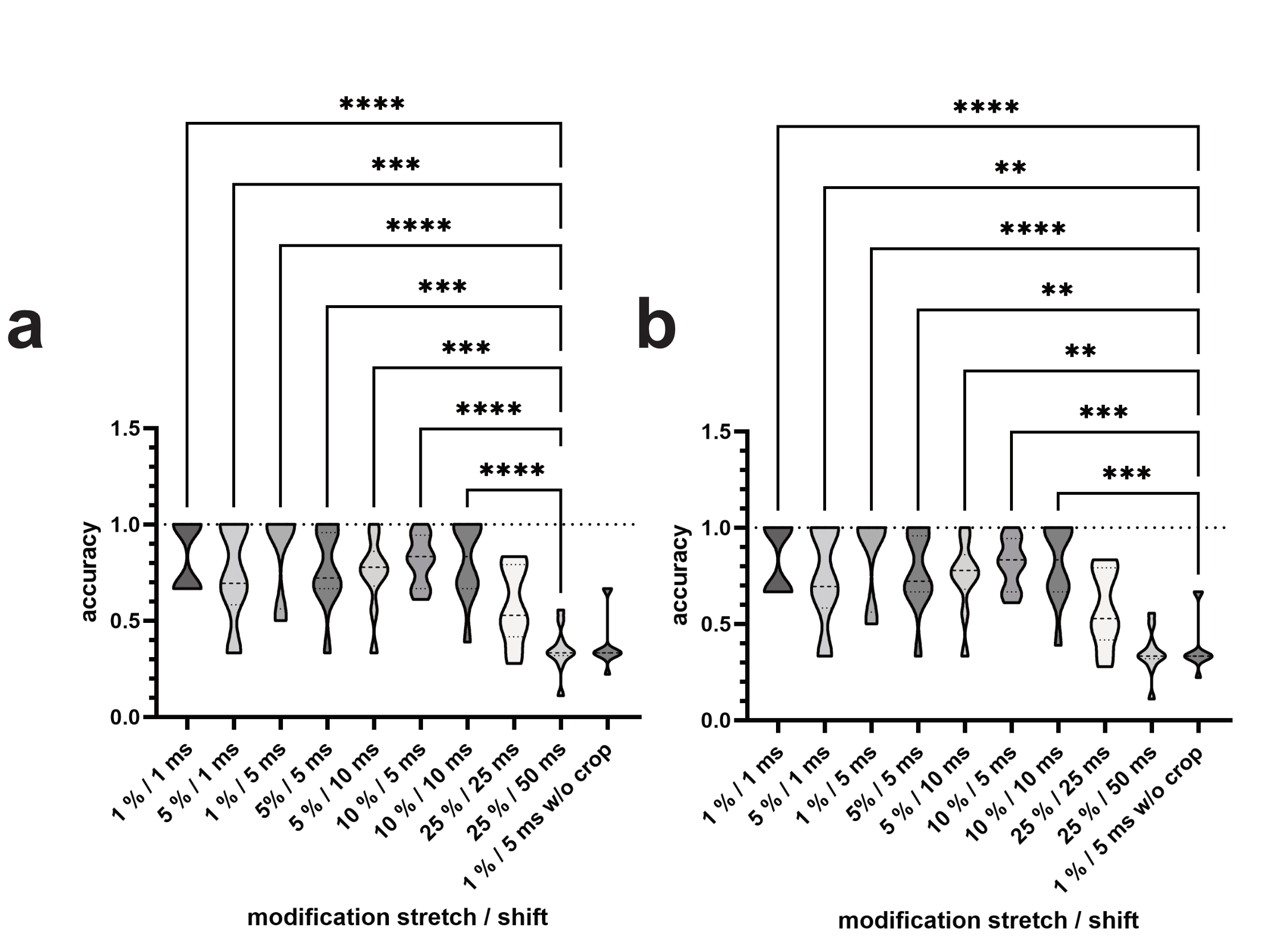

Supplement: S13 Fig — In addition to the augmentation values of 1% - 10% stretch and 1 ms—10 ms shift already shown, other high augmentation values were tested. 25% / 50 ms showed no significant differences from other augmentation values, while 25% / 50 ms was significantly different from any other reported augmentation value (a). Because 1% / 5 ms had the highest mean value (85%), it was compared with performance for the same data set but without the media peak cropping. The statistics showed that except for the augmentation for 25% / 50 ms, the performance without cropping to 0–3.0079 s for T1 and 0–0.4062 s for T2 showed significant differences from all other augmentation values. This suggests that the media peak reduces the accuracy of the ANN when trained on MSC data. *: P ≤ 0.05 / **: P ≤ 0.01 / ***: P ≤ 0.001 / ****: P ≤ 0.0001 (TIFF) [file pcbi.1010842.s013.tiff]

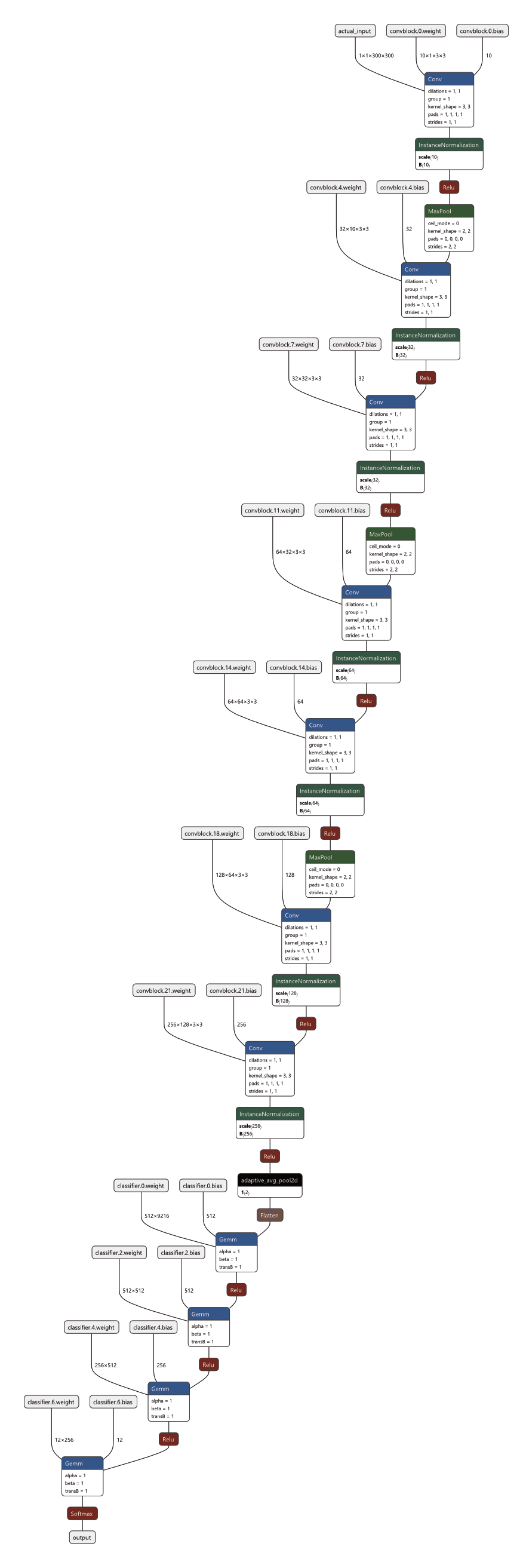

Supplement: S14 Fig — The architecture was based on a modified VGG neural network whose parameters were optimized to achieve the highest accuracy on the available data. The resulting structure consisted of seven convolutional layers followed by four dense layers. Normalization and maxpooling layers were inserted between the convolutional layers. Relu was chosen as the activation function and Adamax as the optimizer. The architecture ended with a softmax function. Further details can be found in the schematic above. The respective ONNX file from which this schematic was generated has been attached to this publication. (TIFF) [file pcbi.1010842.s014.tiff]

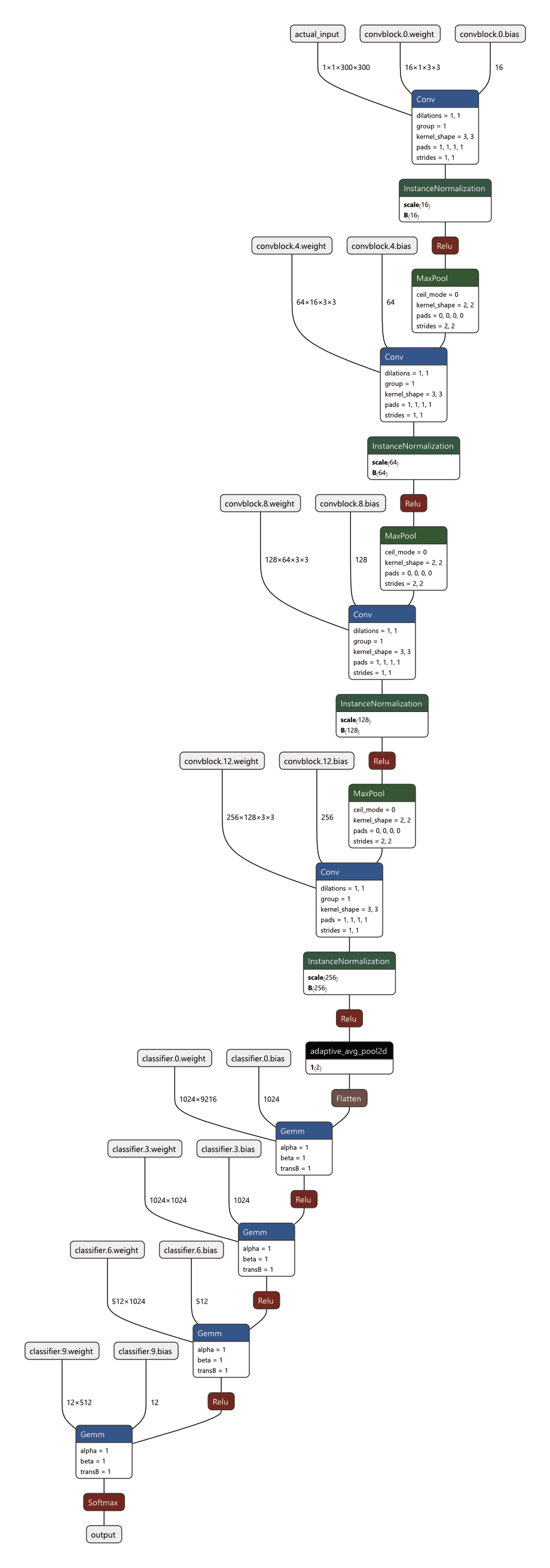

Supplement: S15 Fig — The architecture was based on a modified VGG neural network whose parameters were optimized to achieve the highest accuracy on the available data. It consisted of four convolutional layers separated by normalization and maxpooling layers. The convolutional layers were followed by four dense layers. Relu was used as the activation function and Adamax as an optimizer. To reduce possible overfitting effects, a dropout of 0.25 was applied to the dense layers. The architecture ended with a softmax function. Further details can be found in the schematic above. The respective ONNX file from which this schematic was generated has been attached to this publication. (TIFF) [file pcbi.1010842.s015.tiff]

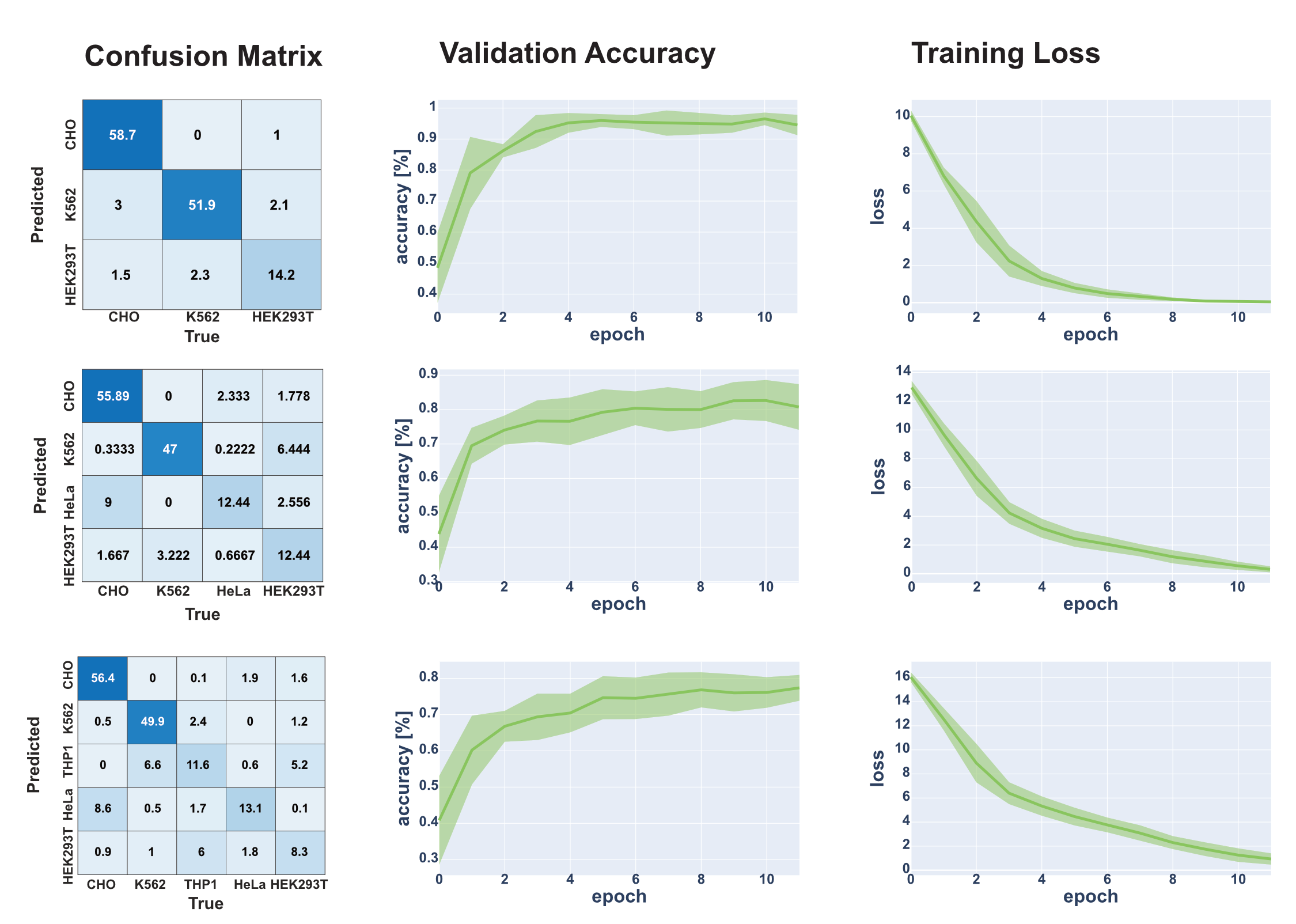

Supplement: S16 Fig — Confusion matrices for the final ANN test (values averaged over ten replicates) demonstrate that most cell lines were correctly classified. These observations correlate with the low variance in the power plots. Accuracy reached its plateau after only four to six epochs. Training loss converged as expected with low variance (shaded area) between the ten independent replicates. All other data can be exported from the attached tensorboard data files. (TIFF) [file pcbi.1010842.s016.tiff]

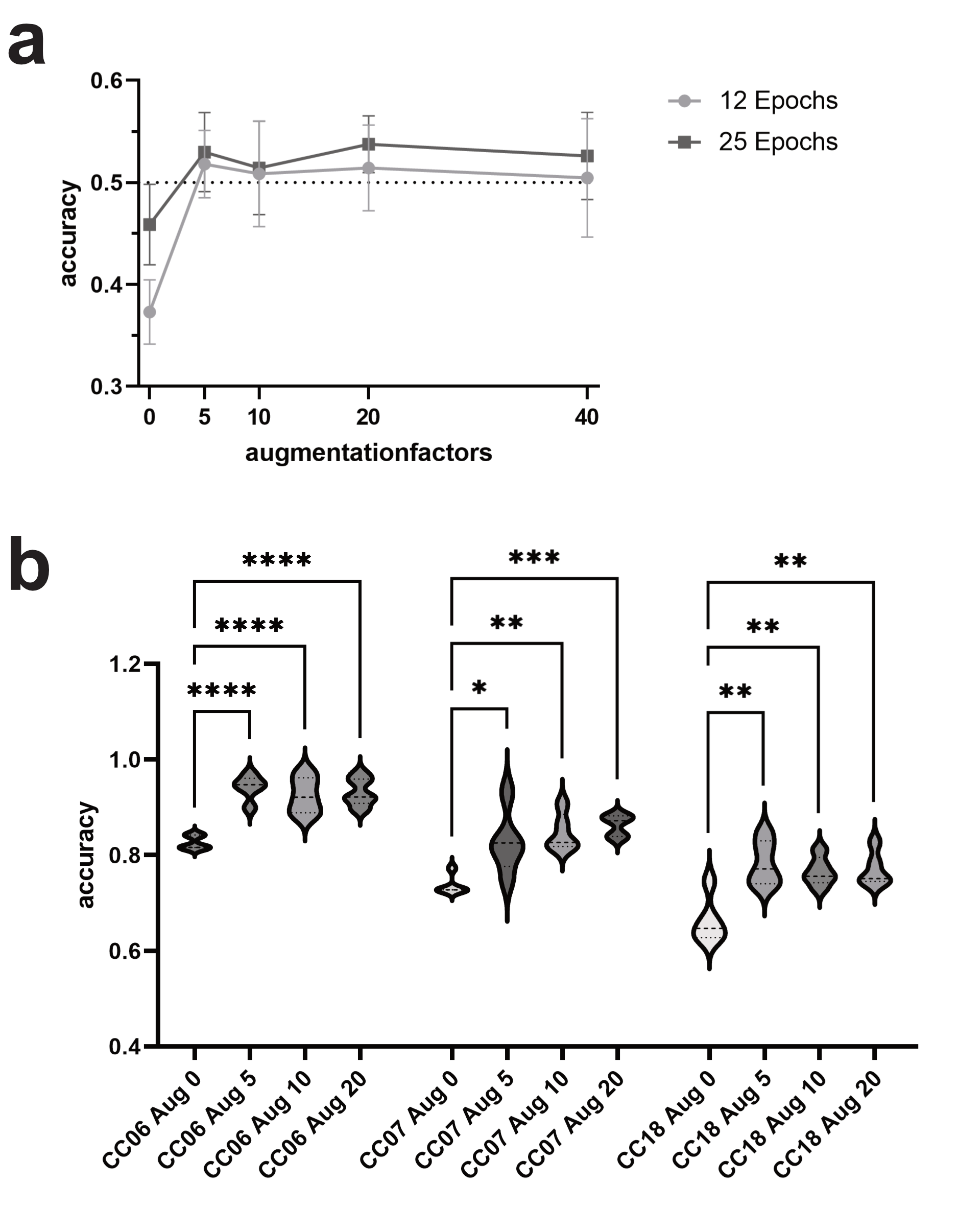

Supplement: S17 Fig — When comparing two different epochs with augmentation factors of 0, 5, 10, 20, and 40, the statistics showed that there were no significant differences between a training duration of 12 and 25 epochs (a). When further comparing different augmentation factors for different cell compositions (b), the statistics showed the previously described pattern that any augmentation factor greater than zero was significantly better than no augmentation (augmentation factor = 0). This was consistent across several different cell compositions. CC06: CHO, K562, HEK293T / CC07: CHO, K562, HEK293T, HeLa / CC18: CHO, K562, HEK293T, HeLa, THP1; *: ≤ 0.05 / **: P ≤ 0.01 / ***: P ≤ 0.001 / ****: P ≤ 0.0001 (TIFF) [file pcbi.1010842.s017.tiff]

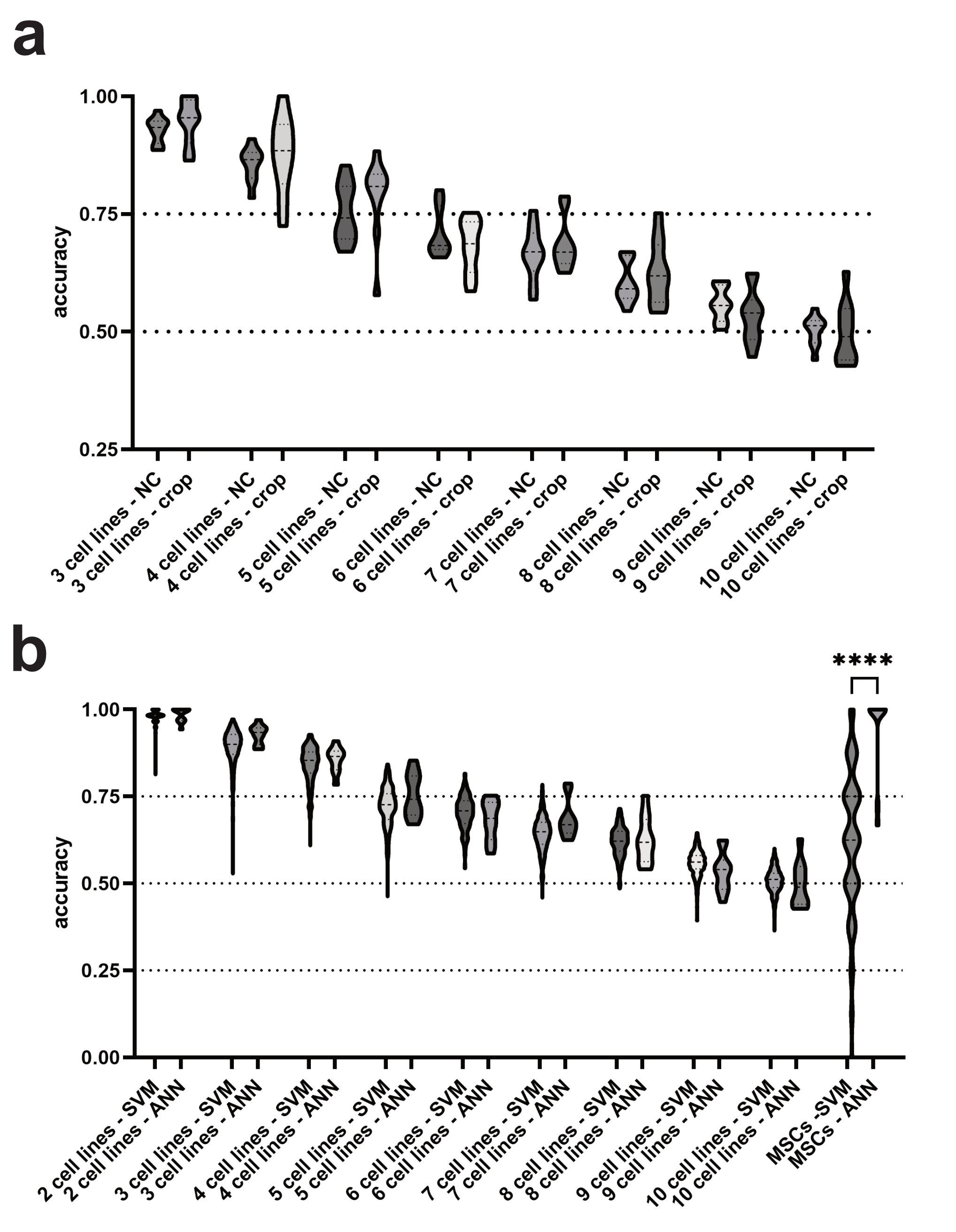

Supplement: S18 Fig — When examining the effect of cropped and uncropped data on ANN performance (a), the statistics showed no significant differences between the two conditions. Similar observations were made when comparing SVM performance with ANN performance (b). No significant differences were found except for the MSCs. The performance of the SVM compared with the ANN for the classification of MSC differentiation was subject to significantly higher scatter. While the ANN only showed results with high accuracies, the SVM gave highly variable and therefore unreliable results between 0 and 100%. Each comparison was performed for the combinations of cell lines that yielded the highest average accuracy. All measurements were carried out ten indipendent times. ****: P ≤ 0.0001 (TIFF) [file pcbi.1010842.s018.tiff]

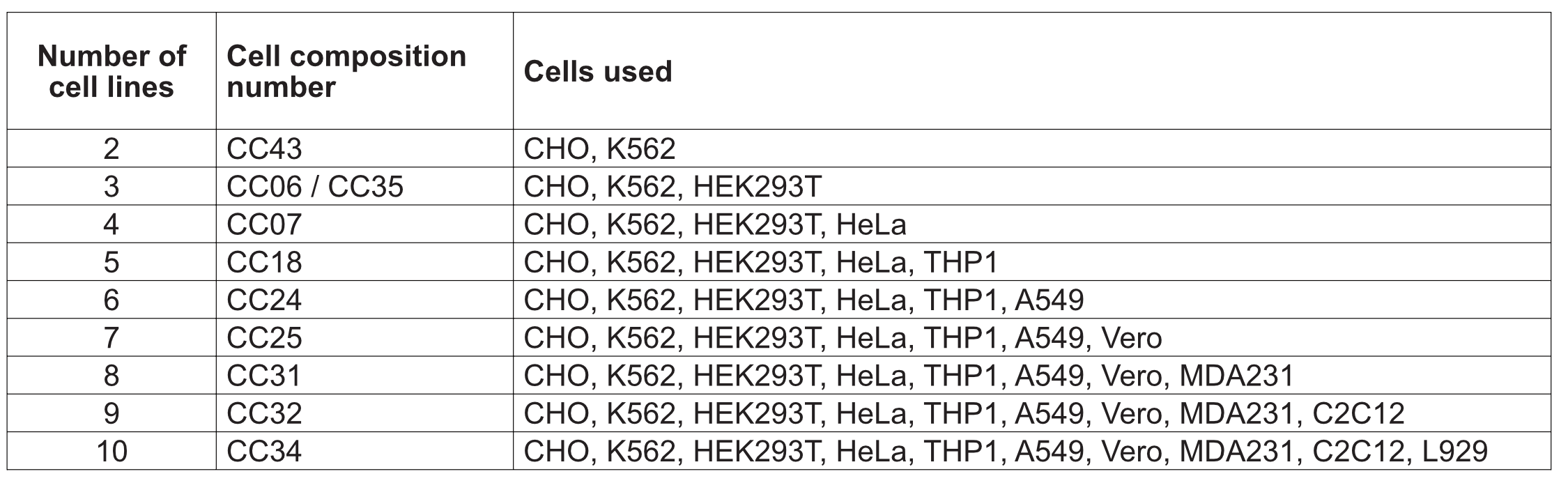

Supplement: S1 Table — The following cell combinations represent the optimal compositions for the respective number of incorporated cells for SVM and ANN training. (TIFF) [file pcbi.1010842.s019.tiff]

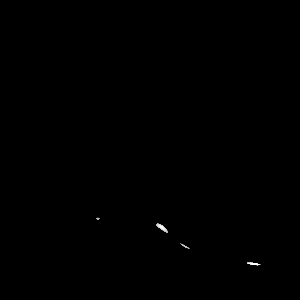

Supplement: S6 Data — The data is saved as *.png files. The subfolder entitled ‘0’ represent the undifferentiated cells while ‘1’ represent the differentiated MSCs. Every dataset also includes the original data, that were not affected by the augmentation algorithm (refer to tag ‘_Original.png’). (ZIP) [file pcbi.1010842.s025.zip › S6 Data/MSCs/MSC_AugIter-0_0Stretch_0Shift/0/MSC-1_Original.png]

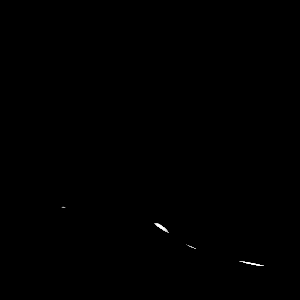

Supplement: S6 Data — The data is saved as *.png files. The subfolder entitled ‘0’ represent the undifferentiated cells while ‘1’ represent the differentiated MSCs. Every dataset also includes the original data, that were not affected by the augmentation algorithm (refer to tag ‘_Original.png’). (ZIP) [file pcbi.1010842.s025.zip › S6 Data/MSCs/MSC_AugIter-0_0Stretch_0Shift/0/MSC-10_Original.png]

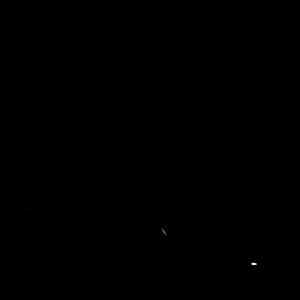

Supplement: S6 Data — The data is saved as *.png files. The subfolder entitled ‘0’ represent the undifferentiated cells while ‘1’ represent the differentiated MSCs. Every dataset also includes the original data, that were not affected by the augmentation algorithm (refer to tag ‘_Original.png’). (ZIP) [file pcbi.1010842.s025.zip › S6 Data/MSCs/MSC_AugIter-0_0Stretch_0Shift/0/MSC-2_Original.png]

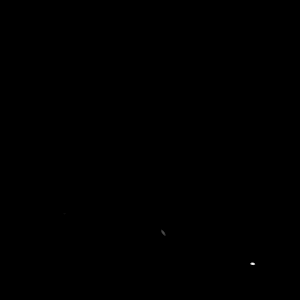

Supplement: S6 Data — The data is saved as *.png files. The subfolder entitled ‘0’ represent the undifferentiated cells while ‘1’ represent the differentiated MSCs. Every dataset also includes the original data, that were not affected by the augmentation algorithm (refer to tag ‘_Original.png’). (ZIP) [file pcbi.1010842.s025.zip › S6 Data/MSCs/MSC_AugIter-0_0Stretch_0Shift/0/MSC-3_Original.png]

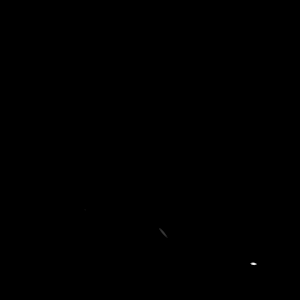

Supplement: S6 Data — The data is saved as *.png files. The subfolder entitled ‘0’ represent the undifferentiated cells while ‘1’ represent the differentiated MSCs. Every dataset also includes the original data, that were not affected by the augmentation algorithm (refer to tag ‘_Original.png’). (ZIP) [file pcbi.1010842.s025.zip › S6 Data/MSCs/MSC_AugIter-0_0Stretch_0Shift/0/MSC-7_Original.png]

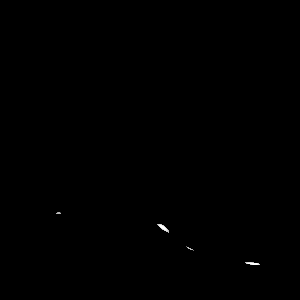

Supplement: S6 Data — The data is saved as *.png files. The subfolder entitled ‘0’ represent the undifferentiated cells while ‘1’ represent the differentiated MSCs. Every dataset also includes the original data, that were not affected by the augmentation algorithm (refer to tag ‘_Original.png’). (ZIP) [file pcbi.1010842.s025.zip › S6 Data/MSCs/MSC_AugIter-0_0Stretch_0Shift/0/MSC-9_Original.png]

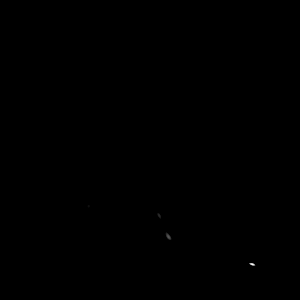

Supplement: S6 Data — The data is saved as *.png files. The subfolder entitled ‘0’ represent the undifferentiated cells while ‘1’ represent the differentiated MSCs. Every dataset also includes the original data, that were not affected by the augmentation algorithm (refer to tag ‘_Original.png’). (ZIP) [file pcbi.1010842.s025.zip › S6 Data/MSCs/MSC_AugIter-0_0Stretch_0Shift/1/DiffMSC-11_Original.png]

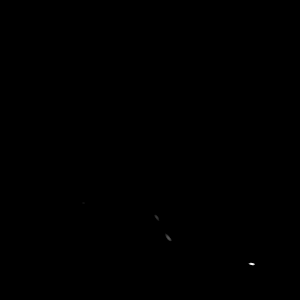

Supplement: S6 Data — The data is saved as *.png files. The subfolder entitled ‘0’ represent the undifferentiated cells while ‘1’ represent the differentiated MSCs. Every dataset also includes the original data, that were not affected by the augmentation algorithm (refer to tag ‘_Original.png’). (ZIP) [file pcbi.1010842.s025.zip › S6 Data/MSCs/MSC_AugIter-0_0Stretch_0Shift/1/DiffMSC-12_Original.png]

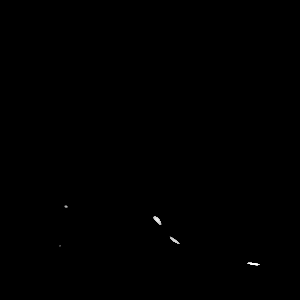

Supplement: S6 Data — The data is saved as *.png files. The subfolder entitled ‘0’ represent the undifferentiated cells while ‘1’ represent the differentiated MSCs. Every dataset also includes the original data, that were not affected by the augmentation algorithm (refer to tag ‘_Original.png’). (ZIP) [file pcbi.1010842.s025.zip › S6 Data/MSCs/MSC_AugIter-0_0Stretch_0Shift/1/DiffMSC-4_Original.png]

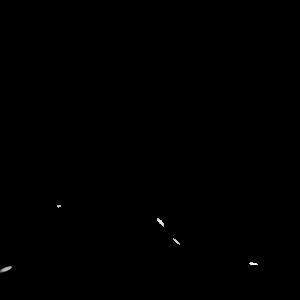

Supplement: S6 Data — The data is saved as *.png files. The subfolder entitled ‘0’ represent the undifferentiated cells while ‘1’ represent the differentiated MSCs. Every dataset also includes the original data, that were not affected by the augmentation algorithm (refer to tag ‘_Original.png’). (ZIP) [file pcbi.1010842.s025.zip › S6 Data/MSCs/MSC_AugIter-0_0Stretch_0Shift/1/DiffMSC-5_Original.png]

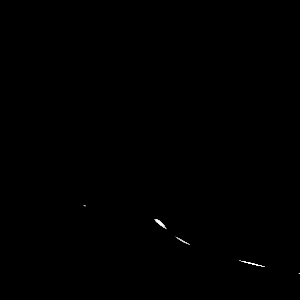

Supplement: S6 Data — The data is saved as *.png files. The subfolder entitled ‘0’ represent the undifferentiated cells while ‘1’ represent the differentiated MSCs. Every dataset also includes the original data, that were not affected by the augmentation algorithm (refer to tag ‘_Original.png’). (ZIP) [file pcbi.1010842.s025.zip › S6 Data/MSCs/MSC_AugIter-0_0Stretch_0Shift/1/DiffMSC-6_Original.png]

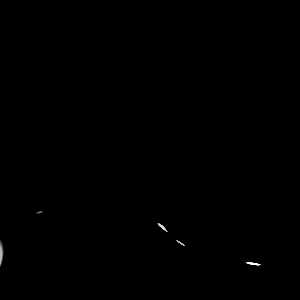

Supplement: S6 Data — The data is saved as *.png files. The subfolder entitled ‘0’ represent the undifferentiated cells while ‘1’ represent the differentiated MSCs. Every dataset also includes the original data, that were not affected by the augmentation algorithm (refer to tag ‘_Original.png’). (ZIP) [file pcbi.1010842.s025.zip › S6 Data/MSCs/MSC_AugIter-0_0Stretch_0Shift/1/DiffMSC-8_Original.png]

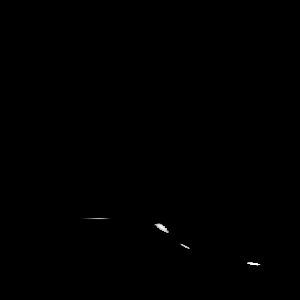

Supplement: S6 Data — The data is saved as *.png files. The subfolder entitled ‘0’ represent the undifferentiated cells while ‘1’ represent the differentiated MSCs. Every dataset also includes the original data, that were not affected by the augmentation algorithm (refer to tag ‘_Original.png’). (ZIP) [file pcbi.1010842.s025.zip › S6 Data/MSCs/MSC_AugIter-40_10Stretch_10Shift/0/MSC-1_1.png]

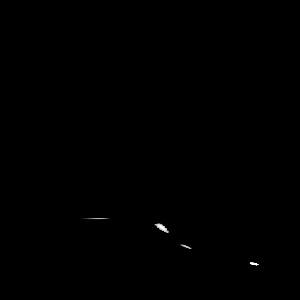

Supplement: S6 Data — The data is saved as *.png files. The subfolder entitled ‘0’ represent the undifferentiated cells while ‘1’ represent the differentiated MSCs. Every dataset also includes the original data, that were not affected by the augmentation algorithm (refer to tag ‘_Original.png’). (ZIP) [file pcbi.1010842.s025.zip › S6 Data/MSCs/MSC_AugIter-40_10Stretch_10Shift/0/MSC-1_10.png]

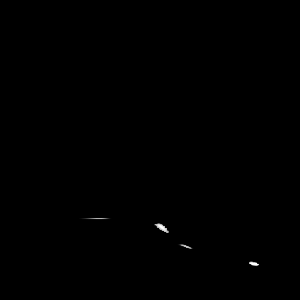

Supplement: S6 Data — The data is saved as *.png files. The subfolder entitled ‘0’ represent the undifferentiated cells while ‘1’ represent the differentiated MSCs. Every dataset also includes the original data, that were not affected by the augmentation algorithm (refer to tag ‘_Original.png’). (ZIP) [file pcbi.1010842.s025.zip › S6 Data/MSCs/MSC_AugIter-40_10Stretch_10Shift/0/MSC-1_11.png]

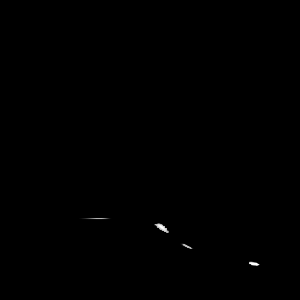

Supplement: S6 Data — The data is saved as *.png files. The subfolder entitled ‘0’ represent the undifferentiated cells while ‘1’ represent the differentiated MSCs. Every dataset also includes the original data, that were not affected by the augmentation algorithm (refer to tag ‘_Original.png’). (ZIP) [file pcbi.1010842.s025.zip › S6 Data/MSCs/MSC_AugIter-40_10Stretch_10Shift/0/MSC-1_12.png]

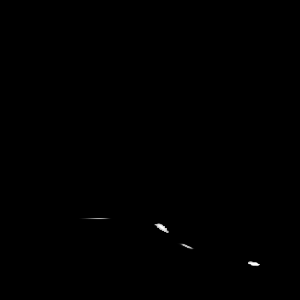

Supplement: S6 Data — The data is saved as *.png files. The subfolder entitled ‘0’ represent the undifferentiated cells while ‘1’ represent the differentiated MSCs. Every dataset also includes the original data, that were not affected by the augmentation algorithm (refer to tag ‘_Original.png’). (ZIP) [file pcbi.1010842.s025.zip › S6 Data/MSCs/MSC_AugIter-40_10Stretch_10Shift/0/MSC-1_13.png]

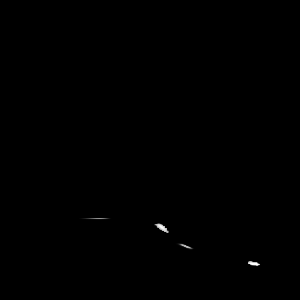

Supplement: S6 Data — The data is saved as *.png files. The subfolder entitled ‘0’ represent the undifferentiated cells while ‘1’ represent the differentiated MSCs. Every dataset also includes the original data, that were not affected by the augmentation algorithm (refer to tag ‘_Original.png’). (ZIP) [file pcbi.1010842.s025.zip › S6 Data/MSCs/MSC_AugIter-40_10Stretch_10Shift/0/MSC-1_14.png]

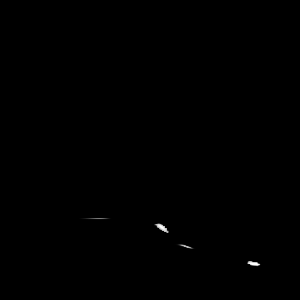

Supplement: S6 Data — The data is saved as *.png files. The subfolder entitled ‘0’ represent the undifferentiated cells while ‘1’ represent the differentiated MSCs. Every dataset also includes the original data, that were not affected by the augmentation algorithm (refer to tag ‘_Original.png’). (ZIP) [file pcbi.1010842.s025.zip › S6 Data/MSCs/MSC_AugIter-40_10Stretch_10Shift/0/MSC-1_15.png]

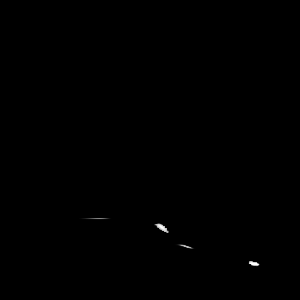

Supplement: S6 Data — The data is saved as *.png files. The subfolder entitled ‘0’ represent the undifferentiated cells while ‘1’ represent the differentiated MSCs. Every dataset also includes the original data, that were not affected by the augmentation algorithm (refer to tag ‘_Original.png’). (ZIP) [file pcbi.1010842.s025.zip › S6 Data/MSCs/MSC_AugIter-40_10Stretch_10Shift/0/MSC-1_16.png]

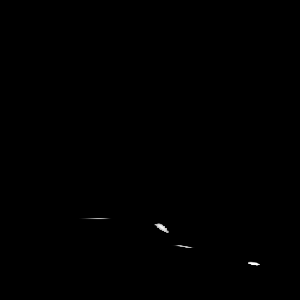

Supplement: S6 Data — The data is saved as *.png files. The subfolder entitled ‘0’ represent the undifferentiated cells while ‘1’ represent the differentiated MSCs. Every dataset also includes the original data, that were not affected by the augmentation algorithm (refer to tag ‘_Original.png’). (ZIP) [file pcbi.1010842.s025.zip › S6 Data/MSCs/MSC_AugIter-40_10Stretch_10Shift/0/MSC-1_17.png]

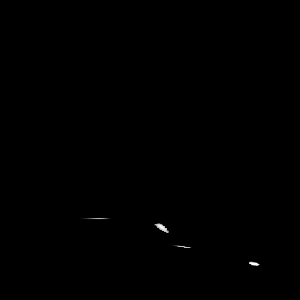

Supplement: S6 Data — The data is saved as *.png files. The subfolder entitled ‘0’ represent the undifferentiated cells while ‘1’ represent the differentiated MSCs. Every dataset also includes the original data, that were not affected by the augmentation algorithm (refer to tag ‘_Original.png’). (ZIP) [file pcbi.1010842.s025.zip › S6 Data/MSCs/MSC_AugIter-40_10Stretch_10Shift/0/MSC-1_18.png]

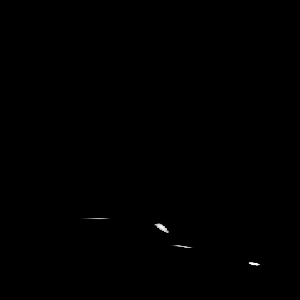

Supplement: S6 Data — The data is saved as *.png files. The subfolder entitled ‘0’ represent the undifferentiated cells while ‘1’ represent the differentiated MSCs. Every dataset also includes the original data, that were not affected by the augmentation algorithm (refer to tag ‘_Original.png’). (ZIP) [file pcbi.1010842.s025.zip › S6 Data/MSCs/MSC_AugIter-40_10Stretch_10Shift/0/MSC-1_19.png]

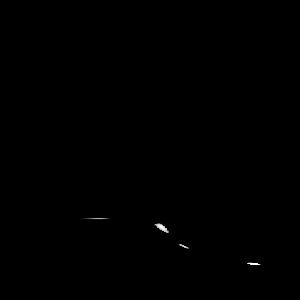

Supplement: S6 Data — The data is saved as *.png files. The subfolder entitled ‘0’ represent the undifferentiated cells while ‘1’ represent the differentiated MSCs. Every dataset also includes the original data, that were not affected by the augmentation algorithm (refer to tag ‘_Original.png’). (ZIP) [file pcbi.1010842.s025.zip › S6 Data/MSCs/MSC_AugIter-40_10Stretch_10Shift/0/MSC-1_2.png]

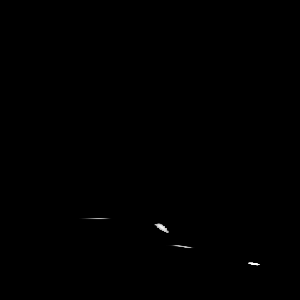

Supplement: S6 Data — The data is saved as *.png files. The subfolder entitled ‘0’ represent the undifferentiated cells while ‘1’ represent the differentiated MSCs. Every dataset also includes the original data, that were not affected by the augmentation algorithm (refer to tag ‘_Original.png’). (ZIP) [file pcbi.1010842.s025.zip › S6 Data/MSCs/MSC_AugIter-40_10Stretch_10Shift/0/MSC-1_20.png]

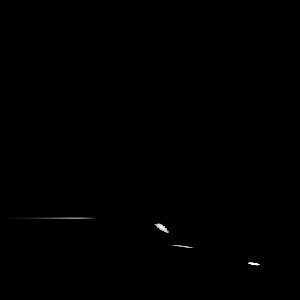

Supplement: S6 Data — The data is saved as *.png files. The subfolder entitled ‘0’ represent the undifferentiated cells while ‘1’ represent the differentiated MSCs. Every dataset also includes the original data, that were not affected by the augmentation algorithm (refer to tag ‘_Original.png’). (ZIP) [file pcbi.1010842.s025.zip › S6 Data/MSCs/MSC_AugIter-40_10Stretch_10Shift/0/MSC-1_21.png]

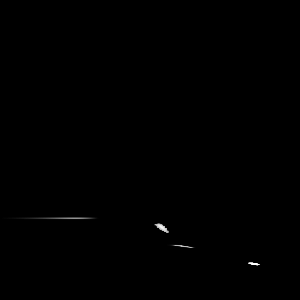

Supplement: S6 Data — The data is saved as *.png files. The subfolder entitled ‘0’ represent the undifferentiated cells while ‘1’ represent the differentiated MSCs. Every dataset also includes the original data, that were not affected by the augmentation algorithm (refer to tag ‘_Original.png’). (ZIP) [file pcbi.1010842.s025.zip › S6 Data/MSCs/MSC_AugIter-40_10Stretch_10Shift/0/MSC-1_22.png]

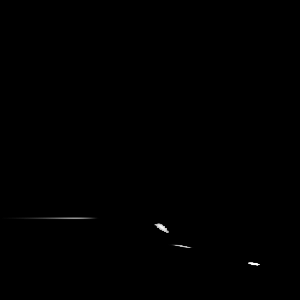

Supplement: S6 Data — The data is saved as *.png files. The subfolder entitled ‘0’ represent the undifferentiated cells while ‘1’ represent the differentiated MSCs. Every dataset also includes the original data, that were not affected by the augmentation algorithm (refer to tag ‘_Original.png’). (ZIP) [file pcbi.1010842.s025.zip › S6 Data/MSCs/MSC_AugIter-40_10Stretch_10Shift/0/MSC-1_23.png]

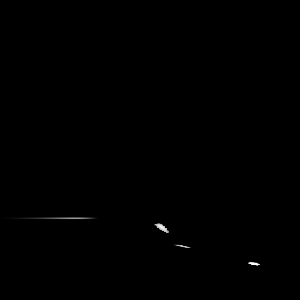

Supplement: S6 Data — The data is saved as *.png files. The subfolder entitled ‘0’ represent the undifferentiated cells while ‘1’ represent the differentiated MSCs. Every dataset also includes the original data, that were not affected by the augmentation algorithm (refer to tag ‘_Original.png’). (ZIP) [file pcbi.1010842.s025.zip › S6 Data/MSCs/MSC_AugIter-40_10Stretch_10Shift/0/MSC-1_24.png]

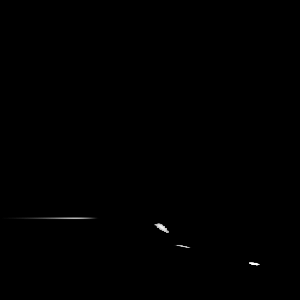

Supplement: S6 Data — The data is saved as *.png files. The subfolder entitled ‘0’ represent the undifferentiated cells while ‘1’ represent the differentiated MSCs. Every dataset also includes the original data, that were not affected by the augmentation algorithm (refer to tag ‘_Original.png’). (ZIP) [file pcbi.1010842.s025.zip › S6 Data/MSCs/MSC_AugIter-40_10Stretch_10Shift/0/MSC-1_25.png]

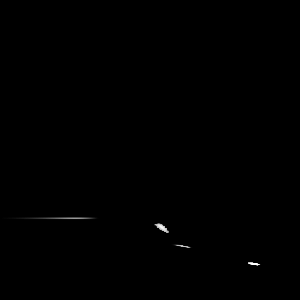

Supplement: S6 Data — The data is saved as *.png files. The subfolder entitled ‘0’ represent the undifferentiated cells while ‘1’ represent the differentiated MSCs. Every dataset also includes the original data, that were not affected by the augmentation algorithm (refer to tag ‘_Original.png’). (ZIP) [file pcbi.1010842.s025.zip › S6 Data/MSCs/MSC_AugIter-40_10Stretch_10Shift/0/MSC-1_26.png]

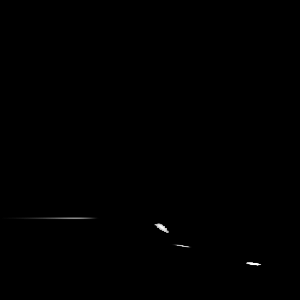

Supplement: S6 Data — The data is saved as *.png files. The subfolder entitled ‘0’ represent the undifferentiated cells while ‘1’ represent the differentiated MSCs. Every dataset also includes the original data, that were not affected by the augmentation algorithm (refer to tag ‘_Original.png’). (ZIP) [file pcbi.1010842.s025.zip › S6 Data/MSCs/MSC_AugIter-40_10Stretch_10Shift/0/MSC-1_27.png]

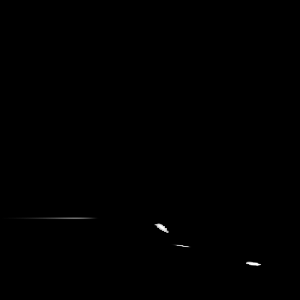

Supplement: S6 Data — The data is saved as *.png files. The subfolder entitled ‘0’ represent the undifferentiated cells while ‘1’ represent the differentiated MSCs. Every dataset also includes the original data, that were not affected by the augmentation algorithm (refer to tag ‘_Original.png’). (ZIP) [file pcbi.1010842.s025.zip › S6 Data/MSCs/MSC_AugIter-40_10Stretch_10Shift/0/MSC-1_28.png]

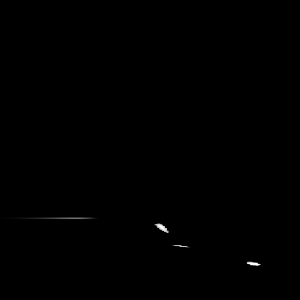

Supplement: S6 Data — The data is saved as *.png files. The subfolder entitled ‘0’ represent the undifferentiated cells while ‘1’ represent the differentiated MSCs. Every dataset also includes the original data, that were not affected by the augmentation algorithm (refer to tag ‘_Original.png’). (ZIP) [file pcbi.1010842.s025.zip › S6 Data/MSCs/MSC_AugIter-40_10Stretch_10Shift/0/MSC-1_29.png]

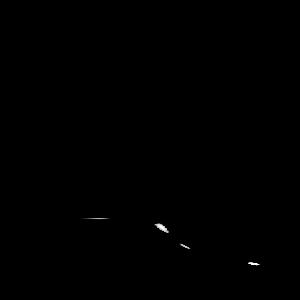

Supplement: S6 Data — The data is saved as *.png files. The subfolder entitled ‘0’ represent the undifferentiated cells while ‘1’ represent the differentiated MSCs. Every dataset also includes the original data, that were not affected by the augmentation algorithm (refer to tag ‘_Original.png’). (ZIP) [file pcbi.1010842.s025.zip › S6 Data/MSCs/MSC_AugIter-40_10Stretch_10Shift/0/MSC-1_3.png]

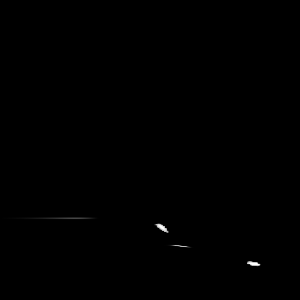

Supplement: S6 Data — The data is saved as *.png files. The subfolder entitled ‘0’ represent the undifferentiated cells while ‘1’ represent the differentiated MSCs. Every dataset also includes the original data, that were not affected by the augmentation algorithm (refer to tag ‘_Original.png’). (ZIP) [file pcbi.1010842.s025.zip › S6 Data/MSCs/MSC_AugIter-40_10Stretch_10Shift/0/MSC-1_30.png]

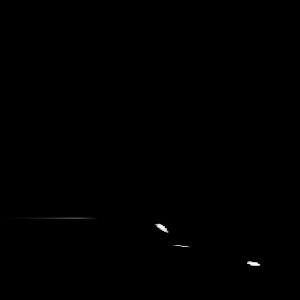

Supplement: S6 Data — The data is saved as *.png files. The subfolder entitled ‘0’ represent the undifferentiated cells while ‘1’ represent the differentiated MSCs. Every dataset also includes the original data, that were not affected by the augmentation algorithm (refer to tag ‘_Original.png’). (ZIP) [file pcbi.1010842.s025.zip › S6 Data/MSCs/MSC_AugIter-40_10Stretch_10Shift/0/MSC-1_31.png]

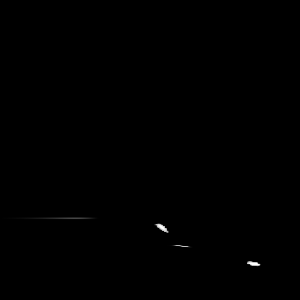

Supplement: S6 Data — The data is saved as *.png files. The subfolder entitled ‘0’ represent the undifferentiated cells while ‘1’ represent the differentiated MSCs. Every dataset also includes the original data, that were not affected by the augmentation algorithm (refer to tag ‘_Original.png’). (ZIP) [file pcbi.1010842.s025.zip › S6 Data/MSCs/MSC_AugIter-40_10Stretch_10Shift/0/MSC-1_32.png]

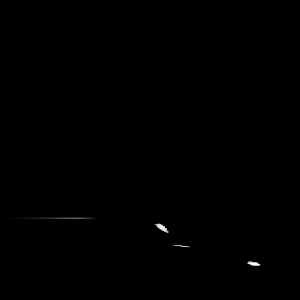

Supplement: S6 Data — The data is saved as *.png files. The subfolder entitled ‘0’ represent the undifferentiated cells while ‘1’ represent the differentiated MSCs. Every dataset also includes the original data, that were not affected by the augmentation algorithm (refer to tag ‘_Original.png’). (ZIP) [file pcbi.1010842.s025.zip › S6 Data/MSCs/MSC_AugIter-40_10Stretch_10Shift/0/MSC-1_33.png]

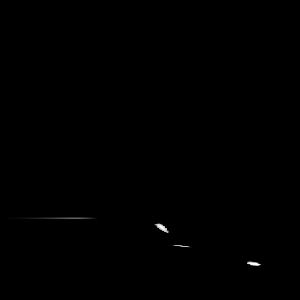

Supplement: S6 Data — The data is saved as *.png files. The subfolder entitled ‘0’ represent the undifferentiated cells while ‘1’ represent the differentiated MSCs. Every dataset also includes the original data, that were not affected by the augmentation algorithm (refer to tag ‘_Original.png’). (ZIP) [file pcbi.1010842.s025.zip › S6 Data/MSCs/MSC_AugIter-40_10Stretch_10Shift/0/MSC-1_34.png]

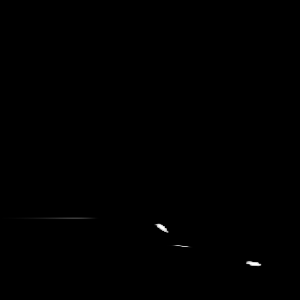

Supplement: S6 Data — The data is saved as *.png files. The subfolder entitled ‘0’ represent the undifferentiated cells while ‘1’ represent the differentiated MSCs. Every dataset also includes the original data, that were not affected by the augmentation algorithm (refer to tag ‘_Original.png’). (ZIP) [file pcbi.1010842.s025.zip › S6 Data/MSCs/MSC_AugIter-40_10Stretch_10Shift/0/MSC-1_35.png]

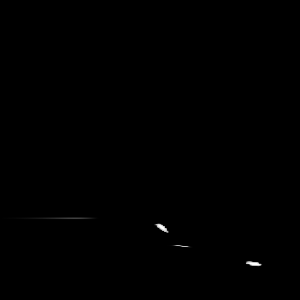

Supplement: S6 Data — The data is saved as *.png files. The subfolder entitled ‘0’ represent the undifferentiated cells while ‘1’ represent the differentiated MSCs. Every dataset also includes the original data, that were not affected by the augmentation algorithm (refer to tag ‘_Original.png’). (ZIP) [file pcbi.1010842.s025.zip › S6 Data/MSCs/MSC_AugIter-40_10Stretch_10Shift/0/MSC-1_36.png]

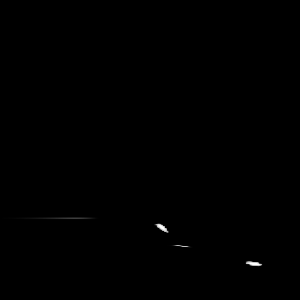

Supplement: S6 Data — The data is saved as *.png files. The subfolder entitled ‘0’ represent the undifferentiated cells while ‘1’ represent the differentiated MSCs. Every dataset also includes the original data, that were not affected by the augmentation algorithm (refer to tag ‘_Original.png’). (ZIP) [file pcbi.1010842.s025.zip › S6 Data/MSCs/MSC_AugIter-40_10Stretch_10Shift/0/MSC-1_37.png]

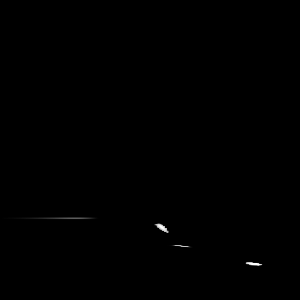

Supplement: S6 Data — The data is saved as *.png files. The subfolder entitled ‘0’ represent the undifferentiated cells while ‘1’ represent the differentiated MSCs. Every dataset also includes the original data, that were not affected by the augmentation algorithm (refer to tag ‘_Original.png’). (ZIP) [file pcbi.1010842.s025.zip › S6 Data/MSCs/MSC_AugIter-40_10Stretch_10Shift/0/MSC-1_38.png]

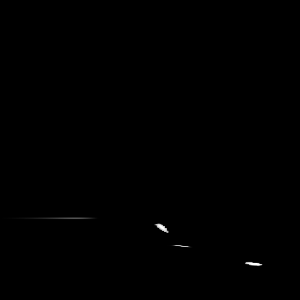

Supplement: S6 Data — The data is saved as *.png files. The subfolder entitled ‘0’ represent the undifferentiated cells while ‘1’ represent the differentiated MSCs. Every dataset also includes the original data, that were not affected by the augmentation algorithm (refer to tag ‘_Original.png’). (ZIP) [file pcbi.1010842.s025.zip › S6 Data/MSCs/MSC_AugIter-40_10Stretch_10Shift/0/MSC-1_39.png]

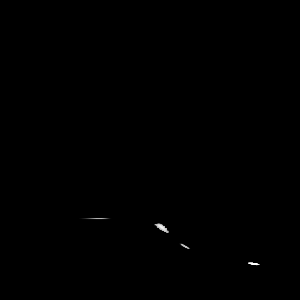

Supplement: S6 Data — The data is saved as *.png files. The subfolder entitled ‘0’ represent the undifferentiated cells while ‘1’ represent the differentiated MSCs. Every dataset also includes the original data, that were not affected by the augmentation algorithm (refer to tag ‘_Original.png’). (ZIP) [file pcbi.1010842.s025.zip › S6 Data/MSCs/MSC_AugIter-40_10Stretch_10Shift/0/MSC-1_4.png]

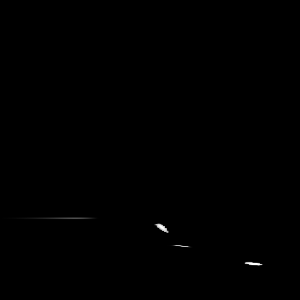

Supplement: S6 Data — The data is saved as *.png files. The subfolder entitled ‘0’ represent the undifferentiated cells while ‘1’ represent the differentiated MSCs. Every dataset also includes the original data, that were not affected by the augmentation algorithm (refer to tag ‘_Original.png’). (ZIP) [file pcbi.1010842.s025.zip › S6 Data/MSCs/MSC_AugIter-40_10Stretch_10Shift/0/MSC-1_40.png]

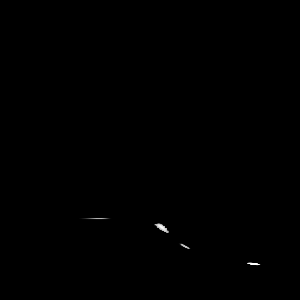

Supplement: S6 Data — The data is saved as *.png files. The subfolder entitled ‘0’ represent the undifferentiated cells while ‘1’ represent the differentiated MSCs. Every dataset also includes the original data, that were not affected by the augmentation algorithm (refer to tag ‘_Original.png’). (ZIP) [file pcbi.1010842.s025.zip › S6 Data/MSCs/MSC_AugIter-40_10Stretch_10Shift/0/MSC-1_5.png]

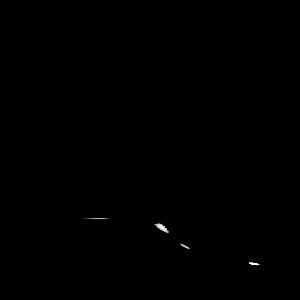

Supplement: S6 Data — The data is saved as *.png files. The subfolder entitled ‘0’ represent the undifferentiated cells while ‘1’ represent the differentiated MSCs. Every dataset also includes the original data, that were not affected by the augmentation algorithm (refer to tag ‘_Original.png’). (ZIP) [file pcbi.1010842.s025.zip › S6 Data/MSCs/MSC_AugIter-40_10Stretch_10Shift/0/MSC-1_6.png]

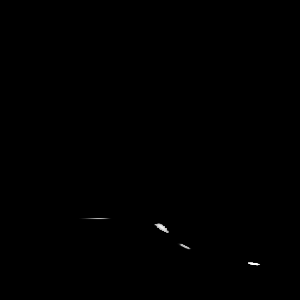

Supplement: S6 Data — The data is saved as *.png files. The subfolder entitled ‘0’ represent the undifferentiated cells while ‘1’ represent the differentiated MSCs. Every dataset also includes the original data, that were not affected by the augmentation algorithm (refer to tag ‘_Original.png’). (ZIP) [file pcbi.1010842.s025.zip › S6 Data/MSCs/MSC_AugIter-40_10Stretch_10Shift/0/MSC-1_7.png]

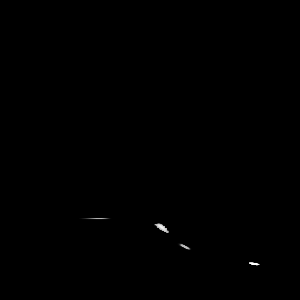

Supplement: S6 Data — The data is saved as *.png files. The subfolder entitled ‘0’ represent the undifferentiated cells while ‘1’ represent the differentiated MSCs. Every dataset also includes the original data, that were not affected by the augmentation algorithm (refer to tag ‘_Original.png’). (ZIP) [file pcbi.1010842.s025.zip › S6 Data/MSCs/MSC_AugIter-40_10Stretch_10Shift/0/MSC-1_8.png]

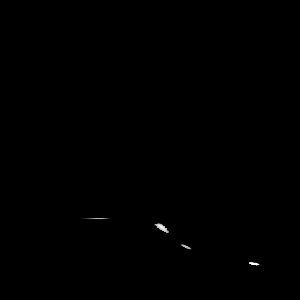

Supplement: S6 Data — The data is saved as *.png files. The subfolder entitled ‘0’ represent the undifferentiated cells while ‘1’ represent the differentiated MSCs. Every dataset also includes the original data, that were not affected by the augmentation algorithm (refer to tag ‘_Original.png’). (ZIP) [file pcbi.1010842.s025.zip › S6 Data/MSCs/MSC_AugIter-40_10Stretch_10Shift/0/MSC-1_9.png]

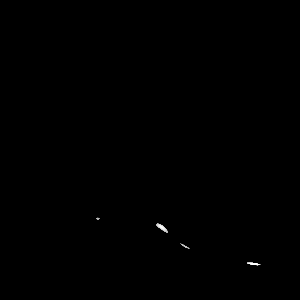

Supplement: S6 Data — The data is saved as *.png files. The subfolder entitled ‘0’ represent the undifferentiated cells while ‘1’ represent the differentiated MSCs. Every dataset also includes the original data, that were not affected by the augmentation algorithm (refer to tag ‘_Original.png’). (ZIP) [file pcbi.1010842.s025.zip › S6 Data/MSCs/MSC_AugIter-40_10Stretch_10Shift/0/MSC-1_Original.png]

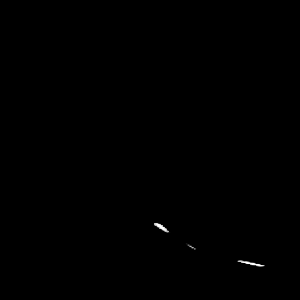

Supplement: S6 Data — The data is saved as *.png files. The subfolder entitled ‘0’ represent the undifferentiated cells while ‘1’ represent the differentiated MSCs. Every dataset also includes the original data, that were not affected by the augmentation algorithm (refer to tag ‘_Original.png’). (ZIP) [file pcbi.1010842.s025.zip › S6 Data/MSCs/MSC_AugIter-40_10Stretch_10Shift/0/MSC-10_1.png]

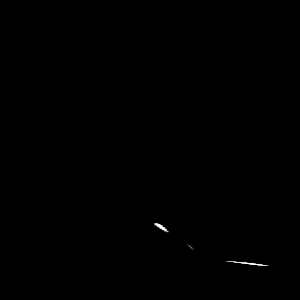

Supplement: S6 Data — The data is saved as *.png files. The subfolder entitled ‘0’ represent the undifferentiated cells while ‘1’ represent the differentiated MSCs. Every dataset also includes the original data, that were not affected by the augmentation algorithm (refer to tag ‘_Original.png’). (ZIP) [file pcbi.1010842.s025.zip › S6 Data/MSCs/MSC_AugIter-40_10Stretch_10Shift/0/MSC-10_10.png]

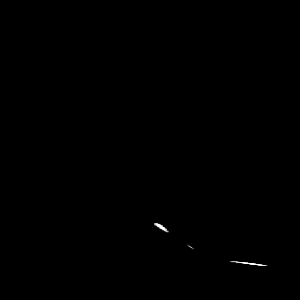

Supplement: S6 Data — The data is saved as *.png files. The subfolder entitled ‘0’ represent the undifferentiated cells while ‘1’ represent the differentiated MSCs. Every dataset also includes the original data, that were not affected by the augmentation algorithm (refer to tag ‘_Original.png’). (ZIP) [file pcbi.1010842.s025.zip › S6 Data/MSCs/MSC_AugIter-40_10Stretch_10Shift/0/MSC-10_11.png]

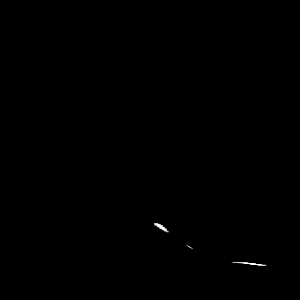

Supplement: S6 Data — The data is saved as *.png files. The subfolder entitled ‘0’ represent the undifferentiated cells while ‘1’ represent the differentiated MSCs. Every dataset also includes the original data, that were not affected by the augmentation algorithm (refer to tag ‘_Original.png’). (ZIP) [file pcbi.1010842.s025.zip › S6 Data/MSCs/MSC_AugIter-40_10Stretch_10Shift/0/MSC-10_12.png]

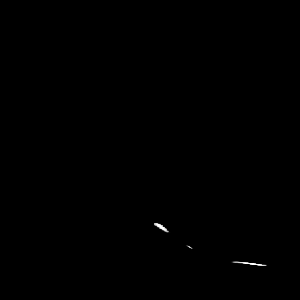

Supplement: S6 Data — The data is saved as *.png files. The subfolder entitled ‘0’ represent the undifferentiated cells while ‘1’ represent the differentiated MSCs. Every dataset also includes the original data, that were not affected by the augmentation algorithm (refer to tag ‘_Original.png’). (ZIP) [file pcbi.1010842.s025.zip › S6 Data/MSCs/MSC_AugIter-40_10Stretch_10Shift/0/MSC-10_13.png]

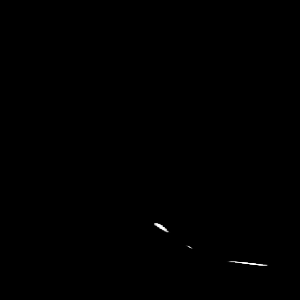

Supplement: S6 Data — The data is saved as *.png files. The subfolder entitled ‘0’ represent the undifferentiated cells while ‘1’ represent the differentiated MSCs. Every dataset also includes the original data, that were not affected by the augmentation algorithm (refer to tag ‘_Original.png’). (ZIP) [file pcbi.1010842.s025.zip › S6 Data/MSCs/MSC_AugIter-40_10Stretch_10Shift/0/MSC-10_14.png]

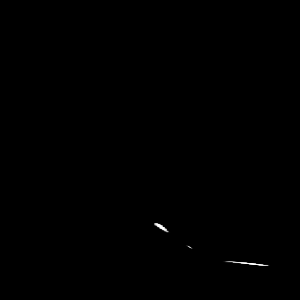

Supplement: S6 Data — The data is saved as *.png files. The subfolder entitled ‘0’ represent the undifferentiated cells while ‘1’ represent the differentiated MSCs. Every dataset also includes the original data, that were not affected by the augmentation algorithm (refer to tag ‘_Original.png’). (ZIP) [file pcbi.1010842.s025.zip › S6 Data/MSCs/MSC_AugIter-40_10Stretch_10Shift/0/MSC-10_15.png]

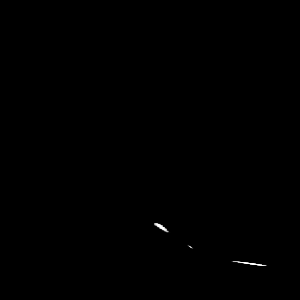

Supplement: S6 Data — The data is saved as *.png files. The subfolder entitled ‘0’ represent the undifferentiated cells while ‘1’ represent the differentiated MSCs. Every dataset also includes the original data, that were not affected by the augmentation algorithm (refer to tag ‘_Original.png’). (ZIP) [file pcbi.1010842.s025.zip › S6 Data/MSCs/MSC_AugIter-40_10Stretch_10Shift/0/MSC-10_16.png]

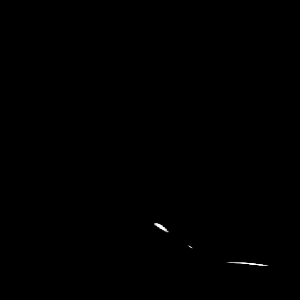

Supplement: S6 Data — The data is saved as *.png files. The subfolder entitled ‘0’ represent the undifferentiated cells while ‘1’ represent the differentiated MSCs. Every dataset also includes the original data, that were not affected by the augmentation algorithm (refer to tag ‘_Original.png’). (ZIP) [file pcbi.1010842.s025.zip › S6 Data/MSCs/MSC_AugIter-40_10Stretch_10Shift/0/MSC-10_17.png]

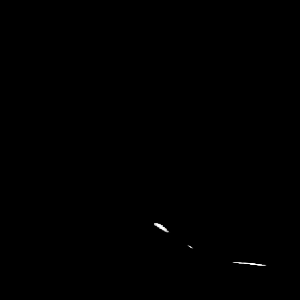

Supplement: S6 Data — The data is saved as *.png files. The subfolder entitled ‘0’ represent the undifferentiated cells while ‘1’ represent the differentiated MSCs. Every dataset also includes the original data, that were not affected by the augmentation algorithm (refer to tag ‘_Original.png’). (ZIP) [file pcbi.1010842.s025.zip › S6 Data/MSCs/MSC_AugIter-40_10Stretch_10Shift/0/MSC-10_18.png]

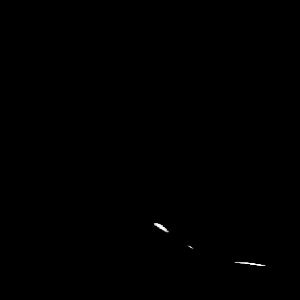

Supplement: S6 Data — The data is saved as *.png files. The subfolder entitled ‘0’ represent the undifferentiated cells while ‘1’ represent the differentiated MSCs. Every dataset also includes the original data, that were not affected by the augmentation algorithm (refer to tag ‘_Original.png’). (ZIP) [file pcbi.1010842.s025.zip › S6 Data/MSCs/MSC_AugIter-40_10Stretch_10Shift/0/MSC-10_19.png]

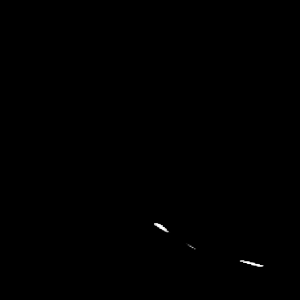

Supplement: S6 Data — The data is saved as *.png files. The subfolder entitled ‘0’ represent the undifferentiated cells while ‘1’ represent the differentiated MSCs. Every dataset also includes the original data, that were not affected by the augmentation algorithm (refer to tag ‘_Original.png’). (ZIP) [file pcbi.1010842.s025.zip › S6 Data/MSCs/MSC_AugIter-40_10Stretch_10Shift/0/MSC-10_2.png]

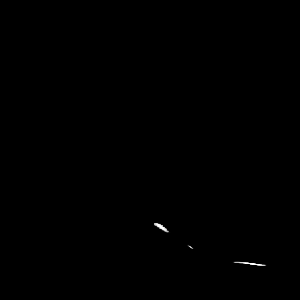

Supplement: S6 Data — The data is saved as *.png files. The subfolder entitled ‘0’ represent the undifferentiated cells while ‘1’ represent the differentiated MSCs. Every dataset also includes the original data, that were not affected by the augmentation algorithm (refer to tag ‘_Original.png’). (ZIP) [file pcbi.1010842.s025.zip › S6 Data/MSCs/MSC_AugIter-40_10Stretch_10Shift/0/MSC-10_20.png]

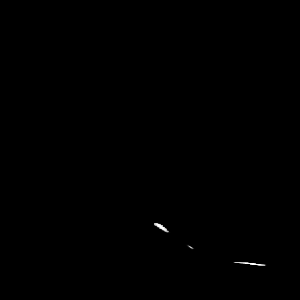

Supplement: S6 Data — The data is saved as *.png files. The subfolder entitled ‘0’ represent the undifferentiated cells while ‘1’ represent the differentiated MSCs. Every dataset also includes the original data, that were not affected by the augmentation algorithm (refer to tag ‘_Original.png’). (ZIP) [file pcbi.1010842.s025.zip › S6 Data/MSCs/MSC_AugIter-40_10Stretch_10Shift/0/MSC-10_21.png]

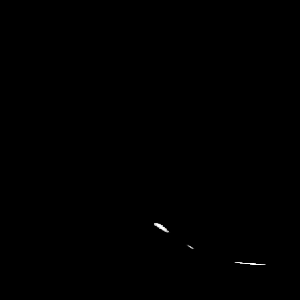

Supplement: S6 Data — The data is saved as *.png files. The subfolder entitled ‘0’ represent the undifferentiated cells while ‘1’ represent the differentiated MSCs. Every dataset also includes the original data, that were not affected by the augmentation algorithm (refer to tag ‘_Original.png’). (ZIP) [file pcbi.1010842.s025.zip › S6 Data/MSCs/MSC_AugIter-40_10Stretch_10Shift/0/MSC-10_22.png]

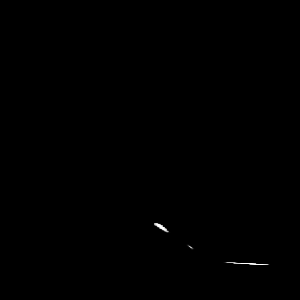

Supplement: S6 Data — The data is saved as *.png files. The subfolder entitled ‘0’ represent the undifferentiated cells while ‘1’ represent the differentiated MSCs. Every dataset also includes the original data, that were not affected by the augmentation algorithm (refer to tag ‘_Original.png’). (ZIP) [file pcbi.1010842.s025.zip › S6 Data/MSCs/MSC_AugIter-40_10Stretch_10Shift/0/MSC-10_23.png]

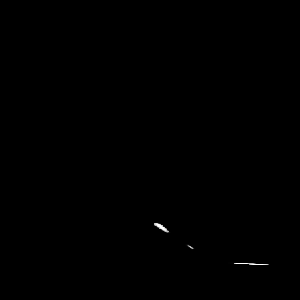

Supplement: S6 Data — The data is saved as *.png files. The subfolder entitled ‘0’ represent the undifferentiated cells while ‘1’ represent the differentiated MSCs. Every dataset also includes the original data, that were not affected by the augmentation algorithm (refer to tag ‘_Original.png’). (ZIP) [file pcbi.1010842.s025.zip › S6 Data/MSCs/MSC_AugIter-40_10Stretch_10Shift/0/MSC-10_24.png]

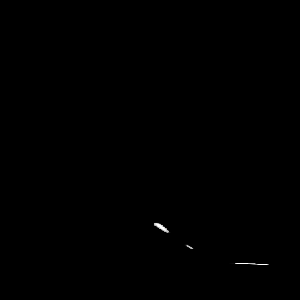

Supplement: S6 Data — The data is saved as *.png files. The subfolder entitled ‘0’ represent the undifferentiated cells while ‘1’ represent the differentiated MSCs. Every dataset also includes the original data, that were not affected by the augmentation algorithm (refer to tag ‘_Original.png’). (ZIP) [file pcbi.1010842.s025.zip › S6 Data/MSCs/MSC_AugIter-40_10Stretch_10Shift/0/MSC-10_25.png]

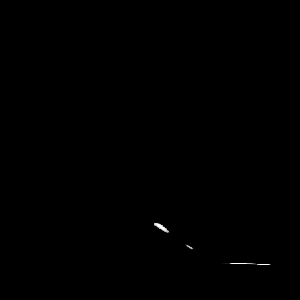

Supplement: S6 Data — The data is saved as *.png files. The subfolder entitled ‘0’ represent the undifferentiated cells while ‘1’ represent the differentiated MSCs. Every dataset also includes the original data, that were not affected by the augmentation algorithm (refer to tag ‘_Original.png’). (ZIP) [file pcbi.1010842.s025.zip › S6 Data/MSCs/MSC_AugIter-40_10Stretch_10Shift/0/MSC-10_26.png]

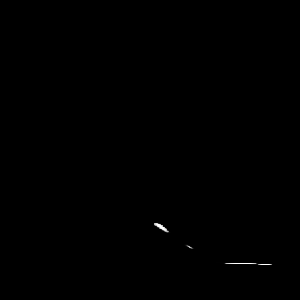

Supplement: S6 Data — The data is saved as *.png files. The subfolder entitled ‘0’ represent the undifferentiated cells while ‘1’ represent the differentiated MSCs. Every dataset also includes the original data, that were not affected by the augmentation algorithm (refer to tag ‘_Original.png’). (ZIP) [file pcbi.1010842.s025.zip › S6 Data/MSCs/MSC_AugIter-40_10Stretch_10Shift/0/MSC-10_27.png]

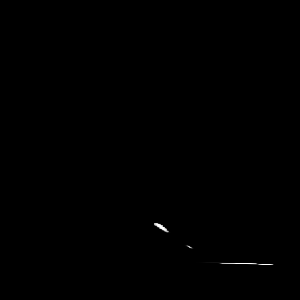

Supplement: S6 Data — The data is saved as *.png files. The subfolder entitled ‘0’ represent the undifferentiated cells while ‘1’ represent the differentiated MSCs. Every dataset also includes the original data, that were not affected by the augmentation algorithm (refer to tag ‘_Original.png’). (ZIP) [file pcbi.1010842.s025.zip › S6 Data/MSCs/MSC_AugIter-40_10Stretch_10Shift/0/MSC-10_28.png]

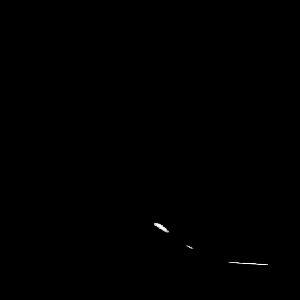

Supplement: S6 Data — The data is saved as *.png files. The subfolder entitled ‘0’ represent the undifferentiated cells while ‘1’ represent the differentiated MSCs. Every dataset also includes the original data, that were not affected by the augmentation algorithm (refer to tag ‘_Original.png’). (ZIP) [file pcbi.1010842.s025.zip › S6 Data/MSCs/MSC_AugIter-40_10Stretch_10Shift/0/MSC-10_29.png]

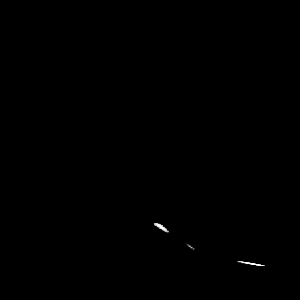

Supplement: S6 Data — The data is saved as *.png files. The subfolder entitled ‘0’ represent the undifferentiated cells while ‘1’ represent the differentiated MSCs. Every dataset also includes the original data, that were not affected by the augmentation algorithm (refer to tag ‘_Original.png’). (ZIP) [file pcbi.1010842.s025.zip › S6 Data/MSCs/MSC_AugIter-40_10Stretch_10Shift/0/MSC-10_3.png]

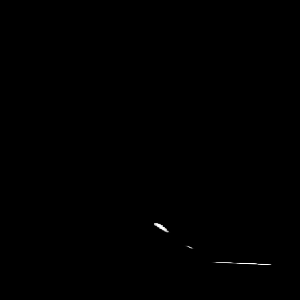

Supplement: S6 Data — The data is saved as *.png files. The subfolder entitled ‘0’ represent the undifferentiated cells while ‘1’ represent the differentiated MSCs. Every dataset also includes the original data, that were not affected by the augmentation algorithm (refer to tag ‘_Original.png’). (ZIP) [file pcbi.1010842.s025.zip › S6 Data/MSCs/MSC_AugIter-40_10Stretch_10Shift/0/MSC-10_30.png]

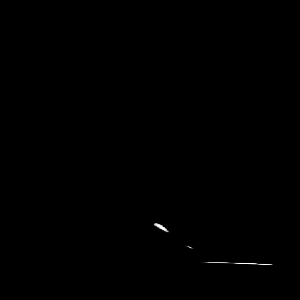

Supplement: S6 Data — The data is saved as *.png files. The subfolder entitled ‘0’ represent the undifferentiated cells while ‘1’ represent the differentiated MSCs. Every dataset also includes the original data, that were not affected by the augmentation algorithm (refer to tag ‘_Original.png’). (ZIP) [file pcbi.1010842.s025.zip › S6 Data/MSCs/MSC_AugIter-40_10Stretch_10Shift/0/MSC-10_31.png]

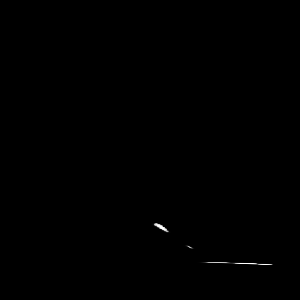

Supplement: S6 Data — The data is saved as *.png files. The subfolder entitled ‘0’ represent the undifferentiated cells while ‘1’ represent the differentiated MSCs. Every dataset also includes the original data, that were not affected by the augmentation algorithm (refer to tag ‘_Original.png’). (ZIP) [file pcbi.1010842.s025.zip › S6 Data/MSCs/MSC_AugIter-40_10Stretch_10Shift/0/MSC-10_32.png]

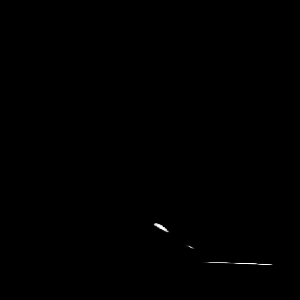

Supplement: S6 Data — The data is saved as *.png files. The subfolder entitled ‘0’ represent the undifferentiated cells while ‘1’ represent the differentiated MSCs. Every dataset also includes the original data, that were not affected by the augmentation algorithm (refer to tag ‘_Original.png’). (ZIP) [file pcbi.1010842.s025.zip › S6 Data/MSCs/MSC_AugIter-40_10Stretch_10Shift/0/MSC-10_33.png]

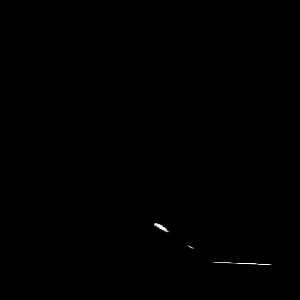

Supplement: S6 Data — The data is saved as *.png files. The subfolder entitled ‘0’ represent the undifferentiated cells while ‘1’ represent the differentiated MSCs. Every dataset also includes the original data, that were not affected by the augmentation algorithm (refer to tag ‘_Original.png’). (ZIP) [file pcbi.1010842.s025.zip › S6 Data/MSCs/MSC_AugIter-40_10Stretch_10Shift/0/MSC-10_34.png]
