# Supplementary figures and images for: Non-destructive classification of unlabeled cells: Combining an automated benchtop magnetic resonance scanner and artificial intelligence (part 2 of 3)
Source: PLoS Comput Biol. 2023 Feb 21;19(2):e1010842. doi: 10.1371/journal.pcbi.1010842 (PMC9983908; doi:10.1371/journal.pcbi.1010842)

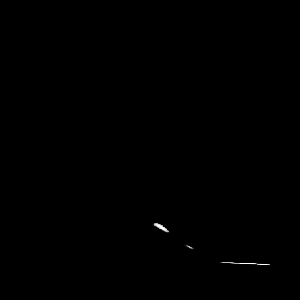

Supplement: S6 Data — The data is saved as *.png files. The subfolder entitled ‘0’ represent the undifferentiated cells while ‘1’ represent the differentiated MSCs. Every dataset also includes the original data, that were not affected by the augmentation algorithm (refer to tag ‘_Original.png’). (ZIP) [file pcbi.1010842.s025.zip › S6 Data/MSCs/MSC_AugIter-40_10Stretch_10Shift/0/MSC-10_35.png]

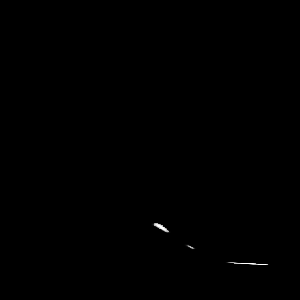

Supplement: S6 Data — The data is saved as *.png files. The subfolder entitled ‘0’ represent the undifferentiated cells while ‘1’ represent the differentiated MSCs. Every dataset also includes the original data, that were not affected by the augmentation algorithm (refer to tag ‘_Original.png’). (ZIP) [file pcbi.1010842.s025.zip › S6 Data/MSCs/MSC_AugIter-40_10Stretch_10Shift/0/MSC-10_36.png]

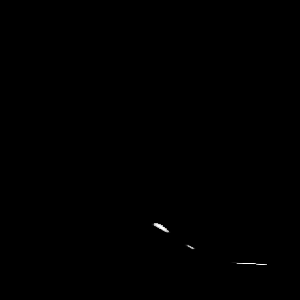

Supplement: S6 Data — The data is saved as *.png files. The subfolder entitled ‘0’ represent the undifferentiated cells while ‘1’ represent the differentiated MSCs. Every dataset also includes the original data, that were not affected by the augmentation algorithm (refer to tag ‘_Original.png’). (ZIP) [file pcbi.1010842.s025.zip › S6 Data/MSCs/MSC_AugIter-40_10Stretch_10Shift/0/MSC-10_37.png]

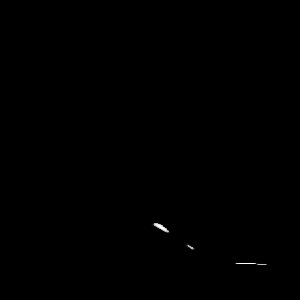

Supplement: S6 Data — The data is saved as *.png files. The subfolder entitled ‘0’ represent the undifferentiated cells while ‘1’ represent the differentiated MSCs. Every dataset also includes the original data, that were not affected by the augmentation algorithm (refer to tag ‘_Original.png’). (ZIP) [file pcbi.1010842.s025.zip › S6 Data/MSCs/MSC_AugIter-40_10Stretch_10Shift/0/MSC-10_38.png]

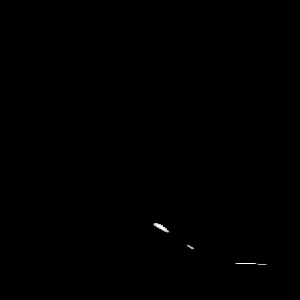

Supplement: S6 Data — The data is saved as *.png files. The subfolder entitled ‘0’ represent the undifferentiated cells while ‘1’ represent the differentiated MSCs. Every dataset also includes the original data, that were not affected by the augmentation algorithm (refer to tag ‘_Original.png’). (ZIP) [file pcbi.1010842.s025.zip › S6 Data/MSCs/MSC_AugIter-40_10Stretch_10Shift/0/MSC-10_39.png]

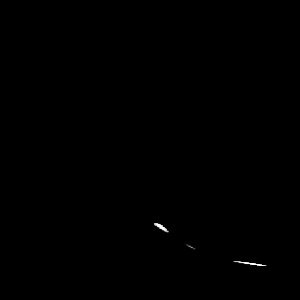

Supplement: S6 Data — The data is saved as *.png files. The subfolder entitled ‘0’ represent the undifferentiated cells while ‘1’ represent the differentiated MSCs. Every dataset also includes the original data, that were not affected by the augmentation algorithm (refer to tag ‘_Original.png’). (ZIP) [file pcbi.1010842.s025.zip › S6 Data/MSCs/MSC_AugIter-40_10Stretch_10Shift/0/MSC-10_4.png]

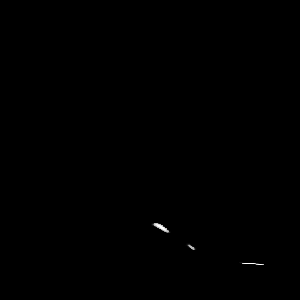

Supplement: S6 Data — The data is saved as *.png files. The subfolder entitled ‘0’ represent the undifferentiated cells while ‘1’ represent the differentiated MSCs. Every dataset also includes the original data, that were not affected by the augmentation algorithm (refer to tag ‘_Original.png’). (ZIP) [file pcbi.1010842.s025.zip › S6 Data/MSCs/MSC_AugIter-40_10Stretch_10Shift/0/MSC-10_40.png]

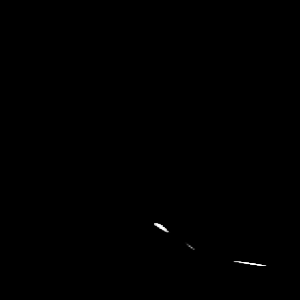

Supplement: S6 Data — The data is saved as *.png files. The subfolder entitled ‘0’ represent the undifferentiated cells while ‘1’ represent the differentiated MSCs. Every dataset also includes the original data, that were not affected by the augmentation algorithm (refer to tag ‘_Original.png’). (ZIP) [file pcbi.1010842.s025.zip › S6 Data/MSCs/MSC_AugIter-40_10Stretch_10Shift/0/MSC-10_5.png]

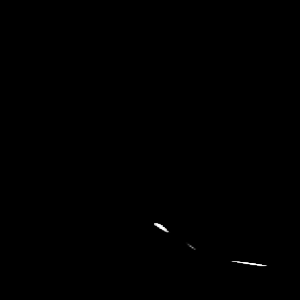

Supplement: S6 Data — The data is saved as *.png files. The subfolder entitled ‘0’ represent the undifferentiated cells while ‘1’ represent the differentiated MSCs. Every dataset also includes the original data, that were not affected by the augmentation algorithm (refer to tag ‘_Original.png’). (ZIP) [file pcbi.1010842.s025.zip › S6 Data/MSCs/MSC_AugIter-40_10Stretch_10Shift/0/MSC-10_6.png]

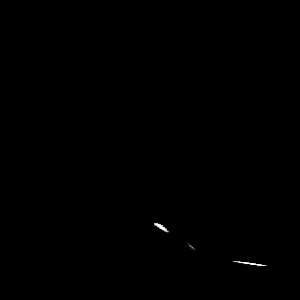

Supplement: S6 Data — The data is saved as *.png files. The subfolder entitled ‘0’ represent the undifferentiated cells while ‘1’ represent the differentiated MSCs. Every dataset also includes the original data, that were not affected by the augmentation algorithm (refer to tag ‘_Original.png’). (ZIP) [file pcbi.1010842.s025.zip › S6 Data/MSCs/MSC_AugIter-40_10Stretch_10Shift/0/MSC-10_7.png]

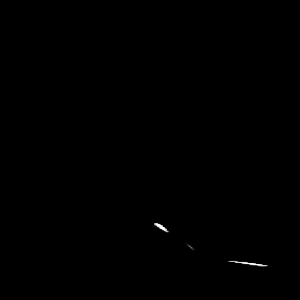

Supplement: S6 Data — The data is saved as *.png files. The subfolder entitled ‘0’ represent the undifferentiated cells while ‘1’ represent the differentiated MSCs. Every dataset also includes the original data, that were not affected by the augmentation algorithm (refer to tag ‘_Original.png’). (ZIP) [file pcbi.1010842.s025.zip › S6 Data/MSCs/MSC_AugIter-40_10Stretch_10Shift/0/MSC-10_8.png]

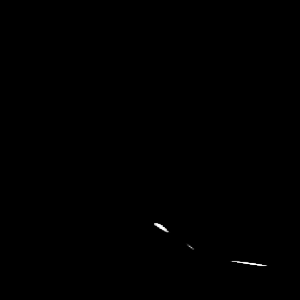

Supplement: S6 Data — The data is saved as *.png files. The subfolder entitled ‘0’ represent the undifferentiated cells while ‘1’ represent the differentiated MSCs. Every dataset also includes the original data, that were not affected by the augmentation algorithm (refer to tag ‘_Original.png’). (ZIP) [file pcbi.1010842.s025.zip › S6 Data/MSCs/MSC_AugIter-40_10Stretch_10Shift/0/MSC-10_9.png]

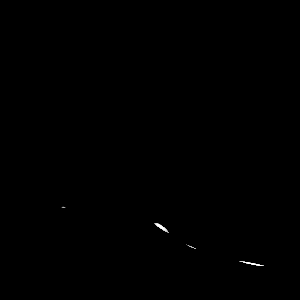

Supplement: S6 Data — The data is saved as *.png files. The subfolder entitled ‘0’ represent the undifferentiated cells while ‘1’ represent the differentiated MSCs. Every dataset also includes the original data, that were not affected by the augmentation algorithm (refer to tag ‘_Original.png’). (ZIP) [file pcbi.1010842.s025.zip › S6 Data/MSCs/MSC_AugIter-40_10Stretch_10Shift/0/MSC-10_Original.png]

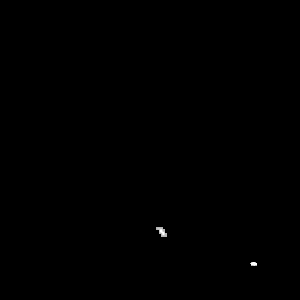

Supplement: S6 Data — The data is saved as *.png files. The subfolder entitled ‘0’ represent the undifferentiated cells while ‘1’ represent the differentiated MSCs. Every dataset also includes the original data, that were not affected by the augmentation algorithm (refer to tag ‘_Original.png’). (ZIP) [file pcbi.1010842.s025.zip › S6 Data/MSCs/MSC_AugIter-40_10Stretch_10Shift/0/MSC-2_1.png]

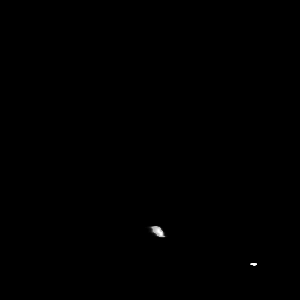

Supplement: S6 Data — The data is saved as *.png files. The subfolder entitled ‘0’ represent the undifferentiated cells while ‘1’ represent the differentiated MSCs. Every dataset also includes the original data, that were not affected by the augmentation algorithm (refer to tag ‘_Original.png’). (ZIP) [file pcbi.1010842.s025.zip › S6 Data/MSCs/MSC_AugIter-40_10Stretch_10Shift/0/MSC-2_10.png]

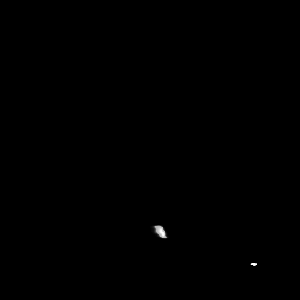

Supplement: S6 Data — The data is saved as *.png files. The subfolder entitled ‘0’ represent the undifferentiated cells while ‘1’ represent the differentiated MSCs. Every dataset also includes the original data, that were not affected by the augmentation algorithm (refer to tag ‘_Original.png’). (ZIP) [file pcbi.1010842.s025.zip › S6 Data/MSCs/MSC_AugIter-40_10Stretch_10Shift/0/MSC-2_11.png]

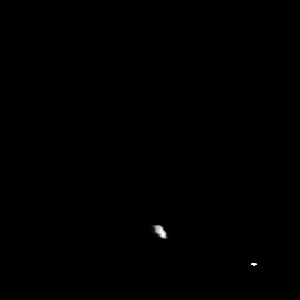

Supplement: S6 Data — The data is saved as *.png files. The subfolder entitled ‘0’ represent the undifferentiated cells while ‘1’ represent the differentiated MSCs. Every dataset also includes the original data, that were not affected by the augmentation algorithm (refer to tag ‘_Original.png’). (ZIP) [file pcbi.1010842.s025.zip › S6 Data/MSCs/MSC_AugIter-40_10Stretch_10Shift/0/MSC-2_12.png]

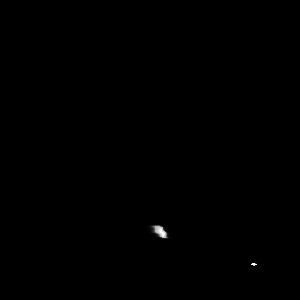

Supplement: S6 Data — The data is saved as *.png files. The subfolder entitled ‘0’ represent the undifferentiated cells while ‘1’ represent the differentiated MSCs. Every dataset also includes the original data, that were not affected by the augmentation algorithm (refer to tag ‘_Original.png’). (ZIP) [file pcbi.1010842.s025.zip › S6 Data/MSCs/MSC_AugIter-40_10Stretch_10Shift/0/MSC-2_13.png]

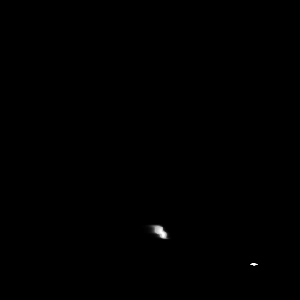

Supplement: S6 Data — The data is saved as *.png files. The subfolder entitled ‘0’ represent the undifferentiated cells while ‘1’ represent the differentiated MSCs. Every dataset also includes the original data, that were not affected by the augmentation algorithm (refer to tag ‘_Original.png’). (ZIP) [file pcbi.1010842.s025.zip › S6 Data/MSCs/MSC_AugIter-40_10Stretch_10Shift/0/MSC-2_14.png]

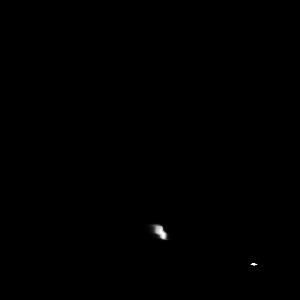

Supplement: S6 Data — The data is saved as *.png files. The subfolder entitled ‘0’ represent the undifferentiated cells while ‘1’ represent the differentiated MSCs. Every dataset also includes the original data, that were not affected by the augmentation algorithm (refer to tag ‘_Original.png’). (ZIP) [file pcbi.1010842.s025.zip › S6 Data/MSCs/MSC_AugIter-40_10Stretch_10Shift/0/MSC-2_15.png]

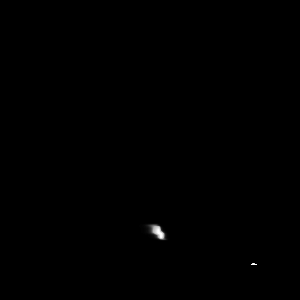

Supplement: S6 Data — The data is saved as *.png files. The subfolder entitled ‘0’ represent the undifferentiated cells while ‘1’ represent the differentiated MSCs. Every dataset also includes the original data, that were not affected by the augmentation algorithm (refer to tag ‘_Original.png’). (ZIP) [file pcbi.1010842.s025.zip › S6 Data/MSCs/MSC_AugIter-40_10Stretch_10Shift/0/MSC-2_16.png]

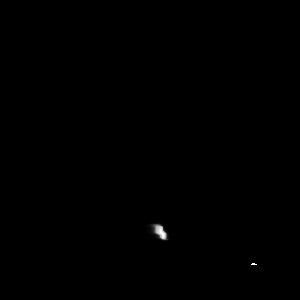

Supplement: S6 Data — The data is saved as *.png files. The subfolder entitled ‘0’ represent the undifferentiated cells while ‘1’ represent the differentiated MSCs. Every dataset also includes the original data, that were not affected by the augmentation algorithm (refer to tag ‘_Original.png’). (ZIP) [file pcbi.1010842.s025.zip › S6 Data/MSCs/MSC_AugIter-40_10Stretch_10Shift/0/MSC-2_17.png]

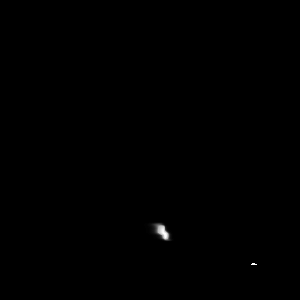

Supplement: S6 Data — The data is saved as *.png files. The subfolder entitled ‘0’ represent the undifferentiated cells while ‘1’ represent the differentiated MSCs. Every dataset also includes the original data, that were not affected by the augmentation algorithm (refer to tag ‘_Original.png’). (ZIP) [file pcbi.1010842.s025.zip › S6 Data/MSCs/MSC_AugIter-40_10Stretch_10Shift/0/MSC-2_18.png]

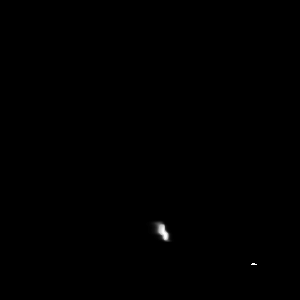

Supplement: S6 Data — The data is saved as *.png files. The subfolder entitled ‘0’ represent the undifferentiated cells while ‘1’ represent the differentiated MSCs. Every dataset also includes the original data, that were not affected by the augmentation algorithm (refer to tag ‘_Original.png’). (ZIP) [file pcbi.1010842.s025.zip › S6 Data/MSCs/MSC_AugIter-40_10Stretch_10Shift/0/MSC-2_19.png]

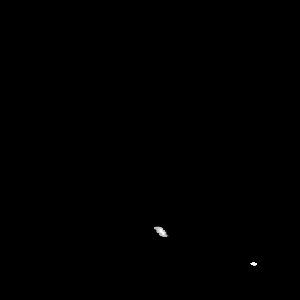

Supplement: S6 Data — The data is saved as *.png files. The subfolder entitled ‘0’ represent the undifferentiated cells while ‘1’ represent the differentiated MSCs. Every dataset also includes the original data, that were not affected by the augmentation algorithm (refer to tag ‘_Original.png’). (ZIP) [file pcbi.1010842.s025.zip › S6 Data/MSCs/MSC_AugIter-40_10Stretch_10Shift/0/MSC-2_2.png]

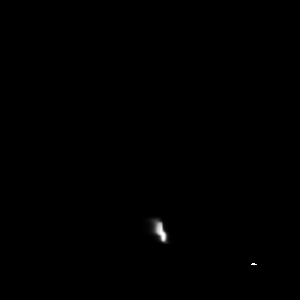

Supplement: S6 Data — The data is saved as *.png files. The subfolder entitled ‘0’ represent the undifferentiated cells while ‘1’ represent the differentiated MSCs. Every dataset also includes the original data, that were not affected by the augmentation algorithm (refer to tag ‘_Original.png’). (ZIP) [file pcbi.1010842.s025.zip › S6 Data/MSCs/MSC_AugIter-40_10Stretch_10Shift/0/MSC-2_20.png]

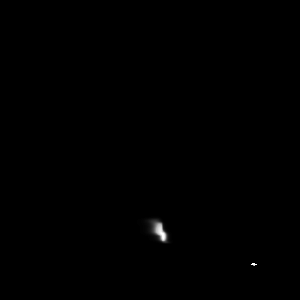

Supplement: S6 Data — The data is saved as *.png files. The subfolder entitled ‘0’ represent the undifferentiated cells while ‘1’ represent the differentiated MSCs. Every dataset also includes the original data, that were not affected by the augmentation algorithm (refer to tag ‘_Original.png’). (ZIP) [file pcbi.1010842.s025.zip › S6 Data/MSCs/MSC_AugIter-40_10Stretch_10Shift/0/MSC-2_21.png]

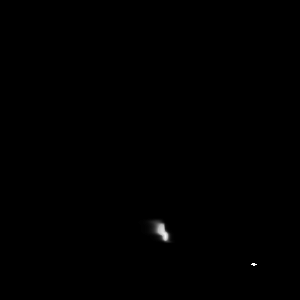

Supplement: S6 Data — The data is saved as *.png files. The subfolder entitled ‘0’ represent the undifferentiated cells while ‘1’ represent the differentiated MSCs. Every dataset also includes the original data, that were not affected by the augmentation algorithm (refer to tag ‘_Original.png’). (ZIP) [file pcbi.1010842.s025.zip › S6 Data/MSCs/MSC_AugIter-40_10Stretch_10Shift/0/MSC-2_22.png]

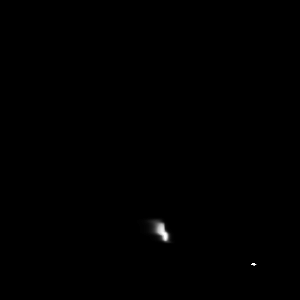

Supplement: S6 Data — The data is saved as *.png files. The subfolder entitled ‘0’ represent the undifferentiated cells while ‘1’ represent the differentiated MSCs. Every dataset also includes the original data, that were not affected by the augmentation algorithm (refer to tag ‘_Original.png’). (ZIP) [file pcbi.1010842.s025.zip › S6 Data/MSCs/MSC_AugIter-40_10Stretch_10Shift/0/MSC-2_23.png]

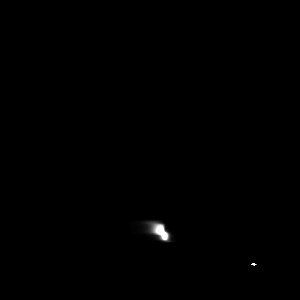

Supplement: S6 Data — The data is saved as *.png files. The subfolder entitled ‘0’ represent the undifferentiated cells while ‘1’ represent the differentiated MSCs. Every dataset also includes the original data, that were not affected by the augmentation algorithm (refer to tag ‘_Original.png’). (ZIP) [file pcbi.1010842.s025.zip › S6 Data/MSCs/MSC_AugIter-40_10Stretch_10Shift/0/MSC-2_24.png]

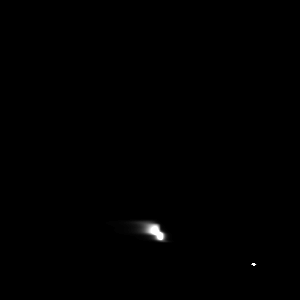

Supplement: S6 Data — The data is saved as *.png files. The subfolder entitled ‘0’ represent the undifferentiated cells while ‘1’ represent the differentiated MSCs. Every dataset also includes the original data, that were not affected by the augmentation algorithm (refer to tag ‘_Original.png’). (ZIP) [file pcbi.1010842.s025.zip › S6 Data/MSCs/MSC_AugIter-40_10Stretch_10Shift/0/MSC-2_25.png]

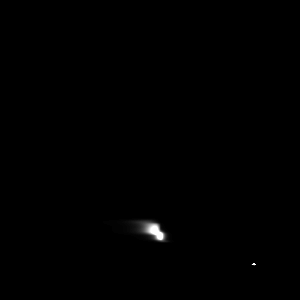

Supplement: S6 Data — The data is saved as *.png files. The subfolder entitled ‘0’ represent the undifferentiated cells while ‘1’ represent the differentiated MSCs. Every dataset also includes the original data, that were not affected by the augmentation algorithm (refer to tag ‘_Original.png’). (ZIP) [file pcbi.1010842.s025.zip › S6 Data/MSCs/MSC_AugIter-40_10Stretch_10Shift/0/MSC-2_26.png]

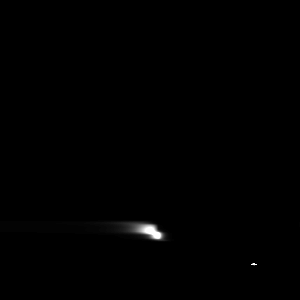

Supplement: S6 Data — The data is saved as *.png files. The subfolder entitled ‘0’ represent the undifferentiated cells while ‘1’ represent the differentiated MSCs. Every dataset also includes the original data, that were not affected by the augmentation algorithm (refer to tag ‘_Original.png’). (ZIP) [file pcbi.1010842.s025.zip › S6 Data/MSCs/MSC_AugIter-40_10Stretch_10Shift/0/MSC-2_27.png]

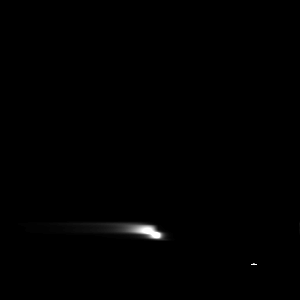

Supplement: S6 Data — The data is saved as *.png files. The subfolder entitled ‘0’ represent the undifferentiated cells while ‘1’ represent the differentiated MSCs. Every dataset also includes the original data, that were not affected by the augmentation algorithm (refer to tag ‘_Original.png’). (ZIP) [file pcbi.1010842.s025.zip › S6 Data/MSCs/MSC_AugIter-40_10Stretch_10Shift/0/MSC-2_28.png]

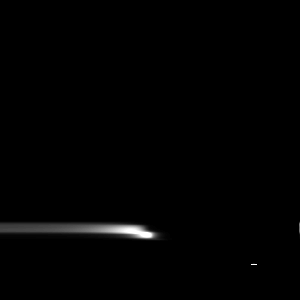

Supplement: S6 Data — The data is saved as *.png files. The subfolder entitled ‘0’ represent the undifferentiated cells while ‘1’ represent the differentiated MSCs. Every dataset also includes the original data, that were not affected by the augmentation algorithm (refer to tag ‘_Original.png’). (ZIP) [file pcbi.1010842.s025.zip › S6 Data/MSCs/MSC_AugIter-40_10Stretch_10Shift/0/MSC-2_29.png]

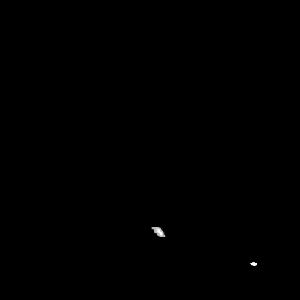

Supplement: S6 Data — The data is saved as *.png files. The subfolder entitled ‘0’ represent the undifferentiated cells while ‘1’ represent the differentiated MSCs. Every dataset also includes the original data, that were not affected by the augmentation algorithm (refer to tag ‘_Original.png’). (ZIP) [file pcbi.1010842.s025.zip › S6 Data/MSCs/MSC_AugIter-40_10Stretch_10Shift/0/MSC-2_3.png]

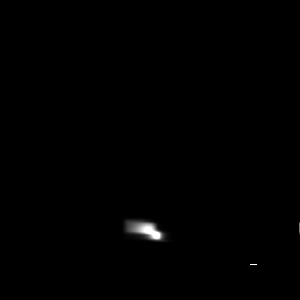

Supplement: S6 Data — The data is saved as *.png files. The subfolder entitled ‘0’ represent the undifferentiated cells while ‘1’ represent the differentiated MSCs. Every dataset also includes the original data, that were not affected by the augmentation algorithm (refer to tag ‘_Original.png’). (ZIP) [file pcbi.1010842.s025.zip › S6 Data/MSCs/MSC_AugIter-40_10Stretch_10Shift/0/MSC-2_30.png]

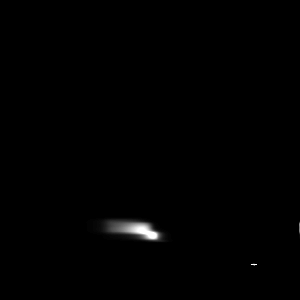

Supplement: S6 Data — The data is saved as *.png files. The subfolder entitled ‘0’ represent the undifferentiated cells while ‘1’ represent the differentiated MSCs. Every dataset also includes the original data, that were not affected by the augmentation algorithm (refer to tag ‘_Original.png’). (ZIP) [file pcbi.1010842.s025.zip › S6 Data/MSCs/MSC_AugIter-40_10Stretch_10Shift/0/MSC-2_31.png]

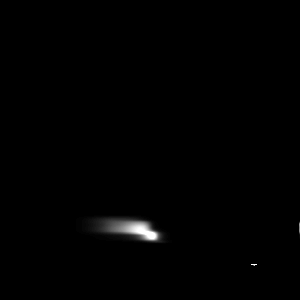

Supplement: S6 Data — The data is saved as *.png files. The subfolder entitled ‘0’ represent the undifferentiated cells while ‘1’ represent the differentiated MSCs. Every dataset also includes the original data, that were not affected by the augmentation algorithm (refer to tag ‘_Original.png’). (ZIP) [file pcbi.1010842.s025.zip › S6 Data/MSCs/MSC_AugIter-40_10Stretch_10Shift/0/MSC-2_32.png]

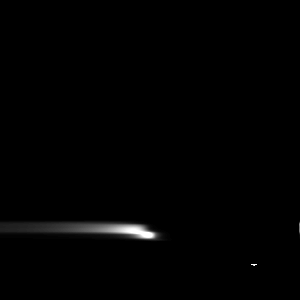

Supplement: S6 Data — The data is saved as *.png files. The subfolder entitled ‘0’ represent the undifferentiated cells while ‘1’ represent the differentiated MSCs. Every dataset also includes the original data, that were not affected by the augmentation algorithm (refer to tag ‘_Original.png’). (ZIP) [file pcbi.1010842.s025.zip › S6 Data/MSCs/MSC_AugIter-40_10Stretch_10Shift/0/MSC-2_33.png]

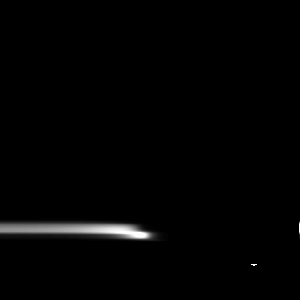

Supplement: S6 Data — The data is saved as *.png files. The subfolder entitled ‘0’ represent the undifferentiated cells while ‘1’ represent the differentiated MSCs. Every dataset also includes the original data, that were not affected by the augmentation algorithm (refer to tag ‘_Original.png’). (ZIP) [file pcbi.1010842.s025.zip › S6 Data/MSCs/MSC_AugIter-40_10Stretch_10Shift/0/MSC-2_34.png]

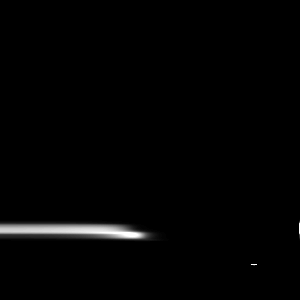

Supplement: S6 Data — The data is saved as *.png files. The subfolder entitled ‘0’ represent the undifferentiated cells while ‘1’ represent the differentiated MSCs. Every dataset also includes the original data, that were not affected by the augmentation algorithm (refer to tag ‘_Original.png’). (ZIP) [file pcbi.1010842.s025.zip › S6 Data/MSCs/MSC_AugIter-40_10Stretch_10Shift/0/MSC-2_35.png]

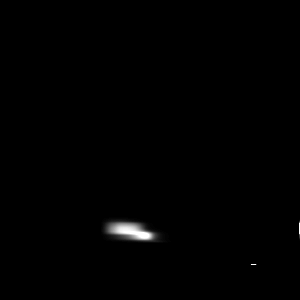

Supplement: S6 Data — The data is saved as *.png files. The subfolder entitled ‘0’ represent the undifferentiated cells while ‘1’ represent the differentiated MSCs. Every dataset also includes the original data, that were not affected by the augmentation algorithm (refer to tag ‘_Original.png’). (ZIP) [file pcbi.1010842.s025.zip › S6 Data/MSCs/MSC_AugIter-40_10Stretch_10Shift/0/MSC-2_36.png]

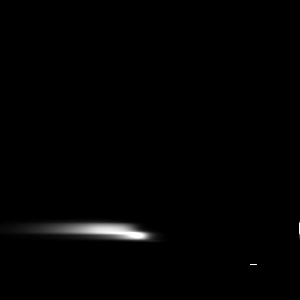

Supplement: S6 Data — The data is saved as *.png files. The subfolder entitled ‘0’ represent the undifferentiated cells while ‘1’ represent the differentiated MSCs. Every dataset also includes the original data, that were not affected by the augmentation algorithm (refer to tag ‘_Original.png’). (ZIP) [file pcbi.1010842.s025.zip › S6 Data/MSCs/MSC_AugIter-40_10Stretch_10Shift/0/MSC-2_37.png]

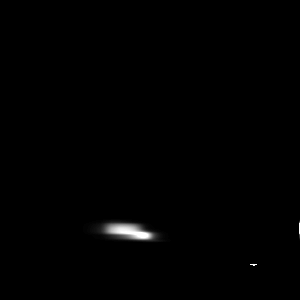

Supplement: S6 Data — The data is saved as *.png files. The subfolder entitled ‘0’ represent the undifferentiated cells while ‘1’ represent the differentiated MSCs. Every dataset also includes the original data, that were not affected by the augmentation algorithm (refer to tag ‘_Original.png’). (ZIP) [file pcbi.1010842.s025.zip › S6 Data/MSCs/MSC_AugIter-40_10Stretch_10Shift/0/MSC-2_38.png]

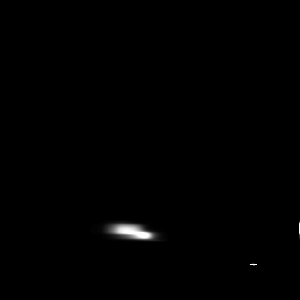

Supplement: S6 Data — The data is saved as *.png files. The subfolder entitled ‘0’ represent the undifferentiated cells while ‘1’ represent the differentiated MSCs. Every dataset also includes the original data, that were not affected by the augmentation algorithm (refer to tag ‘_Original.png’). (ZIP) [file pcbi.1010842.s025.zip › S6 Data/MSCs/MSC_AugIter-40_10Stretch_10Shift/0/MSC-2_39.png]

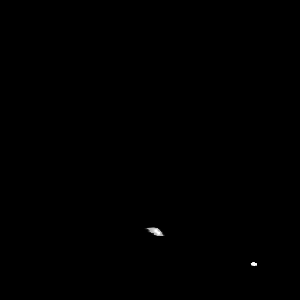

Supplement: S6 Data — The data is saved as *.png files. The subfolder entitled ‘0’ represent the undifferentiated cells while ‘1’ represent the differentiated MSCs. Every dataset also includes the original data, that were not affected by the augmentation algorithm (refer to tag ‘_Original.png’). (ZIP) [file pcbi.1010842.s025.zip › S6 Data/MSCs/MSC_AugIter-40_10Stretch_10Shift/0/MSC-2_4.png]

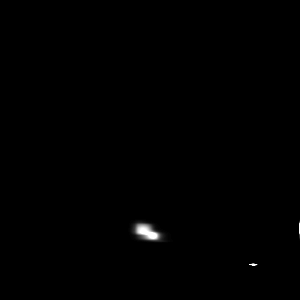

Supplement: S6 Data — The data is saved as *.png files. The subfolder entitled ‘0’ represent the undifferentiated cells while ‘1’ represent the differentiated MSCs. Every dataset also includes the original data, that were not affected by the augmentation algorithm (refer to tag ‘_Original.png’). (ZIP) [file pcbi.1010842.s025.zip › S6 Data/MSCs/MSC_AugIter-40_10Stretch_10Shift/0/MSC-2_40.png]

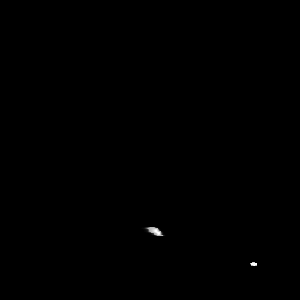

Supplement: S6 Data — The data is saved as *.png files. The subfolder entitled ‘0’ represent the undifferentiated cells while ‘1’ represent the differentiated MSCs. Every dataset also includes the original data, that were not affected by the augmentation algorithm (refer to tag ‘_Original.png’). (ZIP) [file pcbi.1010842.s025.zip › S6 Data/MSCs/MSC_AugIter-40_10Stretch_10Shift/0/MSC-2_5.png]

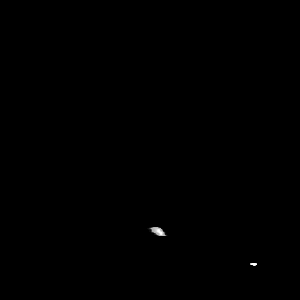

Supplement: S6 Data — The data is saved as *.png files. The subfolder entitled ‘0’ represent the undifferentiated cells while ‘1’ represent the differentiated MSCs. Every dataset also includes the original data, that were not affected by the augmentation algorithm (refer to tag ‘_Original.png’). (ZIP) [file pcbi.1010842.s025.zip › S6 Data/MSCs/MSC_AugIter-40_10Stretch_10Shift/0/MSC-2_6.png]

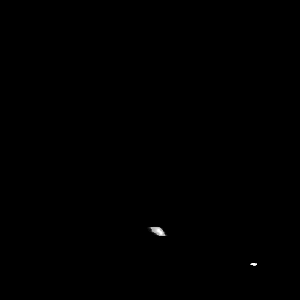

Supplement: S6 Data — The data is saved as *.png files. The subfolder entitled ‘0’ represent the undifferentiated cells while ‘1’ represent the differentiated MSCs. Every dataset also includes the original data, that were not affected by the augmentation algorithm (refer to tag ‘_Original.png’). (ZIP) [file pcbi.1010842.s025.zip › S6 Data/MSCs/MSC_AugIter-40_10Stretch_10Shift/0/MSC-2_7.png]

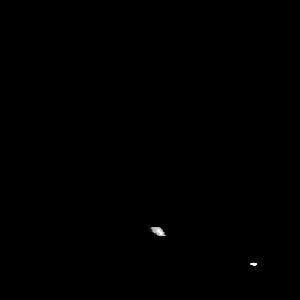

Supplement: S6 Data — The data is saved as *.png files. The subfolder entitled ‘0’ represent the undifferentiated cells while ‘1’ represent the differentiated MSCs. Every dataset also includes the original data, that were not affected by the augmentation algorithm (refer to tag ‘_Original.png’). (ZIP) [file pcbi.1010842.s025.zip › S6 Data/MSCs/MSC_AugIter-40_10Stretch_10Shift/0/MSC-2_8.png]

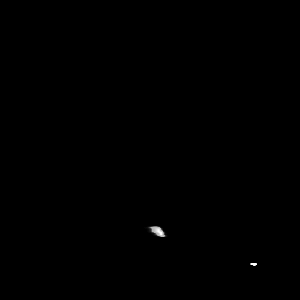

Supplement: S6 Data — The data is saved as *.png files. The subfolder entitled ‘0’ represent the undifferentiated cells while ‘1’ represent the differentiated MSCs. Every dataset also includes the original data, that were not affected by the augmentation algorithm (refer to tag ‘_Original.png’). (ZIP) [file pcbi.1010842.s025.zip › S6 Data/MSCs/MSC_AugIter-40_10Stretch_10Shift/0/MSC-2_9.png]

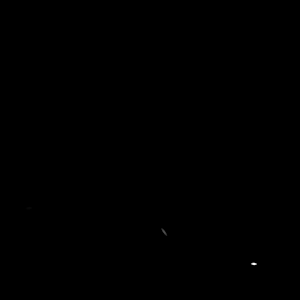

Supplement: S6 Data — The data is saved as *.png files. The subfolder entitled ‘0’ represent the undifferentiated cells while ‘1’ represent the differentiated MSCs. Every dataset also includes the original data, that were not affected by the augmentation algorithm (refer to tag ‘_Original.png’). (ZIP) [file pcbi.1010842.s025.zip › S6 Data/MSCs/MSC_AugIter-40_10Stretch_10Shift/0/MSC-2_Original.png]

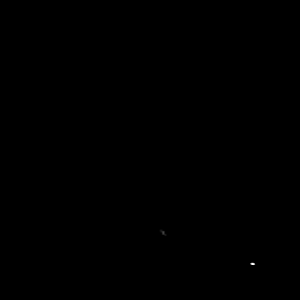

Supplement: S6 Data — The data is saved as *.png files. The subfolder entitled ‘0’ represent the undifferentiated cells while ‘1’ represent the differentiated MSCs. Every dataset also includes the original data, that were not affected by the augmentation algorithm (refer to tag ‘_Original.png’). (ZIP) [file pcbi.1010842.s025.zip › S6 Data/MSCs/MSC_AugIter-40_10Stretch_10Shift/0/MSC-3_1.png]

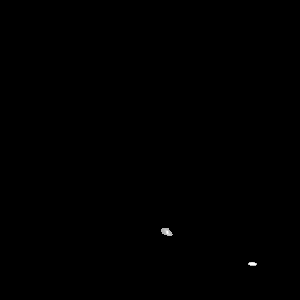

Supplement: S6 Data — The data is saved as *.png files. The subfolder entitled ‘0’ represent the undifferentiated cells while ‘1’ represent the differentiated MSCs. Every dataset also includes the original data, that were not affected by the augmentation algorithm (refer to tag ‘_Original.png’). (ZIP) [file pcbi.1010842.s025.zip › S6 Data/MSCs/MSC_AugIter-40_10Stretch_10Shift/0/MSC-3_10.png]

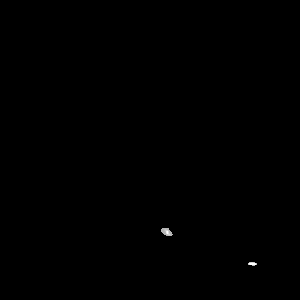

Supplement: S6 Data — The data is saved as *.png files. The subfolder entitled ‘0’ represent the undifferentiated cells while ‘1’ represent the differentiated MSCs. Every dataset also includes the original data, that were not affected by the augmentation algorithm (refer to tag ‘_Original.png’). (ZIP) [file pcbi.1010842.s025.zip › S6 Data/MSCs/MSC_AugIter-40_10Stretch_10Shift/0/MSC-3_11.png]

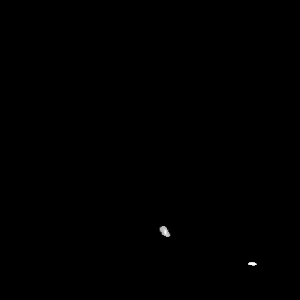

Supplement: S6 Data — The data is saved as *.png files. The subfolder entitled ‘0’ represent the undifferentiated cells while ‘1’ represent the differentiated MSCs. Every dataset also includes the original data, that were not affected by the augmentation algorithm (refer to tag ‘_Original.png’). (ZIP) [file pcbi.1010842.s025.zip › S6 Data/MSCs/MSC_AugIter-40_10Stretch_10Shift/0/MSC-3_12.png]

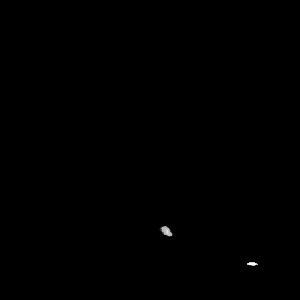

Supplement: S6 Data — The data is saved as *.png files. The subfolder entitled ‘0’ represent the undifferentiated cells while ‘1’ represent the differentiated MSCs. Every dataset also includes the original data, that were not affected by the augmentation algorithm (refer to tag ‘_Original.png’). (ZIP) [file pcbi.1010842.s025.zip › S6 Data/MSCs/MSC_AugIter-40_10Stretch_10Shift/0/MSC-3_13.png]

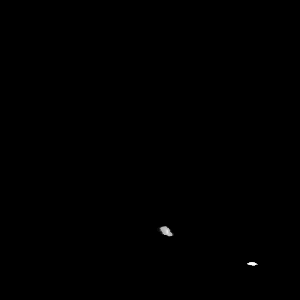

Supplement: S6 Data — The data is saved as *.png files. The subfolder entitled ‘0’ represent the undifferentiated cells while ‘1’ represent the differentiated MSCs. Every dataset also includes the original data, that were not affected by the augmentation algorithm (refer to tag ‘_Original.png’). (ZIP) [file pcbi.1010842.s025.zip › S6 Data/MSCs/MSC_AugIter-40_10Stretch_10Shift/0/MSC-3_14.png]

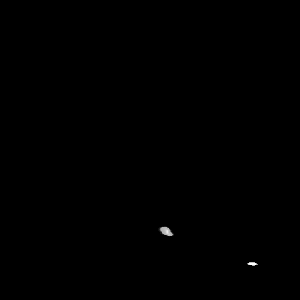

Supplement: S6 Data — The data is saved as *.png files. The subfolder entitled ‘0’ represent the undifferentiated cells while ‘1’ represent the differentiated MSCs. Every dataset also includes the original data, that were not affected by the augmentation algorithm (refer to tag ‘_Original.png’). (ZIP) [file pcbi.1010842.s025.zip › S6 Data/MSCs/MSC_AugIter-40_10Stretch_10Shift/0/MSC-3_15.png]

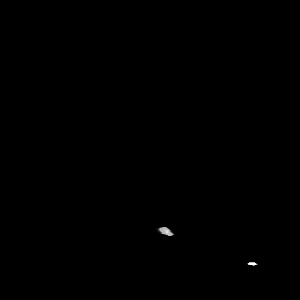

Supplement: S6 Data — The data is saved as *.png files. The subfolder entitled ‘0’ represent the undifferentiated cells while ‘1’ represent the differentiated MSCs. Every dataset also includes the original data, that were not affected by the augmentation algorithm (refer to tag ‘_Original.png’). (ZIP) [file pcbi.1010842.s025.zip › S6 Data/MSCs/MSC_AugIter-40_10Stretch_10Shift/0/MSC-3_16.png]

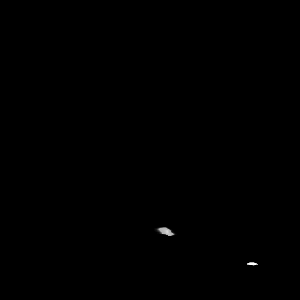

Supplement: S6 Data — The data is saved as *.png files. The subfolder entitled ‘0’ represent the undifferentiated cells while ‘1’ represent the differentiated MSCs. Every dataset also includes the original data, that were not affected by the augmentation algorithm (refer to tag ‘_Original.png’). (ZIP) [file pcbi.1010842.s025.zip › S6 Data/MSCs/MSC_AugIter-40_10Stretch_10Shift/0/MSC-3_17.png]

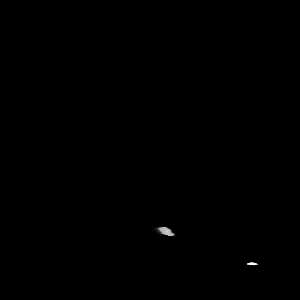

Supplement: S6 Data — The data is saved as *.png files. The subfolder entitled ‘0’ represent the undifferentiated cells while ‘1’ represent the differentiated MSCs. Every dataset also includes the original data, that were not affected by the augmentation algorithm (refer to tag ‘_Original.png’). (ZIP) [file pcbi.1010842.s025.zip › S6 Data/MSCs/MSC_AugIter-40_10Stretch_10Shift/0/MSC-3_18.png]

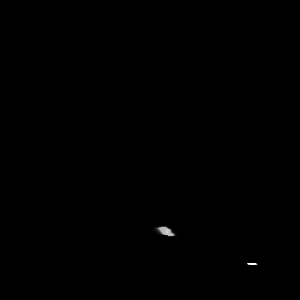

Supplement: S6 Data — The data is saved as *.png files. The subfolder entitled ‘0’ represent the undifferentiated cells while ‘1’ represent the differentiated MSCs. Every dataset also includes the original data, that were not affected by the augmentation algorithm (refer to tag ‘_Original.png’). (ZIP) [file pcbi.1010842.s025.zip › S6 Data/MSCs/MSC_AugIter-40_10Stretch_10Shift/0/MSC-3_19.png]

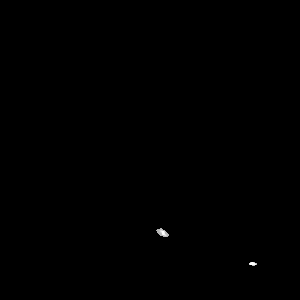

Supplement: S6 Data — The data is saved as *.png files. The subfolder entitled ‘0’ represent the undifferentiated cells while ‘1’ represent the differentiated MSCs. Every dataset also includes the original data, that were not affected by the augmentation algorithm (refer to tag ‘_Original.png’). (ZIP) [file pcbi.1010842.s025.zip › S6 Data/MSCs/MSC_AugIter-40_10Stretch_10Shift/0/MSC-3_2.png]

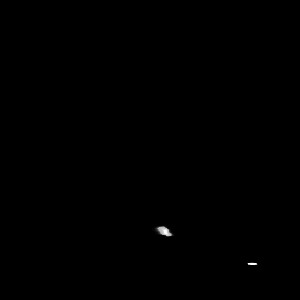

Supplement: S6 Data — The data is saved as *.png files. The subfolder entitled ‘0’ represent the undifferentiated cells while ‘1’ represent the differentiated MSCs. Every dataset also includes the original data, that were not affected by the augmentation algorithm (refer to tag ‘_Original.png’). (ZIP) [file pcbi.1010842.s025.zip › S6 Data/MSCs/MSC_AugIter-40_10Stretch_10Shift/0/MSC-3_20.png]

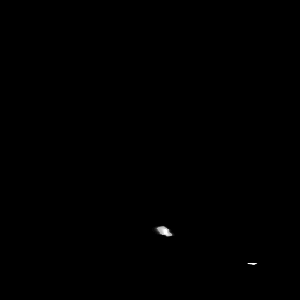

Supplement: S6 Data — The data is saved as *.png files. The subfolder entitled ‘0’ represent the undifferentiated cells while ‘1’ represent the differentiated MSCs. Every dataset also includes the original data, that were not affected by the augmentation algorithm (refer to tag ‘_Original.png’). (ZIP) [file pcbi.1010842.s025.zip › S6 Data/MSCs/MSC_AugIter-40_10Stretch_10Shift/0/MSC-3_21.png]

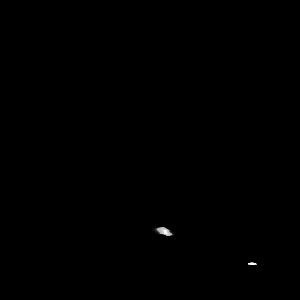

Supplement: S6 Data — The data is saved as *.png files. The subfolder entitled ‘0’ represent the undifferentiated cells while ‘1’ represent the differentiated MSCs. Every dataset also includes the original data, that were not affected by the augmentation algorithm (refer to tag ‘_Original.png’). (ZIP) [file pcbi.1010842.s025.zip › S6 Data/MSCs/MSC_AugIter-40_10Stretch_10Shift/0/MSC-3_22.png]

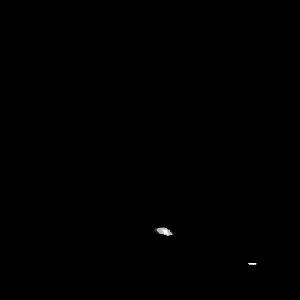

Supplement: S6 Data — The data is saved as *.png files. The subfolder entitled ‘0’ represent the undifferentiated cells while ‘1’ represent the differentiated MSCs. Every dataset also includes the original data, that were not affected by the augmentation algorithm (refer to tag ‘_Original.png’). (ZIP) [file pcbi.1010842.s025.zip › S6 Data/MSCs/MSC_AugIter-40_10Stretch_10Shift/0/MSC-3_23.png]

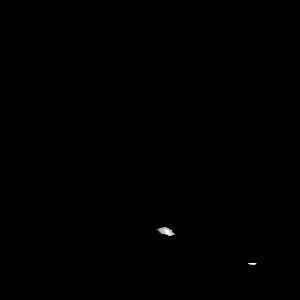

Supplement: S6 Data — The data is saved as *.png files. The subfolder entitled ‘0’ represent the undifferentiated cells while ‘1’ represent the differentiated MSCs. Every dataset also includes the original data, that were not affected by the augmentation algorithm (refer to tag ‘_Original.png’). (ZIP) [file pcbi.1010842.s025.zip › S6 Data/MSCs/MSC_AugIter-40_10Stretch_10Shift/0/MSC-3_24.png]

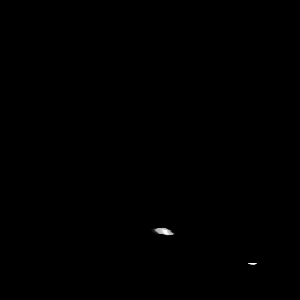

Supplement: S6 Data — The data is saved as *.png files. The subfolder entitled ‘0’ represent the undifferentiated cells while ‘1’ represent the differentiated MSCs. Every dataset also includes the original data, that were not affected by the augmentation algorithm (refer to tag ‘_Original.png’). (ZIP) [file pcbi.1010842.s025.zip › S6 Data/MSCs/MSC_AugIter-40_10Stretch_10Shift/0/MSC-3_25.png]

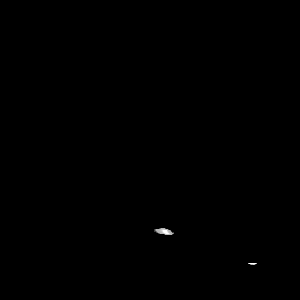

Supplement: S6 Data — The data is saved as *.png files. The subfolder entitled ‘0’ represent the undifferentiated cells while ‘1’ represent the differentiated MSCs. Every dataset also includes the original data, that were not affected by the augmentation algorithm (refer to tag ‘_Original.png’). (ZIP) [file pcbi.1010842.s025.zip › S6 Data/MSCs/MSC_AugIter-40_10Stretch_10Shift/0/MSC-3_26.png]

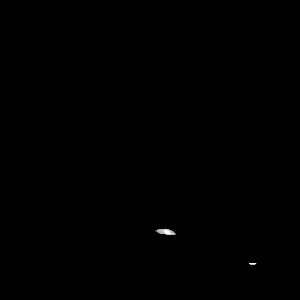

Supplement: S6 Data — The data is saved as *.png files. The subfolder entitled ‘0’ represent the undifferentiated cells while ‘1’ represent the differentiated MSCs. Every dataset also includes the original data, that were not affected by the augmentation algorithm (refer to tag ‘_Original.png’). (ZIP) [file pcbi.1010842.s025.zip › S6 Data/MSCs/MSC_AugIter-40_10Stretch_10Shift/0/MSC-3_27.png]

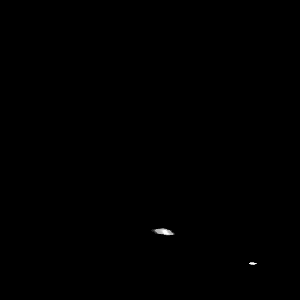

Supplement: S6 Data — The data is saved as *.png files. The subfolder entitled ‘0’ represent the undifferentiated cells while ‘1’ represent the differentiated MSCs. Every dataset also includes the original data, that were not affected by the augmentation algorithm (refer to tag ‘_Original.png’). (ZIP) [file pcbi.1010842.s025.zip › S6 Data/MSCs/MSC_AugIter-40_10Stretch_10Shift/0/MSC-3_28.png]

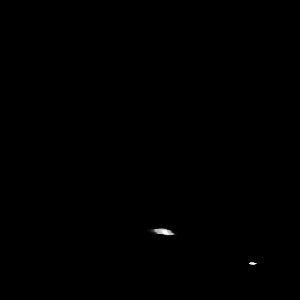

Supplement: S6 Data — The data is saved as *.png files. The subfolder entitled ‘0’ represent the undifferentiated cells while ‘1’ represent the differentiated MSCs. Every dataset also includes the original data, that were not affected by the augmentation algorithm (refer to tag ‘_Original.png’). (ZIP) [file pcbi.1010842.s025.zip › S6 Data/MSCs/MSC_AugIter-40_10Stretch_10Shift/0/MSC-3_29.png]

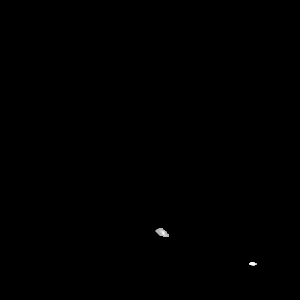

Supplement: S6 Data — The data is saved as *.png files. The subfolder entitled ‘0’ represent the undifferentiated cells while ‘1’ represent the differentiated MSCs. Every dataset also includes the original data, that were not affected by the augmentation algorithm (refer to tag ‘_Original.png’). (ZIP) [file pcbi.1010842.s025.zip › S6 Data/MSCs/MSC_AugIter-40_10Stretch_10Shift/0/MSC-3_3.png]

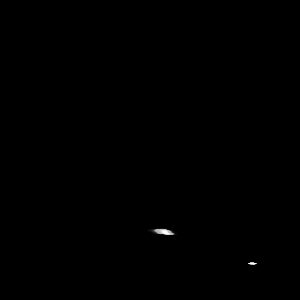

Supplement: S6 Data — The data is saved as *.png files. The subfolder entitled ‘0’ represent the undifferentiated cells while ‘1’ represent the differentiated MSCs. Every dataset also includes the original data, that were not affected by the augmentation algorithm (refer to tag ‘_Original.png’). (ZIP) [file pcbi.1010842.s025.zip › S6 Data/MSCs/MSC_AugIter-40_10Stretch_10Shift/0/MSC-3_30.png]

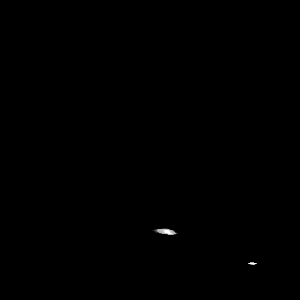

Supplement: S6 Data — The data is saved as *.png files. The subfolder entitled ‘0’ represent the undifferentiated cells while ‘1’ represent the differentiated MSCs. Every dataset also includes the original data, that were not affected by the augmentation algorithm (refer to tag ‘_Original.png’). (ZIP) [file pcbi.1010842.s025.zip › S6 Data/MSCs/MSC_AugIter-40_10Stretch_10Shift/0/MSC-3_31.png]

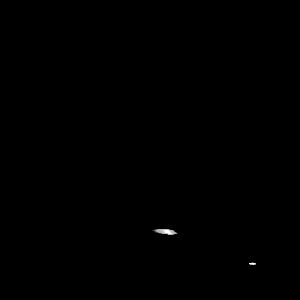

Supplement: S6 Data — The data is saved as *.png files. The subfolder entitled ‘0’ represent the undifferentiated cells while ‘1’ represent the differentiated MSCs. Every dataset also includes the original data, that were not affected by the augmentation algorithm (refer to tag ‘_Original.png’). (ZIP) [file pcbi.1010842.s025.zip › S6 Data/MSCs/MSC_AugIter-40_10Stretch_10Shift/0/MSC-3_32.png]

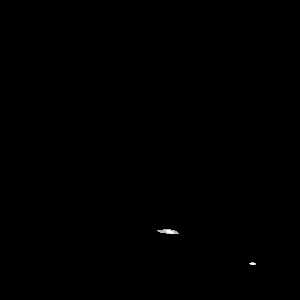

Supplement: S6 Data — The data is saved as *.png files. The subfolder entitled ‘0’ represent the undifferentiated cells while ‘1’ represent the differentiated MSCs. Every dataset also includes the original data, that were not affected by the augmentation algorithm (refer to tag ‘_Original.png’). (ZIP) [file pcbi.1010842.s025.zip › S6 Data/MSCs/MSC_AugIter-40_10Stretch_10Shift/0/MSC-3_33.png]

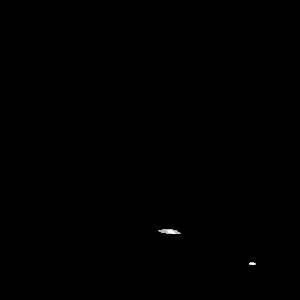

Supplement: S6 Data — The data is saved as *.png files. The subfolder entitled ‘0’ represent the undifferentiated cells while ‘1’ represent the differentiated MSCs. Every dataset also includes the original data, that were not affected by the augmentation algorithm (refer to tag ‘_Original.png’). (ZIP) [file pcbi.1010842.s025.zip › S6 Data/MSCs/MSC_AugIter-40_10Stretch_10Shift/0/MSC-3_34.png]

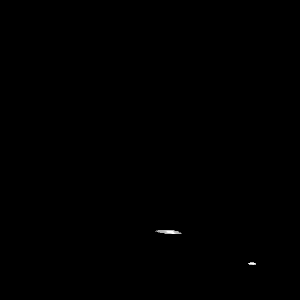

Supplement: S6 Data — The data is saved as *.png files. The subfolder entitled ‘0’ represent the undifferentiated cells while ‘1’ represent the differentiated MSCs. Every dataset also includes the original data, that were not affected by the augmentation algorithm (refer to tag ‘_Original.png’). (ZIP) [file pcbi.1010842.s025.zip › S6 Data/MSCs/MSC_AugIter-40_10Stretch_10Shift/0/MSC-3_35.png]

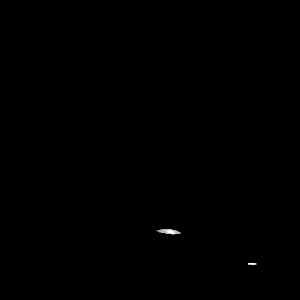

Supplement: S6 Data — The data is saved as *.png files. The subfolder entitled ‘0’ represent the undifferentiated cells while ‘1’ represent the differentiated MSCs. Every dataset also includes the original data, that were not affected by the augmentation algorithm (refer to tag ‘_Original.png’). (ZIP) [file pcbi.1010842.s025.zip › S6 Data/MSCs/MSC_AugIter-40_10Stretch_10Shift/0/MSC-3_36.png]

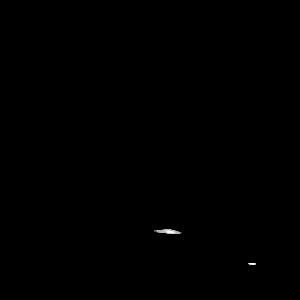

Supplement: S6 Data — The data is saved as *.png files. The subfolder entitled ‘0’ represent the undifferentiated cells while ‘1’ represent the differentiated MSCs. Every dataset also includes the original data, that were not affected by the augmentation algorithm (refer to tag ‘_Original.png’). (ZIP) [file pcbi.1010842.s025.zip › S6 Data/MSCs/MSC_AugIter-40_10Stretch_10Shift/0/MSC-3_37.png]

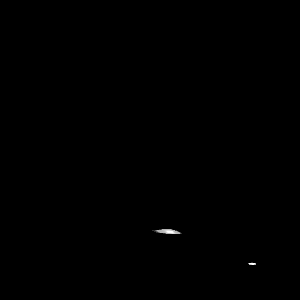

Supplement: S6 Data — The data is saved as *.png files. The subfolder entitled ‘0’ represent the undifferentiated cells while ‘1’ represent the differentiated MSCs. Every dataset also includes the original data, that were not affected by the augmentation algorithm (refer to tag ‘_Original.png’). (ZIP) [file pcbi.1010842.s025.zip › S6 Data/MSCs/MSC_AugIter-40_10Stretch_10Shift/0/MSC-3_38.png]

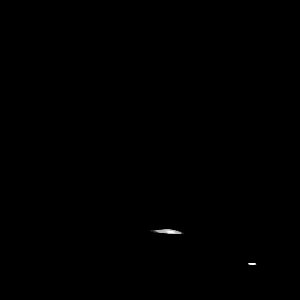

Supplement: S6 Data — The data is saved as *.png files. The subfolder entitled ‘0’ represent the undifferentiated cells while ‘1’ represent the differentiated MSCs. Every dataset also includes the original data, that were not affected by the augmentation algorithm (refer to tag ‘_Original.png’). (ZIP) [file pcbi.1010842.s025.zip › S6 Data/MSCs/MSC_AugIter-40_10Stretch_10Shift/0/MSC-3_39.png]

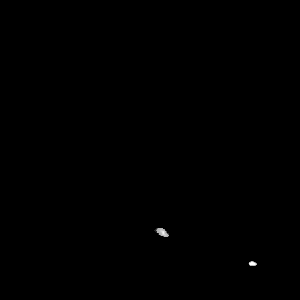

Supplement: S6 Data — The data is saved as *.png files. The subfolder entitled ‘0’ represent the undifferentiated cells while ‘1’ represent the differentiated MSCs. Every dataset also includes the original data, that were not affected by the augmentation algorithm (refer to tag ‘_Original.png’). (ZIP) [file pcbi.1010842.s025.zip › S6 Data/MSCs/MSC_AugIter-40_10Stretch_10Shift/0/MSC-3_4.png]

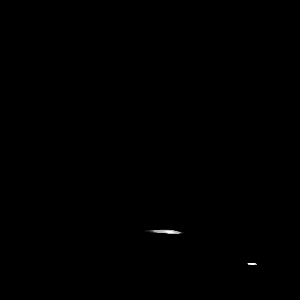

Supplement: S6 Data — The data is saved as *.png files. The subfolder entitled ‘0’ represent the undifferentiated cells while ‘1’ represent the differentiated MSCs. Every dataset also includes the original data, that were not affected by the augmentation algorithm (refer to tag ‘_Original.png’). (ZIP) [file pcbi.1010842.s025.zip › S6 Data/MSCs/MSC_AugIter-40_10Stretch_10Shift/0/MSC-3_40.png]

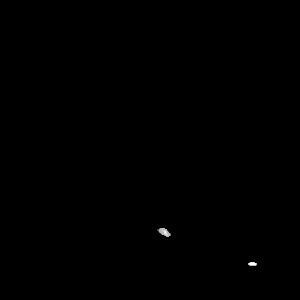

Supplement: S6 Data — The data is saved as *.png files. The subfolder entitled ‘0’ represent the undifferentiated cells while ‘1’ represent the differentiated MSCs. Every dataset also includes the original data, that were not affected by the augmentation algorithm (refer to tag ‘_Original.png’). (ZIP) [file pcbi.1010842.s025.zip › S6 Data/MSCs/MSC_AugIter-40_10Stretch_10Shift/0/MSC-3_5.png]

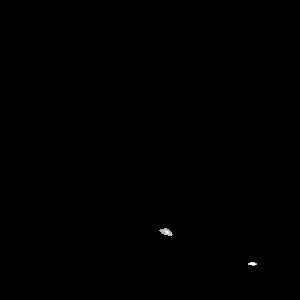

Supplement: S6 Data — The data is saved as *.png files. The subfolder entitled ‘0’ represent the undifferentiated cells while ‘1’ represent the differentiated MSCs. Every dataset also includes the original data, that were not affected by the augmentation algorithm (refer to tag ‘_Original.png’). (ZIP) [file pcbi.1010842.s025.zip › S6 Data/MSCs/MSC_AugIter-40_10Stretch_10Shift/0/MSC-3_6.png]

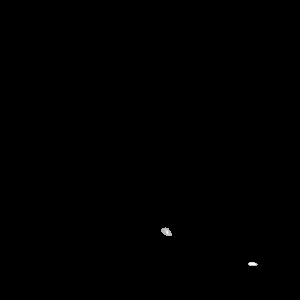

Supplement: S6 Data — The data is saved as *.png files. The subfolder entitled ‘0’ represent the undifferentiated cells while ‘1’ represent the differentiated MSCs. Every dataset also includes the original data, that were not affected by the augmentation algorithm (refer to tag ‘_Original.png’). (ZIP) [file pcbi.1010842.s025.zip › S6 Data/MSCs/MSC_AugIter-40_10Stretch_10Shift/0/MSC-3_7.png]

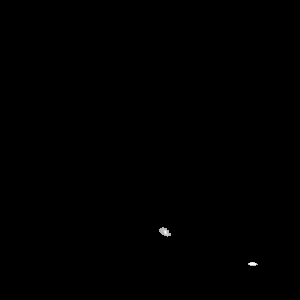

Supplement: S6 Data — The data is saved as *.png files. The subfolder entitled ‘0’ represent the undifferentiated cells while ‘1’ represent the differentiated MSCs. Every dataset also includes the original data, that were not affected by the augmentation algorithm (refer to tag ‘_Original.png’). (ZIP) [file pcbi.1010842.s025.zip › S6 Data/MSCs/MSC_AugIter-40_10Stretch_10Shift/0/MSC-3_8.png]

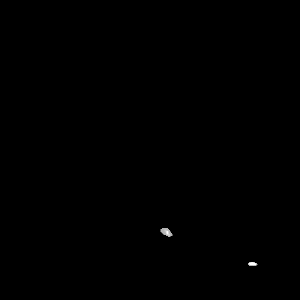

Supplement: S6 Data — The data is saved as *.png files. The subfolder entitled ‘0’ represent the undifferentiated cells while ‘1’ represent the differentiated MSCs. Every dataset also includes the original data, that were not affected by the augmentation algorithm (refer to tag ‘_Original.png’). (ZIP) [file pcbi.1010842.s025.zip › S6 Data/MSCs/MSC_AugIter-40_10Stretch_10Shift/0/MSC-3_9.png]

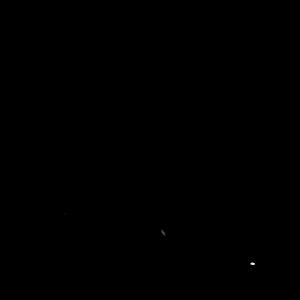

Supplement: S6 Data — The data is saved as *.png files. The subfolder entitled ‘0’ represent the undifferentiated cells while ‘1’ represent the differentiated MSCs. Every dataset also includes the original data, that were not affected by the augmentation algorithm (refer to tag ‘_Original.png’). (ZIP) [file pcbi.1010842.s025.zip › S6 Data/MSCs/MSC_AugIter-40_10Stretch_10Shift/0/MSC-3_Original.png]

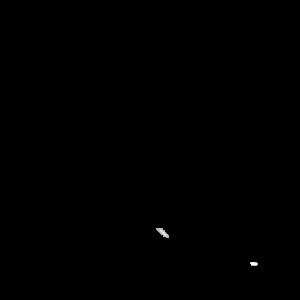

Supplement: S6 Data — The data is saved as *.png files. The subfolder entitled ‘0’ represent the undifferentiated cells while ‘1’ represent the differentiated MSCs. Every dataset also includes the original data, that were not affected by the augmentation algorithm (refer to tag ‘_Original.png’). (ZIP) [file pcbi.1010842.s025.zip › S6 Data/MSCs/MSC_AugIter-40_10Stretch_10Shift/0/MSC-7_1.png]

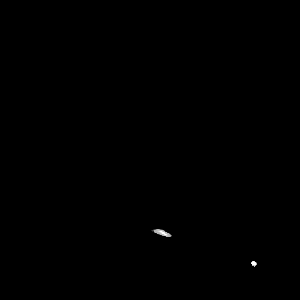

Supplement: S6 Data — The data is saved as *.png files. The subfolder entitled ‘0’ represent the undifferentiated cells while ‘1’ represent the differentiated MSCs. Every dataset also includes the original data, that were not affected by the augmentation algorithm (refer to tag ‘_Original.png’). (ZIP) [file pcbi.1010842.s025.zip › S6 Data/MSCs/MSC_AugIter-40_10Stretch_10Shift/0/MSC-7_10.png]

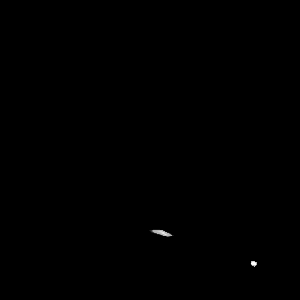

Supplement: S6 Data — The data is saved as *.png files. The subfolder entitled ‘0’ represent the undifferentiated cells while ‘1’ represent the differentiated MSCs. Every dataset also includes the original data, that were not affected by the augmentation algorithm (refer to tag ‘_Original.png’). (ZIP) [file pcbi.1010842.s025.zip › S6 Data/MSCs/MSC_AugIter-40_10Stretch_10Shift/0/MSC-7_11.png]

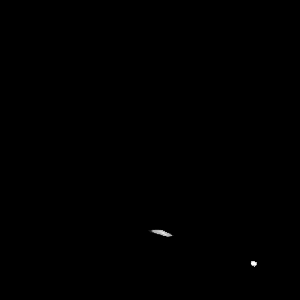

Supplement: S6 Data — The data is saved as *.png files. The subfolder entitled ‘0’ represent the undifferentiated cells while ‘1’ represent the differentiated MSCs. Every dataset also includes the original data, that were not affected by the augmentation algorithm (refer to tag ‘_Original.png’). (ZIP) [file pcbi.1010842.s025.zip › S6 Data/MSCs/MSC_AugIter-40_10Stretch_10Shift/0/MSC-7_12.png]

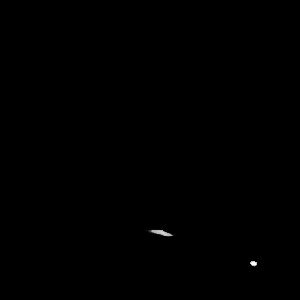

Supplement: S6 Data — The data is saved as *.png files. The subfolder entitled ‘0’ represent the undifferentiated cells while ‘1’ represent the differentiated MSCs. Every dataset also includes the original data, that were not affected by the augmentation algorithm (refer to tag ‘_Original.png’). (ZIP) [file pcbi.1010842.s025.zip › S6 Data/MSCs/MSC_AugIter-40_10Stretch_10Shift/0/MSC-7_13.png]
